# Efficacy of dietary polyphenol supplement in patients with non-alcoholic fatty liver disease: a network meta-analysis

**DOI:** 10.3389/fnut.2025.1582861

**Published:** 2025-05-09

**Authors:** Xiao-cui Wang, Li Song, Xin-han Wang

**Affiliations:** ^1^Ningxia Regional Key Laboratory of Integrated Traditional Chinese and Western Medicine for Prevention and Treatment of Regional High Incidence Disease, Ningxia Medical University, Yinchuan, China; ^2^Key Laboratory of Ningxia Ethnomedicine Modernization, Ministry of Education, Ningxia Medical University, Yinchuan, China; ^3^Department of Nephrology, Affiliated Hospital of Traditional Chinese Medicine, Ningxia Medical University, Yinchuan, China

**Keywords:** non-alcoholic fatty liver disease, dietary polyphenol, network meta-analysis, randomized controlled trials, metabolic indices, catechin

## Abstract

**Background:**

Non-alcoholic fatty liver disease (NAFLD) has become a public health issue worldwide. Dietary polyphenols are naturally occurring plant active ingredients and are widely employed in the treatment of NAFLD. However, the therapeutic effect is still controversial. In this study, a network meta-analysis (NMA) was performed to appraise the effects of various polyphenols on metabolic indices of NAFLD.

**Methods:**

PubMed, Embase, the Cochrane Library, and Web of Science were retrieved for English studies on dietary polyphenols in the treatment of NAFLD. Outcome measures were extracted from the included studies and compared using a Bayesian NMA model, encompassing body mass index (BMI), alanine aminotransferase (ALT), aspartate aminotransferase (AST), triglycerides (TG), total cholesterol (TC), high-density lipoprotein cholesterol (HDL-C), low-density lipoprotein cholesterol (LDL-C), and necrosis factor alpha (TNF-α).

**Results:**

In total, 54 randomized controlled trials (RCTs) were included in this study, including 3,132 participants. It involved 13 single (or combined) dietary polyphenols. Naringenin could reduce serum TC (surface under the cumulative ranking curve: 94.59%) and TG (99.00%) in NAFLD patients. Catechin could decrease BMI (77.74%) and serum ALT (94.21%), AST (93.56%), TC (92.26%), and increase HDL-C (93.72%). Dihydromyricetin (DHM) was effective in reducing serum LDL-C (73.22%), and quercetin decreased serum TNF-α (99.47%).

**Conclusion:**

Catechin may be the most appropriate dietary polyphenol supplement for NAFLD. Future studies should incorporate more RCTs to further validate the efficacy of dietary polyphenols (like DHM and quercetin), which are limited in sample sizes, in treating NAFLD. On the other hand, it is essential to investigate improvements in the bioavailability of these dietary polyphenols and to clarify their safety profiles.

## 1 Introduction

Non-alcoholic fatty liver disease (NAFLD) is the condition of excessive liver fat accumulation, excluding secondary causes and excessive alcohol intake, including two pathologies with various prognoses: non-alcoholic fatty liver and non-alcoholic steatohepatitis (NASH) (1). NAFLD is generally believed to be associated with an unhealthy lifestyle, including a high-fat, high-sugar diet and sedentary behavior (1). Its pathogenesis is based on the “multiple hit” hypothesis, i.e., it is the result of multiple injuries (such as oxidative stress, lipotoxicity, and inflammatory state) (2). NAFLD is the most widespread cause of chronic liver disease across the globe. Between 1990 and 2019, the global prevalence of NAFLD was 30.1%, with 16.02% for NASH (3). Over the last 30 years, NAFLD prevalence has grown by 50.4% (3). During the 1999–2019 survey period in Asia, Mainland China had the highest NAFLD incidence rate, at 63 cases per 1,000 person-years (4). NAFLD patients are 1.9 times more likely to develop cancer than the general population (5). Extrahepatic cancers like uterine and breast cancer have an incidence rate more than eight times higher than that of hepatocellular carcinoma (HCC) (6). In the U.S., NASH is the foremost cause of end-stage liver disease and the second most frequent indication for liver transplantation (7). Since the onset of NAFLD is closely linked to cardiometabolic risk factors, such as type 2 diabetes mellitus (T2DM) and obesity, it has gained widespread attention (8). NAFLD is now a global public health issue and burden, and this burden is escalating quickly (3, 9).

An encouraging development is that in the first half of 2024, the U.S. Food and Drug Administration (FDA) granted approval for resmetirom (Rezdiffra^™^) as a treatment for adults with non-cirrhotic NASH and liver fibrosis (10), offering new hope for NAFLD treatment. The FDA states that this medication should be used together with diet and exercise, meaning that lifestyle changes remain the key approach in treating NAFLD. Combining the Mediterranean diet (MD), caloric restriction, and moderate- to high-intensity aerobic exercise/resistance training has proven effective in improving metabolic measures of NAFLD (11). However, the absence of long-term studies on their impact on the natural progression of NAFLD has prompted the exploration of alternative treatment approaches. Natural products are chemical substances with pharmacological or biological properties naturally produced by living organisms like plants, insects, animals, aquatic organisms and microorganisms (12), which are currently a hot research area for the treatment of diseases. Polyphenols are a class of plant-derived compounds, polyphenolic substances or isolated polyphenol monomers extracted from dietary substances or traditional medicines, which belong to the natural product paradigm. Dietary polyphenols, a class of plant-derived compounds found in fruits, tea, and vegetables, exhibit anti-inflammatory and antioxidant properties that can aid in improving the metabolic disturbances in NAFLD (13). A meta-analysis has examined the therapeutic effects of eight common dietary polyphenols, including curcumin, on NAFLD (14), providing guidance for the clinical selection of dietary polyphenols in NAFLD treatment. However, due to the wide variety of dietary polyphenols, most randomized controlled trials (RCTs) have primarily focused on comparing the efficacy differences between various polyphenols and placebos, without direct comparisons among polyphenols. This gap in evidence can be bridged through network meta-analysis (NMA).

The NMA is able to simultaneously compare multiple interventions not directly compared in RCTs and rank the effectiveness of the interventions (15). Hence, this study intends to compare the efficacy of various single (or combined) dietary polyphenol supplements in the treatment of NAFLD, like curcumin, resveratrol, anthocyanin, silymarin, catechin, chlorogenic acid (CA), ellagic acid (EA), genistin/genistein, naringenin, dihydromyricetin (DHM), hesperidin, quercetin, and gallic acid (GA) and CA, providing evidence-based guidance for both clinicians and patients in selecting appropriate dietary polyphenol therapies for NAFLD.

## 2 Methods

### 2.1 Registration

This study was in line with the preferred reporting items for systematic reviews and meta-analyses extension statement for reporting of systematic reviews incorporating network meta-analyses of health care interventions (16) and was registered in the international prospective register of systematic reviews^1^ with the registration number CRD42024591985.

### 2.2 Literature search strategy

Studies were retrieved from four electronic databases (PubMed, Embase, the Cochrane Library, and Web of Science) up to May 14, 2024. The keywords for the search were “polyphenol,” “phenolic acid,” “silymarin,” “anthocyanidin,” “hesperidin,” “resveratrol,” “nonalcoholic fatty liver,” “non-alcoholic fatty liver disease,” and “NAFLD.” The search formula employed was [(polyphenol) OR (phenolic acid) OR (silymarin) OR (anthocyanidin) OR (hesperidin) OR (resveratrol)] AND [(nonalcoholic fatty liver) OR (non-alcoholic fatty liver disease) OR (NAFLD)]. The detailed search strategy is available in Supplementary material 1.

### 2.3 Inclusion criteria

Population (P): (i) Sex was not limited; (ii) age ≥18 years; (iii) diagnosis of NAFLD confirmed by imaging or liver tissue biopsy.

Intervention (I): The intervention was to employ at least one dietary polyphenol available in the Phenol-Explorer 3.6 database (database on polyphenol content in foods).^2^

Comparison (C): The control intervention was placebo or any of the dietary polyphenols available in the Phenol-Explorer 3.6 database.

Outcomes (O): At least one of the following outcomes must be included: anthropometric measures, including weight, body mass index (BMI), waist circumference (WC), hip circumference (HC), waist-to-hip ratio (WHR), systolic blood pressure (SBP), and diastolic blood pressure (DBP); liver function measures, such as alanine aminotransferase (ALT), aspartate aminotransferase (AST), alkaline phosphatase (ALP), and gamma-glutamyl transferase (GGT); blood lipid metabolism measures, like triglyceride (TG), total cholesterol (TC), high-density lipoprotein cholesterol (HDL-C), and low-density lipoprotein cholesterol (LDL-C); insulin resistance (IR) measures, including fasting blood glucose (FBG), insulin, and homeostasis model assessment of IR; inflammation markers, like necrosis factor alpha (TNF-α).

Study design (S): RCTs.

The language was limited to English.

### 2.4 Exclusion criteria

(i) Studies with duplicate publications, repeated populations, and unavailable outcome measures.
(ii) Studies focusing on NAFLD with diabetes mellitus (DM) and other types of chronic liver diseases, such as viral hepatitis.
(iii) Studies where the intervention involved a particular food but did not clearly define the dietary polyphenol active ingredients, or where the list of these ingredients was too extensive for mechanism analysis; or studies that included dietary polyphenol supplements alongside western drugs (such metformin) or antioxidants (such as vitamin E).
(iv) Studies excluding the outcome measures covered in this study.
(v) Studies with insufficient data.
(vi) Animal or cell experiments, case reports, scientific experiment programs, reviews, letters, editorials, and conference papers.

### 2.5 Data extraction

Study screening and management were carried out by means of Endnote X9 software. Two authors (XiaW and LS) initially excluded studies that did not meet the inclusion criteria based on the titles and abstracts, then further searched for and read the full texts to make a final decision on inclusion. Data extraction was independently carried out by the two authors using a pre-created standardized electronic form, where the following key data were extracted and recorded: dietary polyphenols, title, first author, region, publication year, sample size, sex ratio, age, intervention, treatment duration, and outcome measures. To guarantee the accuracy of the data, all extracted data were input into Excel. Any disputed issues during the data extraction process were resolved through discussion between the two authors, with the involvement of a third author (XinW) when necessary.

### 2.6 Quality evaluation

Using the Cochrane risk of bias tool (ROB 2.0), the quality of each RCT included was appraised sequentially in five areas (17): (i) bias from the randomization process, (ii) bias from the intended intervention-intervention allocation, (iii) bias for missing outcome data, (iv) bias for outcome measurement, and (v) bias for selective reporting of results. The relevant response options were selected for each domain namely: yes, probably yes, probably no, no, and no information. These options were distinctly categorized as low risk of bias (ROB), moderate ROB, and high ROB. In the final quality evaluation, a study was deemed to have a low risk of bias (ROB) if all five domains indicated low ROB. If no more than three domains showed some ROB, it was classified as having a moderate ROB. A study with any domain identified as high ROB was considered to have a high ROB. The work was independently done by two authors (XiaW and XinW), and any differences were settled with a third author (LS).

### 2.7 Statistical analysis

All variables were expressed as mean ± standard deviation, with effect sizes measured by means of weighted mean difference (WMD) or standardized mean difference. When a study employed the same dietary polyphenol intervention with varying doses, only noticeable efficacy data was included in the NMA. When a study had two dietary polyphenol interventions, they were considered separate studies and included in the NMA. If certain studies were potentially or definitively derived from the same clinical trial registry, they may be included in the NMA, provided that duplicate outcome measures were excluded.

In this study, the Markov Chain Monte Carlo method was leveraged to create a Bayesian NMA model (18), which was iterated to estimate the relative efficacy of various dietary polyphenols. During testing, the model chain was set to 4, annealing to 10,000, iterations to 50,000, with a step size of 1 and an initial value of 2.5. This procedure was designed to obtain the posterior distribution.

To choose an appropriate effect model, heterogeneity among the included studies was appraised employing the *I*^2^ statistic. If *I*^2^ ≤ 50%, indicating moderate heterogeneity across studies (19), the Bayesian fixed-effect NMA model was employed. In contrast, the Bayesian random-effects NMA model was applied (20). In this way, the overall effect size could be estimated more accurately. Since the study results did not form a closed loop, an inconsistency test was not executed.

Simultaneously, a network diagram was plotted to intuitively display the links among various polyphenol interventions for NAFLD. Finally, the surface under the cumulative ranking curve (SUCRA) was calculated (21), allowing for a ranking of the therapeutic effects of each polyphenol. Additionally, a publication bias test was executed. Funnel plots were leveraged to appraise the potential for publication bias (22), thereby ensuring the impartiality and thoroughness of the analysis results.

R (version 4.3.2) was leveraged to build the Bayesian NMA model, perform heterogeneity tests, and calculate SUCRA. Network diagrams and funnel plots were created by means of Stata (version 15.1). *p* < 0.05 was deemed statistically significant.

## 3 Results

### 3.1 Study selection

**Figure 1:**
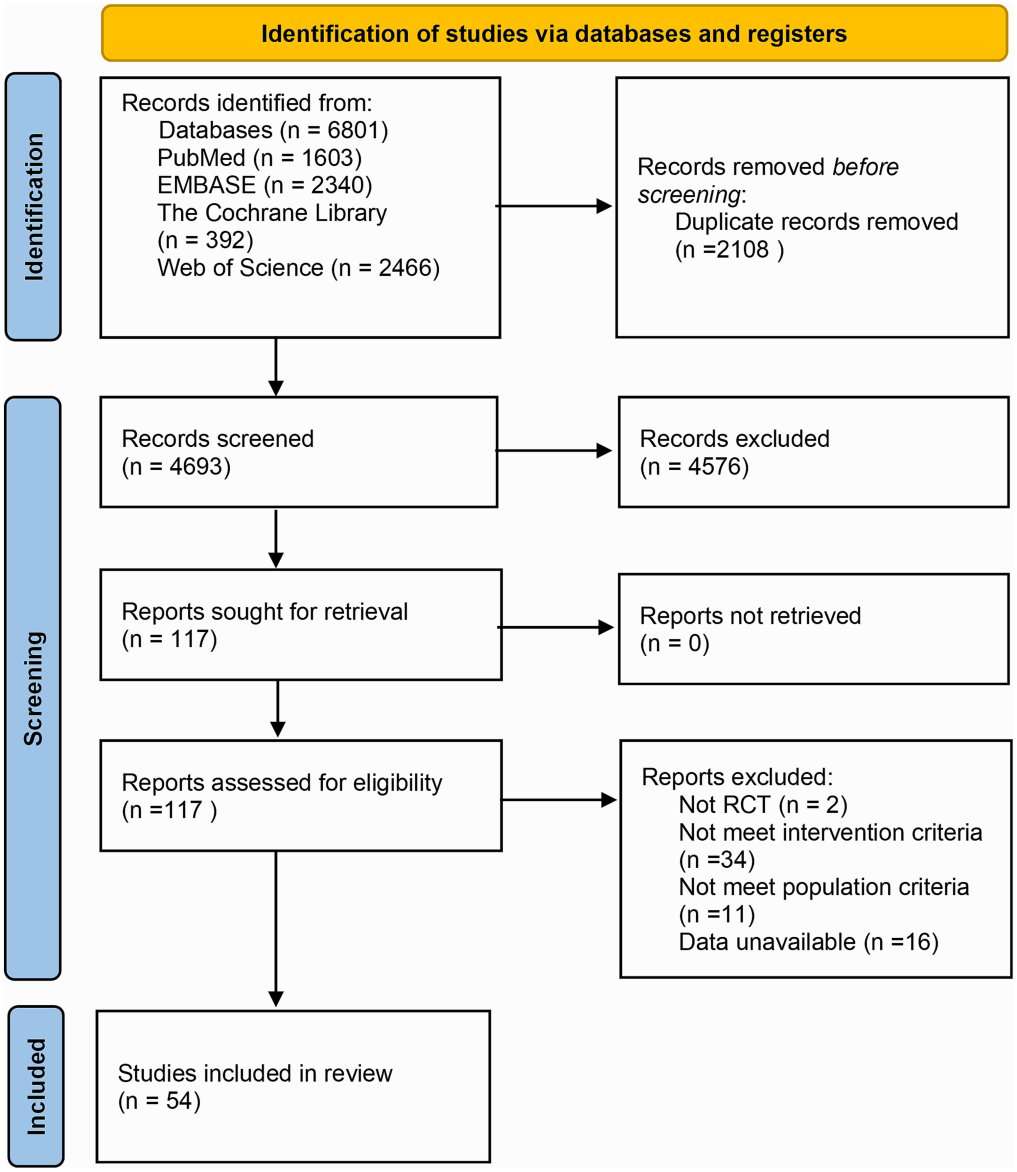
Literature screening flow chart.

In total, 6,801 articles were retrieved from the above databases. Endnote X9 was leveraged to search for study titles with terms such as “animal” “mouse” “cell” “review” “abstract” and “network pharmacology” to exclude non-RCTs. Through examining the abstracts, other studies that did not meet the inclusion criteria were excluded, leaving 117 articles. Upon reading the entire text, further screening was executed on the PICOS framework. Among the 117 articles, 2 were non-RCTs, 34 had interventions in the treatment or control groups that did not meet the inclusion criteria, 11 involved participants with NAFLD combined with DM or pediatric NAFLD, and 16 had no available outcome measures. Ultimately, 54 articles were included in the NMA, and the detailed screening procedure is depicted in Figure 1.

### 3.2 Basic characteristics and quality assessment of the included studies

The basic characteristics of the studies included are available in Supplementary material 2. In total, 54 RCTs were included (23–76), involving 3,132 participants aged 18 to 70 years. Males represented approximately 56.84%, and females accounted for approximately 45.56%. Forty-four studies were conducted in Iran, 3 in China, 2 in India, and 1 each in Australia, Denmark, Japan, Malaysia, and Pakistan. The type of intervention was dietary polyphenol supplements or diets containing certain dietary polyphenol active ingredients. Curcumin was employed in 18 studies, resveratrol in 8, anthocyanin in 5, silymarin in 6, catechin in 4, CA in 4, EA in 2, genistin/genistein in 2, naringenin in 2, DHM in 1, hesperidin in 1, quercetin in 1, and GA and CA in 1.

**Figure 2:**
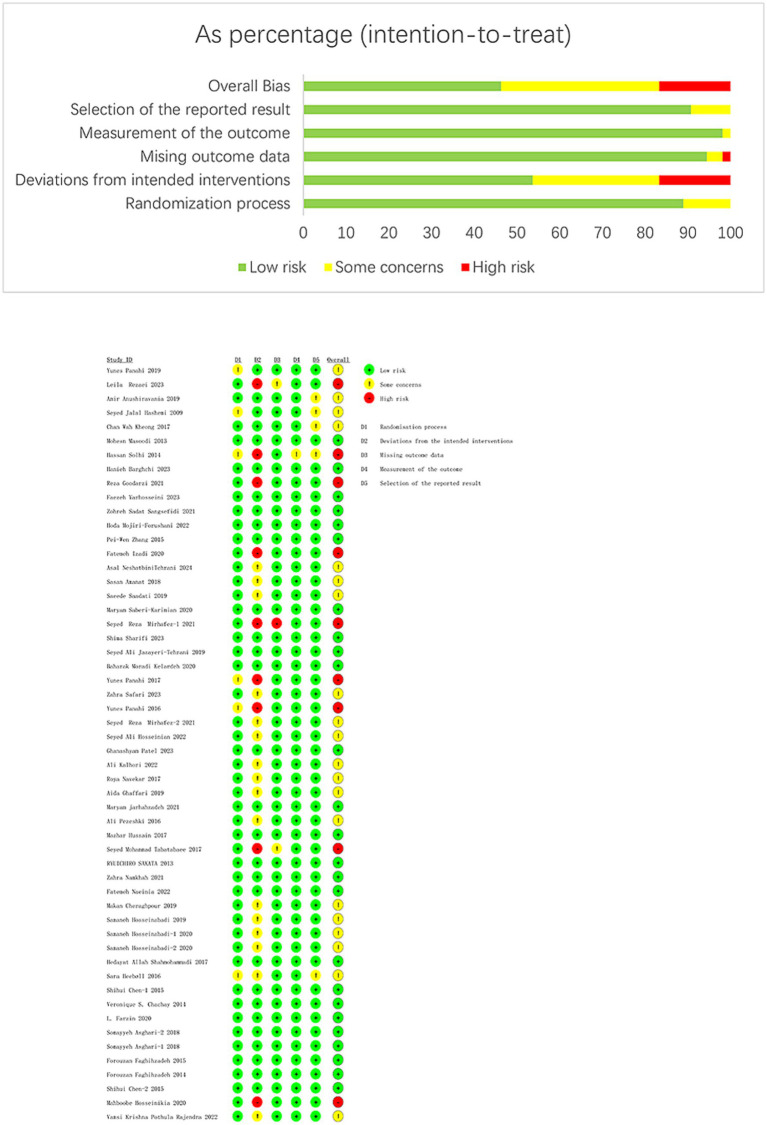
Quality evaluation of included studies.

The results of the quality evaluation are available in Figure 2. Out of the studies, 9 (16.66%) were high risk, 25 (46.30%) were low risk, and 20 (37.04%) had some risk. Among the nine high-risk studies, six had a dropout rate over 10% (27, 29, 50, 62, 67, 75), and three had a dropout rate over 10% and did not employ a double-blind approach (32, 33, 56).

### 3.3 NMA results

#### 3.3.1 Network diagram

**Figure 3:**
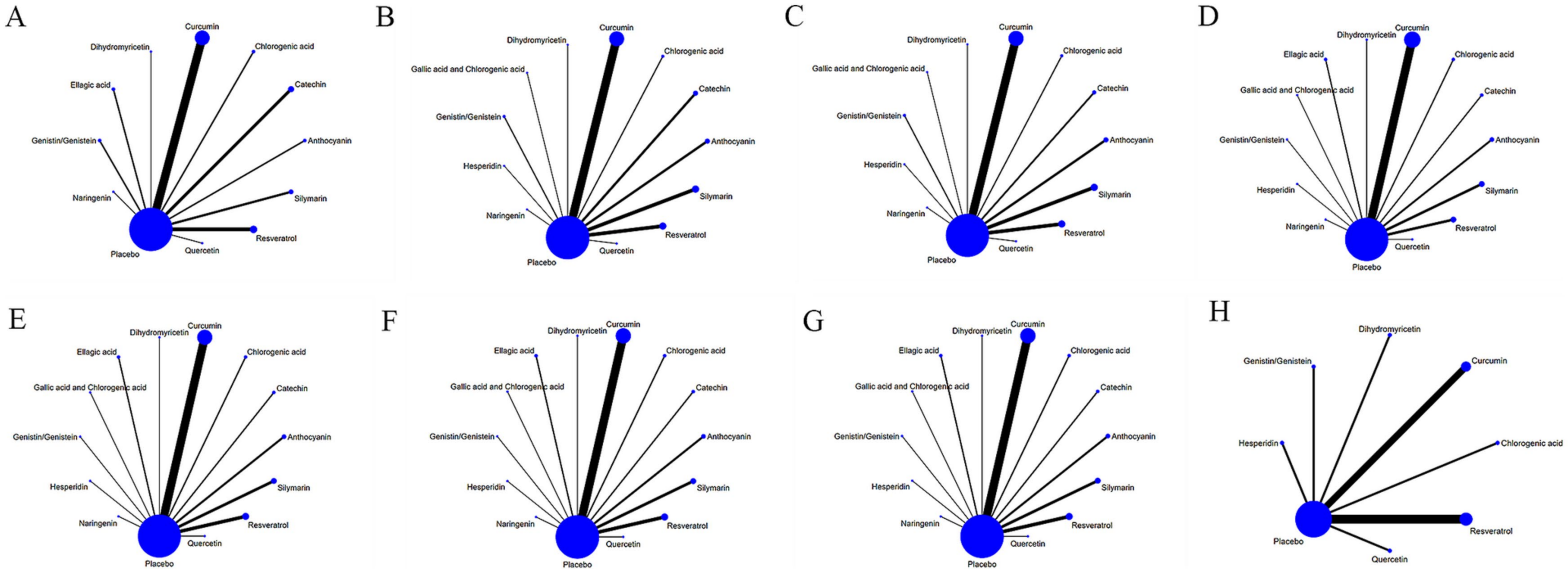
Network relationship diagram of different outcome indicators in NAFLD patients treated with different dietary polyphenol supplements. **(A)** BMI; **(B)** ALT; **(C)** AST; **(D)** TC; **(E)** TG; **(F)** HDL-C; **(G)** LDL-C; **(H)** TNF-α.

Figure 3 displays the network diagrams of outcome measures (such as BMI) in the treatment of NAFLD with various dietary polyphenols. Each node in the diagram represented a polyphenol or placebo, and the connecting line between two nodes represented a direct comparison of two polyphenols or any polyphenol with a placebo. The size of each node and the width of the connecting line were proportional to the number of studies (22). The network diagrams of other outcome measures can be found in Supplementary material 3.

#### 3.3.2 SUCRA plot

**Figure 4:**
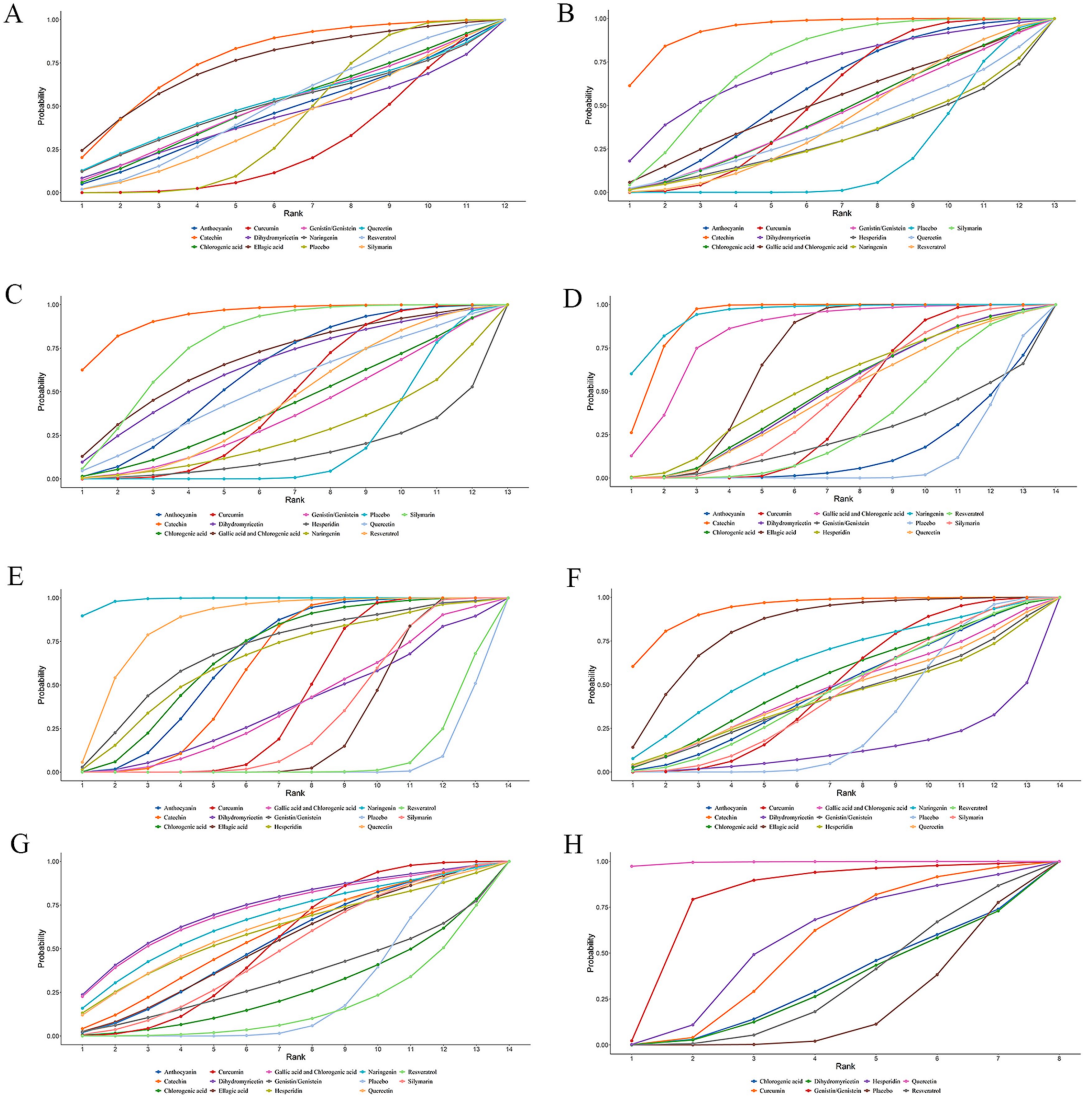
SUCRA diagram of changes in different outcome indicators caused by different dietary polyphenol supplements in the treatment of NAFLD. **(A)** BMI; **(B)** ALT; **(C)** AST; **(D)** TC; **(E)** TG; **(F)** HDL-C; **(G)** LDL-C; **(H)** TNF-α.

The SUCRA varied from 0 to 100%. A larger SUCRA indicated that the dietary polyphenol was ranked higher compared to other polyphenols and was more effective in treating NAFLD. Figure 4 displays the SUCRAs for outcome measures, including BMI, following the treatment of NAFLD with various dietary polyphenol supplements. The SUCRAs for other outcome measures are provided in Supplementary material 3.

#### 3.3.3 Anthropometric outcome measures

The anthropometric outcome measures included weight, BMI, WHR, WC, HC, SBP, and DBP, with 34 studies reporting BMI. The NMA results of weight, WC, HC, WHR, SBP, and DBP are available in Supplementary material 3.

The NMA results exhibited no marked differences in BMI changes induced by these dietary polyphenols (Table 1). According to the SUCRA probability ranking for BMI reduction, catechin (77.74%) was ranked highest, followed by EA (74.29%) and quercetin (51.58%) (Figure 4).

**Table 1:** League table of BMI of different dietary polyphenols.

Placebo	EA	Anthocyanin	Genistin/Genistein	CA	Curcumin	Naringenin	Catechin	Resveratrol	DHM	Silymarin	Quercetin
Placebo	−1.99 (−5.66, 1.66)	−0.16 (−3.96, 3.63)	−0.43 (−4.34, 3.45)	−0.44 (−4.05, 3.18)	0.67 (−0.89, 2.22)	−0.49 (−5.53, 4.56)	−2.05 (−4.76, 0.7)	−0.37 (−2.92, 2.15)	0.06 (−5.02, 5.1)	0.04 (−2.96, 3.04)	−0.56 (−5.57, 4.45)
1.99 (−1.66, 5.66)	EA	1.83 (−3.41, 7.11)	1.55 (−3.78, 6.9)	1.55 (−3.59, 6.71)	2.67 (−1.32, 6.64)	1.5 (−4.75, 7.74)	−0.05 (−4.59, 4.55)	1.61 (−2.83, 6.07)	2.05 (−4.2, 8.3)	2.04 (−2.69, 6.77)	1.43 (−4.76, 7.66)
0.16 (−3.63, 3.96)	−1.83 (−7.11, 3.41)	Anthocyanin	−0.28 (−5.72, 5.14)	−0.28 (−5.52, 4.97)	0.83 (−3.28, 4.94)	−0.33 (−6.66, 5.99)	−1.88 (−6.57, 2.8)	−0.22 (−4.77, 4.36)	0.21 (−6.12, 6.56)	0.2 (−4.66, 5.03)	−0.41 (−6.7, 5.89)
0.43 (−3.45, 4.34)	−1.55 (−6.9, 3.78)	0.28 (−5.14, 5.72)	Genistin/Genistein	−0.01 (−5.3, 5.32)	1.1 (−3.08, 5.3)	−0.06 (−6.41, 6.33)	−1.61 (−6.36, 3.14)	0.05 (−4.58, 4.7)	0.49 (−5.88, 6.87)	0.47 (−4.42, 5.41)	−0.13 (−6.46, 6.19)
0.44 (−3.18, 4.05)	−1.55 (−6.71, 3.59)	0.28 (−4.97, 5.52)	0.01 (−5.32, 5.3)	CA	1.12 (−2.83, 5.03)	−0.04 (−6.24, 6.16)	−1.6 (−6.14, 2.94)	0.06 (−4.36, 4.49)	0.5 (−5.74, 6.71)	0.49 (−4.22, 5.17)	−0.13 (−6.31, 6.04)
−0.67 (−2.22, 0.89)	−2.67 (−6.64, 1.32)	−0.83 (−4.94, 3.28)	−1.1 (−5.3, 3.08)	−1.12 (−5.03, 2.83)	Curcumin	−1.17 (−6.43, 4.11)	−2.72 (−5.83, 0.44)	−1.04 (−4.03, 1.91)	−0.62 (−5.9, 4.68)	−0.63 (−4.01, 2.74)	−1.23 (−6.48, 4.01)
0.49 (−4.56, 5.53)	−1.5 (−7.74, 4.75)	0.33 (−5.99, 6.66)	0.06 (−6.33, 6.41)	0.04 (−6.16, 6.24)	1.17 (−4.11, 6.43)	Naringenin	−1.56 (−7.29, 4.18)	0.11 (−5.52, 5.76)	0.55 (−6.57, 7.64)	0.54 (−5.36, 6.42)	−0.07 (−7.18, 7.05)
2.05 (−0.7, 4.76)	0.05 (−4.55, 4.59)	1.88 (−2.8, 6.57)	1.61 (−3.14, 6.36)	1.6 (−2.94, 6.14)	2.72 (−0.44, 5.83)	1.56 (−4.18, 7.29)	Catechin	1.66 (−2.07, 5.39)	2.11 (−3.67, 7.83)	2.09 (−1.98, 6.12)	1.48 (−4.23, 7.21)
0.37 (−2.15, 2.92)	−1.61 (−6.07, 2.83)	0.22 (−4.36, 4.77)	−0.05 (−4.7, 4.58)	−0.06 (−4.49, 4.36)	1.04 (−1.91, 4.03)	−0.11 (−5.76, 5.52)	−1.66 (−5.39, 2.07)	Resveratrol	0.44 (−5.26, 6.07)	0.42 (−3.52, 4.33)	−0.19 (−5.79, 5.43)
−0.06 (−5.1, 5.02)	−2.05 (−8.3, 4.2)	−0.21 (−6.56, 6.12)	−0.49 (−6.87, 5.88)	−0.5 (−6.71, 5.74)	0.62 (−4.68, 5.9)	−0.55 (−7.64, 6.57)	−2.11 (−7.83, 3.67)	−0.44 (−6.07, 5.26)	DHM	−0.02 (−5.92, 5.88)	−0.63 (−7.76, 6.52)
−0.04 (−3.04, 2.96)	−2.04 (−6.77, 2.69)	−0.2 (−5.03, 4.66)	−0.47 (−5.41, 4.42)	−0.49 (−5.17, 4.22)	0.63 (−2.74, 4.01)	−0.54 (−6.42, 5.36)	−2.09 (−6.12, 1.98)	−0.42 (−4.33, 3.52)	0.02 (−5.88, 5.92)	Silymarin	−0.61 (−6.45, 5.25)
0.56 (−4.45, 5.57)	−1.43 (−7.66, 4.76)	0.41 (−5.89, 6.7)	0.13 (−6.19, 6.46)	0.13 (−6.04, 6.31)	1.23 (−4.01, 6.48)	0.07 (−7.05, 7.18)	−1.48 (−7.21, 4.23)	0.19 (−5.43, 5.79)	0.63 (−6.52, 7.76)	0.61 (−5.25, 6.45)	Quercetin

The blue module represents different dietary polyphenols. The green module represents row-column comparisons of intervention effects of different dietary polyphenols. The orange module represents column-row comparisons of intervention effects of different dietary polyphenols.

#### 3.3.4 Liver function-related outcome measures

Liver function-related outcome measures included ALT, AST, ALP, and GGT. ALT was reported in 42 studies, and AST in 41 studies. The NMA results of ALP and GGT can be found in Supplementary material 3.

The NMA results revealed that catechin was more effective than placebo [WMD = 19.14, 95% confidence interval (CI) = (8.87, 29.32)], curcumin [WMD = 12.93, 95% CI = (1.51, 24.24)], and resveratrol [WMD = −15.39, 95% CI = (−29.17, −1.55)] in reducing ALT. Additional results with differences are provided in Table 2. The SUCRA probability ranking for reducing serum ALT revealed that catechin (94.21%) was the most effective, followed by silymarin (74.76%) and DHM (70.86%) (Figure 4).

**Table 2:** League table of ALT of different dietary polyphenols.

Placebo	Anthocyanin	Genistin/Genistein	CA	Curcumin	Catechin	Resveratrol	DHM	Naringenin	Silymarin	GA and CA	Hesperidin	Quercetin
Placebo	−7.44 (−16.44, 1.79)	−4.24 (−17.82, 9.39)	−4.46 (−17.12, 8.44)	**−6.21 (−11.18, −1.23)**	**−19.14 (−29.32, −8.87)**	−3.75 (−13.06, 5.6)	−11.73 (−28.77, 5.54)	−0.74 (−17.49, 16.05)	**−11.11 (−19.06, −3.43)**	−6.15 (−23.03, 10.67)	−0.35 (−18.39, 17.67)	−2.43 (−19.1, 14.24)
7.44 (−1.79, 16.44)	Anthocyanin	3.2 (−13.31, 19.53)	2.96 (−12.77, 18.72)	1.22 (−9.28, 11.51)	−11.72 (−25.46, 1.96)	3.7 (−9.44, 16.74)	−4.29 (−23.79, 15.14)	6.69 (−12.56, 25.68)	−3.69 (−15.91, 8.16)	1.28 (−18.05, 20.27)	7.09 (−13.3, 27.15)	4.98 (−14.11, 23.9)
4.24 (−9.39, 17.82)	−3.2 (−19.53, 13.31)	Genistin/Genistein	−0.25 (−18.78, 18.64)	−1.98 (−16.47, 12.47)	−14.87 (−31.91, 2.12)	0.44 (−16, 17.08)	−7.5 (−29.26, 14.51)	3.5 (−18.04, 25.09)	−6.9 (−22.72, 8.74)	−1.93 (−23.54, 19.82)	3.82 (−18.59, 26.56)	1.79 (−19.75, 23.47)
4.46 (−8.44, 17.12)	−2.96 (−18.72, 12.77)	0.25 (−18.64, 18.78)	CA	−1.74 (−15.6, 11.88)	−14.68 (−31.07, 1.63)	0.71 (−15.18, 16.48)	−7.24 (−28.81, 14.11)	3.72 (−17.52, 24.65)	−6.67 (−21.91, 8.1)	−1.7 (−23.18, 19.37)	4.12 (−18.13, 26.14)	2.02 (−19.3, 22.99)
**6.21 (1.23, 11.18)**	−1.22 (−11.51, 9.28)	1.98 (−12.47, 16.47)	1.74 (−11.88, 15.6)	Curcumin	**−12.93 (−24.24, −1.51)**	2.45 (−8.1, 13.01)	−5.51 (−23.28, 12.38)	5.47 (−11.95, 22.96)	−4.91 (−14.28, 4.22)	0.05 (−17.49, 17.58)	5.86 (−12.89, 24.53)	3.74 (−13.64, 21.17)
**19.14 (8.87, 29.32)**	11.72 (−1.96, 25.46)	14.87 (−2.12, 31.91)	14.68 (−1.63, 31.07)	**12.93 (1.51, 24.24)**	Catechin	**15.39 (1.55, 29.17)**	7.45 (−12.62, 27.28)	18.43 (−1.24, 38)	8.03 (−5.01, 20.72)	12.98 (−6.8, 32.65)	18.79 (−1.98, 39.4)	16.71 (−2.89, 36.18)
3.75 (−5.6, 13.06)	−3.7 (−16.74, 9.44)	−0.44 (−17.08, 16)	−0.71 (−16.48, 15.18)	−2.45 (−13.01, 8.1)	**−15.39 (−29.17, −1.55)**	Resveratrol	−7.97 (−27.44, 11.57)	2.99 (−16.25, 22.19)	−7.38 (−19.7, 4.69)	−2.41 (−21.81, 16.84)	3.41 (−16.96, 23.72)	1.29 (−17.83, 20.42)
11.73 (−5.54, 28.77)	4.29 (−15.14, 23.79)	7.5 (−14.51, 29.26)	7.24 (−14.11, 28.81)	5.51 (−12.38, 23.28)	−7.45 (−27.28, 12.62)	7.97 (−11.57, 27.44)	DHM	10.96 (−13.06, 34.91)	0.61 (−18.48, 19.24)	5.59 (−18.67, 29.51)	11.33 (−13.61, 36.33)	9.24 (−14.75, 33.15)
0.74 (−16.05, 17.49)	−6.69 (−25.68, 12.56)	−3.5 (−25.09, 18.04)	−3.72 (−24.65, 17.52)	−5.47 (−22.96, 11.95)	−18.43 (−38, 1.24)	−2.99 (−22.19, 16.25)	−10.96 (−34.91, 13.06)	Naringenin	−10.4 (−28.98, 7.99)	−5.44 (−29.27, 18.33)	0.37 (−24.24, 24.96)	−1.7 (−25.34, 21.9)
**11.11 (3.43, 19.06)**	3.69 (−8.16, 15.91)	6.9 (−8.74, 22.72)	6.67 (−8.1, 21.91)	4.91 (−4.22, 14.28)	−8.03 (−20.72, 5.01)	7.38 (−4.69, 19.7)	−0.61 (−19.24, 18.48)	10.4 (−7.99, 28.98)	Silymarin	4.95 (−13.56, 23.68)	10.77 (−8.76, 30.51)	8.68 (−9.59, 27.18)
6.15 (−10.67, 23.03)	−1.28 (−20.27, 18.05)	1.93 (−19.82, 23.54)	1.7 (−19.37, 23.18)	−0.05 (−17.58, 17.49)	−12.98 (−32.65, 6.8)	2.41 (−16.84, 21.81)	−5.59 (−29.51, 18.67)	5.44 (−18.33, 29.27)	−4.95 (−23.68, 13.56)	GA and CA	5.82 (−18.83, 30.5)	3.69 (−20.05, 27.62)
0.35 (−17.67, 18.39)	−7.09 (−27.15, 13.3)	−3.82 (−26.56, 18.59)	−4.12 (−26.14, 18.13)	−5.86 (−24.53, 12.89)	−18.79 (−39.4, 1.98)	−3.41 (−23.72, 16.96)	−11.33 (−36.33, 13.61)	−0.37 (−24.96, 24.24)	−10.77 (−30.51, 8.76)	−5.82 (−30.5, 18.83)	Hesperidin	−2.07 (−26.66, 22.44)
2.43 (−14.24, 19.1)	−4.98 (−23.9, 14.11)	−1.79 (−23.47, 19.75)	−2.02 (−22.99, 19.3)	−3.74 (−21.17, 13.64)	−16.71 (−36.18, 2.89)	−1.29 (−20.42, 17.83)	−9.24 (−33.15, 14.75)	1.7 (−21.9, 25.34)	−8.68 (−27.18, 9.59)	−3.69 (−27.62, 20.05)	2.07 (−22.44, 26.66)	Quercetin

^#Bold font represents a statistical difference. The blue module represents different dietary polyphenols. The green module represents row-column comparisons of intervention effects of different dietary polyphenols. The orange module represents column-row comparisons of intervention effects of different dietary polyphenols.^

Catechin demonstrated superior efficacy in lowering AST relative to placebo [WMD = 12.37, 95% CI = (4.68, 19.71)], curcumin [WMD = 9.32, 95% CI = (1.1, 17.2)], resveratrol [WMD = −9.5, 95% CI = (−18.62, −0.06)], naringenin [WMD = −12.77, 95% CI = (−24.56, −0.51)], and hesperidin [WMD = −15.23, 95% CI = (−27.67, −2.37)]. Additional results with differences are provided in Table 3. For lowering serum AST, the SUCRA probability ranking showed catechin (93.56%) as the highest, followed by silymarin (78.37%) and GA and CA (68.49%) (Figure 4).

**Table 3:** League table of AST of different dietary polyphenols.

Placebo	Anthocyanin	Genistin/Genistein	CA	Curcumin	Catechin	Resveratrol	DHM	Naringenin	Silymarin	GA and CA	Hesperidin	Quercetin
Placebo	−5.02 (−10.33, 0.16)	−1.78 (−9.32, 5.87)	−2.4 (−10.86, 6.04)	**−3.05 (−5.88, −0.24)**	**−12.37 (−19.71, −4.68)**	−2.87 (−8.29, 2.59)	−6.05 (−15.79, 3.71)	0.36 (−9.04, 9.77)	**−7.7 (−12.26, −2.98)**	−6.82 (−16.71, 3.18)	2.87 (−7.24, 12.93)	−4.05 (−13.72, 5.63)
5.02 (−0.16, 10.33)	Anthocyanin	3.25 (−5.89, 12.56)	2.62 (−7.34, 12.61)	1.96 (−3.93, 7.98)	−7.35 (−16.33, 2.09)	2.16 (−5.35, 9.81)	−1.02 (−12.04, 10.06)	5.38 (−5.32, 16.2)	−2.68 (−9.53, 4.44)	−1.79 (−12.94, 9.51)	7.88 (−3.46, 19.31)	0.96 (−9.96, 12.08)
1.78 (−5.87, 9.32)	−3.25 (−12.56, 5.89)	Genistin/Genistein	−0.61 (−12.02, 10.63)	−1.28 (−9.48, 6.76)	−10.6 (−21.17, 0.19)	−1.08 (−10.4, 8.29)	−4.26 (−16.7, 8.07)	2.15 (−10.04, 14.15)	−5.91 (−14.75, 3.05)	−5.03 (−17.55, 7.44)	4.64 (−8.06, 17.2)	−2.28 (−14.67, 10)
2.4 (−6.04, 10.86)	−2.62 (−12.61, 7.34)	0.61 (−10.63, 12.02)	CA	−0.66 (−9.53, 8.23)	−9.97 (−21.11, 1.58)	−0.47 (−10.47, 9.65)	−3.63 (−16.54, 9.35)	2.76 (−9.83, 15.5)	−5.28 (−14.83, 4.46)	−4.41 (−17.39, 8.71)	5.26 (−7.82, 18.49)	−1.65 (−14.43, 11.21)
**3.05 (0.24, 5.88)**	−1.96 (−7.98, 3.93)	1.28 (−6.76, 9.48)	0.66 (−8.23, 9.53)	Curcumin	**−9.32 (−17.2, −1.1)**	0.19 (−5.92, 6.34)	−2.99 (−13.14, 7.2)	3.43 (−6.39, 13.24)	−4.64 (−9.98, 0.88)	−3.77 (−14.05, 6.62)	5.91 (−4.56, 16.41)	−1 (−11.08, 9.06)
**12.37 (4.68, 19.71)**	7.35 (−2.09, 16.33)	10.6 (−0.19, 21.17)	9.97 (−1.58, 21.11)	**9.32 (1.1, 17.2)**	Catechin	**9.5 (0.06, 18.62)**	6.35 (−6.23, 18.42)	**12.77 (0.51, 24.56)**	4.68 (−4.22, 13.35)	5.57 (−7.07, 17.87)	**15.23 (2.37, 27.67)**	8.32 (−4.18, 20.33)
2.87 (−2.59, 8.29)	−2.16 (−9.81, 5.35)	1.08 (−8.29, 10.4)	0.47 (−9.65, 10.47)	−0.19 (−6.34, 5.92)	**−9.5 (−18.62, −0.06)**	Resveratrol	−3.18 (−14.45, 7.99)	3.24 (−7.66, 14.01)	−4.83 (−11.92, 2.36)	−3.96 (−15.28, 7.33)	5.74 (−5.78, 17.13)	−1.19 (−12.35, 9.87)
6.05 (−3.71, 15.79)	1.02 (−10.06, 12.04)	4.26 (−8.07, 16.7)	3.63 (−9.35, 16.54)	2.99 (−7.2, 13.14)	−6.35 (−18.42, 6.23)	3.18 (−7.99, 14.45)	DHM	6.4 (−7.13, 19.94)	−1.65 (−12.38, 9.18)	−0.76 (−14.66, 13.18)	8.89 (−5.22, 22.96)	1.99 (−11.71, 15.74)
−0.36 (−9.77, 9.04)	−5.38 (−16.2, 5.32)	−2.15 (−14.15, 10.04)	−2.76 (−15.5, 9.83)	−3.43 (−13.24, 6.39)	**−12.77 (−24.56, −0.51)**	−3.24 (−14.01, 7.66)	−6.4 (−19.94, 7.13)	Naringenin	−8.08 (−18.47, 2.51)	−7.16 (−20.83, 6.53)	2.47 (−11.26, 16.25)	−4.4 (−17.92, 9.1)
**7.7 (2.98, 12.26)**	2.68 (−4.44, 9.53)	5.91 (−3.05, 14.75)	5.28 (−4.46, 14.83)	4.64 (−0.88, 9.98)	−4.68 (−13.35, 4.22)	4.83 (−2.36, 11.92)	1.65 (−9.18, 12.38)	8.08 (−2.51, 18.47)	Silymarin	0.88 (−10.1, 11.8)	10.56 (−0.61, 21.57)	3.64 (−7.16, 14.26)
6.82 (−3.18, 16.71)	1.79 (−9.51, 12.94)	5.03 (−7.44, 17.55)	4.41 (−8.71, 17.39)	3.77 (−6.62, 14.05)	−5.57 (−17.87, 7.07)	3.96 (−7.33, 15.28)	0.76 (−13.18, 14.66)	7.16 (−6.53, 20.83)	−0.88 (−11.8, 10.1)	GA and CA	9.69 (−4.56, 23.78)	2.76 (−11.06, 16.63)
−2.87 (−12.93, 7.24)	−7.88 (−19.31, 3.46)	−4.64 (−17.2, 8.06)	−5.26 (−18.49, 7.82)	−5.91 (−16.41, 4.56)	**−15.23 (−27.67, −2.37)**	−5.74 (−17.13, 5.78)	−8.89 (−22.96, 5.22)	−2.47 (−16.25, 11.26)	−10.56 (−21.57, 0.61)	−9.69 (−23.78, 4.56)	Hesperidin	−6.93 (−20.86, 7.1)
4.05 (−5.63, 13.72)	−0.96 (−12.08, 9.96)	2.28 (−10, 14.67)	1.65 (−11.21, 14.43)	1 (−9.06, 11.08)	−8.32 (−20.33, 4.18)	1.19 (−9.87, 12.35)	−1.99 (−15.74, 11.71)	4.4 (−9.1, 17.92)	−3.64 (−14.26, 7.16)	−2.76 (−16.63, 11.06)	6.93 (−7.1, 20.86)	Quercetin

^#Bold font represents a statistical difference. The blue module represents different dietary polyphenols. The green module represents row-column comparisons of intervention effects of different dietary polyphenols. The orange module represents column-row comparisons of intervention effects of different dietary polyphenols.^

#### 3.3.5 Blood lipid-related outcome measures

Blood lipid-related outcome measures involved TC, TG, HDL-C, and LDL-C. Thirty-five studies reported TC, TG, HDL-C, and LDL-C each.

According to the NMA results, naringenin outperformed placebo [WMD = 32.84, 95% CI = (14.31, 51.46)], EA [WMD = 19.11, 95% CI = (0.56, 37.74)], anthocyanin [WMD = 32.65, 95% CI = (11.93, 53.47)], genistin/genistein [WMD = 31.34, 95% CI = (5.24, 57.52)], CA [WMD = 23.33, 95% CI = (0.84, 45.79)], curcumin [WMD = 25.26, 95% CI = (6.52, 44.09)], resveratrol [WMD = −27.86, 95% CI = (−48.1, −7.7)], DHM [WMD = −23.55, 95% CI = (−45.81, −1.34)], silymarin [WMD = −24.29, 95% CI = (−44.68, −3.99)], and quercetin [WMD = −24.13, 95% CI = (−47.01, −1.39)] in reducing TC. Catechin exhibited superior efficacy in lowering TC relative to placebo [WMD = 28.44, 95% CI = (19.52, 37.29)], EA [WMD = 14.71, 95% CI = (5.78, 23.58)], anthocyanin [WMD = 28.29, 95% CI = (15.41, 41.13)], genistin/genistein [WMD = 26.99, 95% CI = (6.53, 47.44)], CA [WMD = 18.94, 95% CI = (3.51, 34.39)], curcumin [WMD = 20.86, 95% CI = (11.56, 30.12)], resveratrol [WMD = −23.46, 95% CI = (−35.41, −11.52)], DHM [WMD = −19.12, 95% CI = (−34.18, −4.02)], silymarin [WMD = −19.93, 95% CI = (−31.95, −7.79)], and quercetin [WMD = −19.76, 95% CI = (−35.56, −3.88)]. More results with differences are presented in Table 4. The SUCRA probability ranking for lowering serum TC demonstrated that naringenin (94.59%) was the most effective, followed by catechin (92.26%) and GA and CA (83.52%) (Figure 4).

**Table 4:** League table of TC of different dietary polyphenols.

Placebo	EA	Anthocyanin	Genistin/Genistein	CA	Curcumin	Naringenin	Catechin	Resveratrol	DHM	Silymarin	GA and CA	Hesperidin	Quercetin
Placebo	**−13.73 (−14.12, −13.34)**	−0.15 (−9.51, 9.19)	−1.45 (−19.94, 16.93)	−9.48 (−22.21, 3.12)	**−7.58 (−10.28, −4.89)**	**−32.84 (−51.46, −14.31)**	**−28.44 (−37.29, −19.52)**	−4.99 (−12.97, 3.01)	−9.31 (−21.54, 2.91)	**−8.51 (−16.74, −0.34)**	**−23.31 (−38.98, −7.65)**	−10.79 (−26.41, 4.76)	−8.71 (−21.91, 4.52)
**13.73 (13.34, 14.12)**	EA	**13.58 (4.21, 22.93)**	12.28 (−6.2, 30.67)	4.25 (−8.47, 16.87)	**6.15 (3.43, 8.87)**	**−19.11 (−37.74, −0.56)**	**−14.71 (−23.58, −5.78)**	**8.75 (0.76, 16.77)**	4.42 (−7.8, 16.64)	5.22 (−3.02, 13.4)	−9.58 (−25.26, 6.09)	2.95 (−12.66, 18.51)	5.03 (−8.18, 18.25)
0.15 (−9.19, 9.51)	**−13.58 (−22.93, −4.21)**	Anthocyanin	−1.31 (−21.9, 19.29)	−9.32 (−25.01, 6.33)	−7.44 (−17.14, 2.31)	**−32.65 (−53.47, −11.93)**	**−28.29 (−41.13, −15.41)**	−4.82 (−17.11, 7.51)	−9.16 (−24.45, 6.28)	−8.39 (−20.75, 4.09)	**−23.15 (−41.33, −4.88)**	−10.63 (−28.75, 7.45)	−8.53 (−24.65, 7.66)
1.45 (−16.93, 19.94)	−12.28 (−30.67, 6.2)	1.31 (−19.29, 21.9)	Genistin/Genistein	−8.04 (−30.42, 14.33)	−6.13 (−24.7, 12.56)	**−31.34 (−57.52, −5.24)**	**−26.99 (−47.44, −6.53)**	−3.5 (−23.61, 16.56)	−7.82 (−29.91, 14.32)	−7.08 (−27.22, 13)	−21.81 (−45.98, 2.31)	−9.33 (−33.38, 14.79)	−7.25 (−29.84, 15.42)
9.48 (−3.12, 22.21)	−4.25 (−16.87, 8.47)	9.32 (−6.33, 25.01)	8.04 (−14.33, 30.42)	CA	1.92 (−10.99, 14.91)	**−23.33 (−45.79, −0.84)**	**−18.94 (−34.39, −3.51)**	4.5 (−10.37, 19.44)	0.2 (−17.4, 17.78)	0.98 (−14.09, 16.09)	−13.8 (−33.9, 6.32)	−1.29 (−21.42, 18.76)	0.81 (−17.41, 19.02)
**7.58 (4.89, 10.28)**	**−6.15 (−8.87, −3.43)**	7.44 (−2.31, 17.14)	6.13 (−12.56, 24.7)	−1.92 (−14.91, 10.99)	Curcumin	**−25.26 (−44.09, −6.52)**	**−20.86 (−30.12, −11.56)**	2.59 (−5.82, 11.02)	−1.72 (−14.25, 10.81)	−0.94 (−9.6, 7.68)	−15.73 (−31.61, 0.16)	−3.19 (−19.04, 12.56)	−1.12 (−14.58, 12.34)
**32.84 (14.31, 51.46)**	**19.11 (0.56, 37.74)**	**32.65 (11.93, 53.47)**	**31.34 (5.24, 57.52)**	**23.33 (0.84, 45.79)**	**25.26 (6.52, 44.09)**	Naringenin	4.37 (−16.15, 25.05)	**27.86 (7.7, 48.1)**	**23.55 (1.34, 45.81)**	**24.29 (3.99, 44.68)**	9.53 (−14.75, 33.79)	22.04 (−2.35, 46.13)	**24.13 (1.39, 47.01)**
**28.44 (19.52, 37.29)**	**14.71 (5.78, 23.58)**	**28.29 (15.41, 41.13)**	**26.99 (6.53, 47.44)**	**18.94 (3.51, 34.39)**	**20.86 (11.56, 30.12)**	−4.37 (−25.05, 16.15)	Catechin	**23.46 (11.52, 35.41)**	**19.12 (4.02, 34.18)**	**19.93 (7.79, 31.95)**	5.14 (−12.89, 23.11)	17.64 (−0.33, 35.56)	**19.76 (3.88, 35.56)**
4.99 (−3.01, 12.97)	**−8.75 (−16.77, −0.76)**	4.82 (−7.51, 17.11)	3.5 (−16.56, 23.61)	−4.5 (−19.44, 10.37)	−2.59 (−11.02, 5.82)	**−27.86 (−48.1, −7.7)**	**−23.46 (−35.41, −11.52)**	Resveratrol	−4.33 (−18.9, 10.2)	−3.53 (−14.98, 7.88)	**−18.3 (−35.99, −0.7)**	−5.79 (−23.29, 11.59)	−3.73 (−19.09, 11.75)
9.31 (−2.91, 21.54)	−4.42 (−16.64, 7.8)	9.16 (−6.28, 24.45)	7.82 (−14.32, 29.91)	−0.2 (−17.78, 17.4)	1.72 (−10.81, 14.25)	**−23.55 (−45.81, −1.34)**	**−19.12 (−34.18, −4.02)**	4.33 (−10.2, 18.9)	DHM	0.79 (−13.94, 15.37)	−13.99 (−33.9, 5.98)	−1.49 (−21.36, 18.28)	0.6 (−17.34, 18.49)
**8.51 (0.34, 16.74)**	−5.22 (−13.4, 3.02)	8.39 (−4.09, 20.75)	7.08 (−13, 27.22)	−0.98 (−16.09, 14.09)	0.94 (−7.68, 9.6)	**−24.29 (−44.68, −3.99)**	**−19.93 (−31.95, −7.79)**	3.53 (−7.88, 14.98)	−0.79 (−15.37, 13.94)	Silymarin	−14.8 (−32.41, 3.02)	−2.27 (−19.93, 15.32)	−0.19 (−15.66, 15.35)
**23.31 (7.65, 38.98)**	9.58 (−6.09, 25.26)	**23.15 (4.88, 41.33)**	21.81 (−2.31, 45.98)	13.8 (−6.32, 33.9)	15.73 (−0.16, 31.61)	−9.53 (−33.79, 14.75)	−5.14 (−23.11, 12.89)	**18.3 (0.7, 35.99)**	13.99 (−5.98, 33.9)	14.8 (−3.02, 32.41)	GA and CA	12.5 (−9.6, 34.64)	14.61 (−5.83, 35.01)
10.79 (−4.76, 26.41)	−2.95 (−18.51, 12.66)	10.63 (−7.45, 28.75)	9.33 (−14.79, 33.38)	1.29 (−18.76, 21.42)	3.19 (−12.56, 19.04)	−22.04 (−46.13, 2.35)	−17.64 (−35.56, 0.33)	5.79 (−11.59, 23.29)	1.49 (−18.28, 21.36)	2.27 (−15.32, 19.93)	−12.5 (−34.64, 9.6)	Hesperidin	2.11 (−18.32, 22.52)
8.71 (−4.52, 21.91)	−5.03 (−18.25, 8.18)	8.53 (−7.66, 24.65)	7.25 (−15.42, 29.84)	−0.81 (−19.02, 17.41)	1.12 (−12.34, 14.58)	**−24.13 (−47.01, −1.39)**	**−19.76 (−35.56, −3.88)**	3.73 (−11.75, 19.09)	−0.6 (−18.49, 17.34)	0.19 (−15.35, 15.66)	−14.61 (−35.01, 5.83)	−2.11 (−22.52, 18.32)	Quercetin

^#Bold font represents a statistical difference. The blue module represents different dietary polyphenols. The green module represents row-column comparisons of intervention effects of different dietary polyphenols. The orange module represents column-row comparisons of intervention effects of different dietary polyphenols.^

Naringenin outperformed placebo [WMD = 92.5, 95% CI = (58.09, 126.53)], EA [WMD = 76.18, 95% CI = (41.77, 110.18)], anthocyanin [WMD = 57.46, 95% CI = (19.56, 94.86)], CA [WMD = 54.89, 95% CI = (13.59, 95.86)], curcumin [WMD = 70, 95% CI = (35.33, 104.33)], catechin [WMD = −61.26, 95% CI = (−96.53, −25.7)], resveratrol [WMD = −90.54, 95% CI = (−126.58, −54.25)], DHM [WMD = −72.82, 95% CI = (−120.49, −25.32)], silymarin [WMD = −74.94, 95% CI = (−110.26, −39.23)], GA and CA [WMD = −72.12, 95% CI = (−114.89, −28.87)], and hesperidin [WMD = −53.78, 95% CI = (−105.8, −1.89)] in lowering TG. More results with differences are presented in Table 5. In terms of lowering serum TG, the SUCRA probability ranking results showed that naringenin (99.00%) was ranked highest, followed by quercetin (85.73%) and genistin/genistein (69.22%) (Figure 4).

**Table 5:** League table of TG of different dietary polyphenols.

Placebo	EA	Anthocyanin	Genistin/Genistein	CA	Curcumin	Naringenin	Catechin	Resveratrol	DHM	Silymarin	GA and CA	Hesperidin	Quercetin
Placebo	**−16.32 (−16.94, −15.7)**	**−35.05 (−50.65, −19.59)**	**−43.06 (−84.4, −1.69)**	**−37.57 (−60.56, −14.67)**	**−22.5 (−26.99, −17.99)**	**−92.5 (−126.53, −58.09)**	**−31.26 (−40.11, −22.42)**	−1.93 (−13.76, 9.77)	−19.65 (−52.57, 13.67)	**−17.57 (−27.27, −7.85)**	−20.42 (−46.66, 5.68)	−38.59 (−77.84, 0.57)	**−57.49 (−87.27, −27.45)**
**16.32 (15.7, 16.94)**	EA	**−18.73 (−34.32, −3.25)**	−26.75 (−68.07, 14.67)	−21.25 (−44.25, 1.68)	**−6.18 (−10.71, −1.63)**	**−76.18 (−110.18, −41.77)**	**−14.93 (−23.79, −6.07)**	**14.39 (2.55, 26.11)**	−3.34 (−36.27, 30)	−1.25 (−10.97, 8.46)	−4.11 (−30.37, 22.01)	−22.28 (−61.51, 16.91)	**−41.17 (−70.97, −11.15)**
**35.05 (19.59, 50.65)**	**18.73 (3.25, 34.32)**	Anthocyanin	−8.04 (−52.19, 36)	−2.5 (−30.32, 25.16)	12.54 (−3.59, 28.82)	**−57.46 (−94.86, −19.56)**	3.79 (−13.98, 21.7)	**33.07 (13.65, 52.58)**	15.43 (−21.06, 52.1)	17.46 (−0.84, 35.86)	14.61 (−15.89, 45.04)	−3.59 (−45.66, 38.67)	−22.43 (−56.02, 11.36)
**43.06 (1.69, 84.4)**	26.75 (−14.67, 68.07)	8.04 (−36, 52.19)	Genistin/Genistein	5.51 (−41.91, 52.82)	20.56 (−21.14, 62.11)	−49.42 (−103.34, 4.18)	11.79 (−30.56, 54.22)	41.08 (−1.86, 84.2)	23.53 (−29.36, 76.41)	25.49 (−16.92, 67.92)	22.69 (−26.42, 71.48)	4.42 (−52.48, 61.47)	−14.4 (−65.44, 36.76)
**37.57 (14.67, 60.56)**	21.25 (−1.68, 44.25)	2.5 (−25.16, 30.32)	−5.51 (−52.82, 41.91)	CA	15.07 (−8.19, 38.43)	**−54.89 (−95.86, −13.59)**	6.34 (−18.22, 30.97)	**35.67 (10.01, 61.34)**	17.97 (−22.18, 58.24)	20.02 (−4.77, 44.87)	17.15 (−17.5, 51.9)	−1.02 (−46.43, 44.49)	−19.84 (−57.54, 17.91)
**22.5 (17.99, 26.99)**	**6.18 (1.63, 10.71)**	−12.54 (−28.82, 3.59)	−20.56 (−62.11, 21.14)	−15.07 (−38.43, 8.19)	Curcumin	**−70 (−104.33, −35.33)**	−8.74 (−18.66, 1.19)	**20.56 (7.92, 33.11)**	2.82 (−30.36, 36.41)	4.94 (−5.82, 15.64)	2.05 (−24.53, 28.56)	−16.12 (−55.57, 23.28)	**−34.94 (−65.11, −4.65)**
**92.5 (58.09, 126.53)**	**76.18 (41.77, 110.18)**	**57.46 (19.56, 94.86)**	49.42 (−4.18, 103.34)	**54.89 (13.59, 95.86)**	**70 (35.33, 104.33)**	Naringenin	**61.26 (25.7, 96.53)**	**90.54 (54.25, 126.58)**	**72.82 (25.32, 120.49)**	**74.94 (39.23, 110.26)**	**72.12 (28.87, 114.89)**	**53.78 (1.89, 105.8)**	35.02 (−10.33, 80.58)
**31.26 (22.42, 40.11)**	**14.93 (6.07, 23.79)**	−3.79 (−21.7, 13.98)	−11.79 (−54.22, 30.56)	−6.34 (−30.97, 18.22)	8.74 (−1.19, 18.66)	**−61.26 (−96.53, −25.7)**	Catechin	**29.31 (14.56, 44.06)**	11.61 (−22.44, 46.1)	**13.68 (0.54, 26.8)**	10.79 (−16.82, 38.46)	−7.37 (−47.56, 32.73)	−26.24 (−57.29, 5.11)
1.93 (−9.77, 13.76)	**−14.39 (−26.11, −2.55)**	**−33.07 (−52.58, −13.65)**	−41.08 (−84.2, 1.86)	**−35.67 (−61.34, −10.01)**	**−20.56 (−33.11, −7.92)**	**−90.54 (−126.58, −54.25)**	**−29.31 (−44.06, −14.56)**	Resveratrol	−17.75 (−52.68, 17.65)	**−15.64 (−30.81, −0.37)**	−18.5 (−47.03, 10.16)	−36.65 (−77.57, 4.34)	**−55.5 (−87.62, −23.25)**
19.65 (−13.67, 52.57)	3.34 (−30, 36.27)	−15.43 (−52.1, 21.06)	−23.53 (−76.41, 29.36)	−17.97 (−58.24, 22.18)	−2.82 (−36.41, 30.36)	**−72.82 (−120.49, −25.32)**	−11.61 (−46.1, 22.44)	17.75 (−17.65, 52.68)	DHM	2.04 (−32.58, 36.49)	−0.77 (−43.23, 41.39)	−19 (−70.18, 32.3)	−37.85 (−82.31, 6.87)
**17.57 (7.85, 27.27)**	1.25 (−8.46, 10.97)	−17.46 (−35.86, 0.84)	−25.49 (−67.92, 16.92)	−20.02 (−44.87, 4.77)	−4.94 (−15.64, 5.82)	**−74.94 (−110.26, −39.23)**	**−13.68 (−26.8, −0.54)**	**15.64 (0.37, 30.81)**	−2.04 (−36.49, 32.58)	Silymarin	−2.87 (−30.73, 25.05)	−21.01 (−61.41, 19.35)	**−39.89 (−71.18, −8.4)**
20.42 (−5.68, 46.66)	4.11 (−22.01, 30.37)	−14.61 (−45.04, 15.89)	−22.69 (−71.48, 26.42)	−17.15 (−51.9, 17.5)	−2.05 (−28.56, 24.53)	**−72.12 (−114.89, −28.87)**	−10.79 (−38.46, 16.82)	18.5 (−10.16, 47.03)	0.77 (−41.39, 43.23)	2.87 (−25.05, 30.73)	GA and CA	−18.2 (−65.36, 29.19)	−36.95 (−76.51, 2.81)
38.59 (−0.57, 77.84)	22.28 (−16.91, 61.51)	3.59 (−38.67, 45.66)	−4.42 (−61.47, 52.48)	1.02 (−44.49, 46.43)	16.12 (−23.28, 55.57)	**−53.78 (−105.8, −1.89)**	7.37 (−32.73, 47.56)	36.65 (−4.34, 77.57)	19 (−32.3, 70.18)	21.01 (−19.35, 61.41)	18.2 (−29.19, 65.36)	Hesperidin	−18.83 (−68.08, 30.64)
**57.49 (27.45, 87.27)**	**41.17 (11.15, 70.97)**	22.43 (−11.36, 56.02)	14.4 (−36.76, 65.44)	19.84 (−17.91, 57.54)	**34.94 (4.65, 65.11)**	−35.02 (−80.58, 10.33)	26.24 (−5.11, 57.29)	**55.5 (23.25, 87.62)**	37.85 (−6.87, 82.31)	**39.89 (8.4, 71.18)**	36.95 (−2.81, 76.51)	18.83 (−30.64, 68.08)	Quercetin

^#Bold font represents a statistical difference. The blue module represents different dietary polyphenols. The green module represents row-column comparisons of intervention effects of different dietary polyphenols. The orange module represents column-row comparisons of intervention effects of different dietary polyphenols.^

Catechin was more effective in increasing HDL-C than placebo [WMD = −7.88, 95% CI = (−13.03, −2.48)], curcumin [WMD = −6.62, 95% CI = (−12.26, −0.82)], DHM [WMD = 11.36, 95% CI = (2.13, 20.36)], and silymarin [WMD = 6.93, 95% CI = (0.48, 13.09)]. Additional results with differences are presented in Table 6. According to the SUCRA probability ranking for increasing serum HDL-C, catechin (93.72%) was ranked highest, followed by EA (82.69%) and naringenin (63.05%) (Figure 4).

**Table 6:** League table of HDL-C of different dietary polyphenols.

Placebo	EA	Anthocyanin	Genistin/Genistein	CA	Curcumin	Naringenin	Catechin	Resveratrol	DHM	Silymarin Silymarin	GA and CA	Hesperidin	Quercetin
Placebo	**5.29 (0.69, 9.92)**	1.22 (−3.67, 6.33)	0.64 (−7.06, 8.38)	1.85 (−4.1, 7.81)	1.26 (−1.03, 3.43)	3.08 (−3.85, 10.03)	**7.88 (2.48, 13.03)**	1.13 (−3.38, 5.69)	−3.47 (−10.97, 3.99)	0.96 (−2.54, 4.46)	1.24 (−5.7, 8.23)	0.55 (−8, 9.1)	1.03 (−6.46, 8.53)
**−5.29 (−9.92, −0.69)**	EA	−4.08 (−10.77, 2.85)	−4.65 (−13.62, 4.35)	−3.46 (−10.95, 4.07)	−4.03 (−9.23, 1.01)	−2.2 (−10.54, 6.11)	2.6 (−4.54, 9.44)	−4.16 (−10.59, 2.34)	**−8.77 (−17.57, −0.02)**	−4.33 (−10.13, 1.48)	−4.06 (−12.4, 4.34)	−4.74 (−14.46, 4.97)	−4.27 (−13.04, 4.53)
−1.22 (−6.33, 3.67)	4.08 (−2.85, 10.77)	Anthocyanin	−0.58 (−9.87, 8.53)	0.61 (−7.24, 8.32)	0.04 (−5.61, 5.36)	1.86 (−6.77, 10.27)	6.65 (−0.81, 13.73)	−0.09 (−6.89, 6.6)	−4.7 (−13.8, 4.2)	−0.26 (−6.49, 5.73)	0.02 (−8.7, 8.49)	−0.67 (−10.63, 9.14)	−0.18 (−9.3, 8.72)
−0.64 (−8.38, 7.06)	4.65 (−4.35, 13.62)	0.58 (−8.53, 9.87)	Genistin/Genistein	1.21 (−8.64, 10.99)	0.62 (−7.5, 8.58)	2.43 (−7.92, 12.8)	7.23 (−2.23, 16.45)	0.49 (−8.41, 9.43)	−4.13 (−14.91, 6.62)	0.32 (−8.17, 8.79)	0.61 (−9.8, 11.02)	−0.09 (−11.6, 11.43)	0.39 (−10.41, 11.15)
−1.85 (−7.81, 4.1)	3.46 (−4.07, 10.95)	−0.61 (−8.32, 7.24)	−1.21 (−10.99, 8.64)	CA	−0.59 (−6.99, 5.68)	1.24 (−7.93, 10.36)	6.04 (−2.04, 13.86)	−0.71 (−8.19, 6.81)	−5.32 (−14.87, 4.26)	−0.87 (−7.83, 6)	−0.58 (−9.81, 8.56)	−1.28 (−11.72, 9.08)	−0.82 (−10.37, 8.76)
−1.26 (−3.43, 1.03)	4.03 (−1.01, 9.23)	−0.04 (−5.36, 5.61)	−0.62 (−8.58, 7.5)	0.59 (−5.68, 6.99)	Curcumin	1.81 (−5.36, 9.19)	**6.62 (0.82, 12.26)**	−0.12 (−5.12, 5)	−4.72 (−12.49, 3.12)	−0.3 (−4.39, 3.91)	−0.02 (−7.26, 7.37)	−0.7 (−9.49, 8.16)	−0.22 (−7.98, 7.66)
−3.08 (−10.03, 3.85)	2.2 (−6.11, 10.54)	−1.86 (−10.27, 6.77)	−2.43 (−12.8, 7.92)	−1.24 (−10.36, 7.93)	−1.81 (−9.19, 5.36)	Naringenin	4.8 (−4.05, 13.37)	−1.94 (−10.17, 6.32)	−6.55 (−16.73, 3.6)	−2.14 (−9.88, 5.65)	−1.86 (−11.68, 8)	−2.52 (−13.51, 8.39)	−2.05 (−12.25, 8.17)
**−7.88 (−13.03, −2.48)**	−2.6 (−9.44, 4.54)	−6.65 (−13.73, 0.81)	−7.23 (−16.45, 2.23)	−6.04 (−13.86, 2.04)	**−6.62 (−12.26, −0.82)**	−4.8 (−13.37, 4.05)	Catechin	−6.74 (−13.57, 0.3)	**−11.36 (−20.36, −2.13)**	**−6.93 (−13.09, −0.48)**	−6.63 (−15.25, 2.25)	−7.32 (−17.24, 2.8)	−6.83 (−15.9, 2.38)
−1.13 (−5.69, 3.38)	4.16 (−2.34, 10.59)	0.09 (−6.6, 6.89)	−0.49 (−9.43, 8.41)	0.71 (−6.81, 8.19)	0.12 (−5, 5.12)	1.94 (−6.32, 10.17)	6.74 (−0.3, 13.57)	Resveratrol	−4.62 (−13.35, 4.1)	−0.18 (−5.93, 5.54)	0.1 (−8.18, 8.42)	−0.59 (−10.29, 9.07)	−0.1 (−8.88, 8.61)
3.47 (−3.99, 10.97)	**8.77 (0.02, 17.57)**	4.7 (−4.2, 13.8)	4.13 (−6.62, 14.91)	5.32 (−4.26, 14.87)	4.72 (−3.12, 12.49)	6.55 (−3.6, 16.73)	**11.36 (2.13, 20.36)**	4.62 (−4.1, 13.35)	DHM	4.42 (−3.8, 12.72)	4.73 (−5.54, 14.99)	4.03 (−7.26, 15.38)	4.49 (−6.04, 15.05)
−0.96 (−4.46, 2.54)	4.33 (−1.48, 10.13)	0.26 (−5.73, 6.49)	−0.32 (−8.79, 8.17)	0.87 (−6, 7.83)	0.3 (−3.91, 4.39)	2.14 (−5.65, 9.88)	**6.93 (0.48, 13.09)**	0.18 (−5.54, 5.93)	−4.42 (−12.72, 3.8)	Silymarin	0.28 (−7.5, 8.13)	−0.42 (−9.67, 8.84)	0.07 (−8.22, 8.36)
−1.24 (−8.23, 5.7)	4.06 (−4.34, 12.4)	−0.02 (−8.49, 8.7)	−0.61 (−11.02, 9.8)	0.58 (−8.56, 9.81)	0.02 (−7.37, 7.26)	1.86 (−8, 11.68)	6.63 (−2.25, 15.25)	−0.1 (−8.42, 8.18)	−4.73 (−14.99, 5.54)	−0.28 (−8.13, 7.5)	GA and CA	−0.69 (−11.71, 10.32)	−0.22 (−10.44, 10.03)
−0.55 (−9.1, 8)	4.74 (−4.97, 14.46)	0.67 (−9.14, 10.63)	0.09 (−11.43, 11.6)	1.28 (−9.08, 11.72)	0.7 (−8.16, 9.49)	2.52 (−8.39, 13.51)	7.32 (−2.8, 17.24)	0.59 (−9.07, 10.29)	−4.03 (−15.38, 7.26)	0.42 (−8.84, 9.67)	0.69 (−10.32, 11.71)	Hesperidin	0.48 (−10.93, 11.81)
−1.03 (−8.53, 6.46)	4.27 (−4.53, 13.04)	0.18 (−8.72, 9.3)	−0.39 (−11.15, 10.41)	0.82 (−8.76, 10.37)	0.22 (−7.66, 7.98)	2.05 (−8.17, 12.25)	6.83 (−2.38, 15.9)	0.1 (−8.61, 8.88)	−4.49 (−15.05, 6.04)	−0.07 (−8.36, 8.22)	0.22 (−10.03, 10.44)	−0.48 (−11.81, 10.93)	Quercetin

^#Bold font represents a statistical difference. The blue module represents different dietary polyphenols. The green module represents row-column comparisons of intervention effects of different dietary polyphenols. The orange module represents column-row comparisons of intervention effects of different dietary polyphenols.^

No statistical differences were noted in the impact of these dietary polyphenols on LDL-C levels (Table 7). The SUCRA probability ranking results for lowering serum LDL-C levels indicated that DHM (73.22%) was more effective than GA and CA (71.95%), followed by naringenin (66.44%) (Figure 4).

**Table 7:** League table of LDL-C of different dietary polyphenols.

Placebo	EA	Anthocyanin	Genistin/Genistein	CA	Curcumin	Naringenin	Catechin	Resveratrol	DHM	Silymarin	GA and CA	Hesperidin	Quercetin
Placebo	−8.83 (−29.38, 11.67)	−9.16 (−27.72, 9.71)	−0.49 (−30.82, 30.06)	1.61 (−20.86, 24.08)	−8.47 (−17.52, 0.78)	−16.02 (−46.07, 14.03)	−10.84 (−31.95, 10.85)	4.58 (−10.56, 20.06)	−19.77 (−50.14, 10.68)	−7.34 (−23.52, 8.69)	−19.13 (−50.24, 11.87)	−13.1 (−45.41, 19.16)	−13.65 (−43.27, 15.86)
8.83 (−11.67, 29.38)	EA	−0.37 (−27.88, 27.58)	8.39 (−28.39, 45.06)	10.49 (−20.06, 40.83)	0.33 (−22, 22.92)	−7.19 (−43.83, 29.15)	−2 (−31.54, 27.82)	13.36 (−12.01, 39.27)	−10.92 (−47.89, 25.83)	1.47 (−24.44, 27.56)	−10.31 (−47.42, 27.05)	−4.25 (−42.39, 33.9)	−4.81 (−40.86, 30.92)
9.16 (−9.71, 27.72)	0.37 (−27.58, 27.88)	Anthocyanin	8.69 (−27.14, 44.28)	10.87 (−18.69, 39.9)	0.69 (−20.2, 21.44)	−6.87 (−42.3, 28.5)	−1.68 (−30.19, 26.79)	13.74 (−10.28, 38.03)	−10.59 (−46.38, 24.98)	1.8 (−23.02, 26.48)	−10.01 (−46.32, 26.11)	−3.89 (−41.38, 33.26)	−4.52 (−39.63, 30.46)
0.49 (−30.06, 30.82)	−8.39 (−45.06, 28.39)	−8.69 (−44.28, 27.14)	Genistin/Genistein	2.12 (−35.79, 39.95)	−7.97 (−39.74, 23.85)	−15.59 (−58.43, 27.32)	−10.31 (−47.39, 26.98)	5.06 (−28.84, 39.29)	−19.27 (−62.58, 23.69)	−6.86 (−41.57, 27.48)	−18.67 (−62.11, 24.69)	−12.55 (−57.12, 31.62)	−13.23 (−55.7, 29.28)
−1.61 (−24.08, 20.86)	−10.49 (−40.83, 20.06)	−10.87 (−39.9, 18.69)	−2.12 (−39.95, 35.79)	CA	−10.14 (−34.22, 14.31)	−17.66 (−55.28, 20)	−12.49 (−43.33, 18.71)	2.92 (−24.02, 30.41)	−21.38 (−59.14, 16.48)	−8.99 (−36.63, 18.62)	−20.78 (−59.06, 17.67)	−14.64 (−54.16, 24.54)	−15.35 (−52.6, 21.89)
8.47 (−0.78, 17.52)	−0.33 (−22.92, 22)	−0.69 (−21.44, 20.2)	7.97 (−23.85, 39.74)	10.14 (−14.31, 34.22)	Curcumin	−7.58 (−39.07, 23.73)	−2.37 (−25.58, 20.98)	13.05 (−4.64, 30.82)	−11.29 (−43.27, 20.43)	1.11 (−17.61, 19.53)	−10.69 (−43.09, 21.55)	−4.6 (−38.31, 28.8)	−5.17 (−36.38, 25.6)
16.02 (−14.03, 46.07)	7.19 (−29.15, 43.83)	6.87 (−28.5, 42.3)	15.59 (−27.32, 58.43)	17.66 (−20, 55.28)	7.58 (−23.73, 39.07)	Naringenin	5.15 (−31.48, 42.4)	20.57 (−13.03, 54.53)	−3.72 (−46.45, 39.01)	8.68 (−25.41, 42.68)	−3.13 (−46.23, 40.09)	3 (−41.14, 47.07)	2.37 (−39.96, 44.54)
10.84 (−10.85, 31.95)	2 (−27.82, 31.54)	1.68 (−26.79, 30.19)	10.31 (−26.98, 47.39)	12.49 (−18.71, 43.33)	2.37 (−20.98, 25.58)	−5.15 (−42.4, 31.48)	Catechin	15.41 (−10.87, 41.79)	−8.94 (−46.45, 27.89)	3.46 (−23.31, 30.14)	−8.32 (−45.95, 29.2)	−2.29 (−41.05, 36.21)	−2.86 (−39.54, 33.56)
−4.58 (−20.06, 10.56)	−13.36 (−39.27, 12.01)	−13.74 (−38.03, 10.28)	−5.06 (−39.29, 28.84)	−2.92 (−30.41, 24.02)	−13.05 (−30.82, 4.64)	−20.57 (−54.53, 13.03)	−15.41 (−41.79, 10.87)	Resveratrol	−24.3 (−58.66, 9.47)	−11.92 (−34.29, 9.93)	−23.68 (−58.38, 10.82)	−17.67 (−53.44, 17.89)	−18.21 (−51.83, 14.87)
19.77 (−10.68, 50.14)	10.92 (−25.83, 47.89)	10.59 (−24.98, 46.38)	19.27 (−23.69, 62.58)	21.38 (−16.48, 59.14)	11.29 (−20.43, 43.27)	3.72 (−39.01, 46.45)	8.94 (−27.89, 46.45)	24.3 (−9.47, 58.66)	DHM	12.37 (−22, 46.82)	0.58 (−42.53, 44.54)	6.65 (−37.49, 50.92)	6 (−36.37, 48.74)
7.34 (−8.69, 23.52)	−1.47 (−27.56, 24.44)	−1.8 (−26.48, 23.02)	6.86 (−27.48, 41.57)	8.99 (−18.62, 36.63)	−1.11 (−19.53, 17.61)	−8.68 (−42.68, 25.41)	−3.46 (−30.14, 23.31)	11.92 (−9.93, 34.29)	−12.37 (−46.82, 22)	Silymarin	−11.73 (−46.7, 23.14)	−5.7 (−41.93, 30.25)	−6.33 (−39.92, 27.16)
19.13 (−11.87, 50.24)	10.31 (−27.05, 47.42)	10.01 (−26.11, 46.32)	18.67 (−24.69, 62.11)	20.78 (−17.67, 59.06)	10.69 (−21.55, 43.09)	3.13 (−40.09, 46.23)	8.32 (−29.2, 45.95)	23.68 (−10.82, 58.38)	−0.58 (−44.54, 42.53)	11.73 (−23.14, 46.7)	GA and CA	6.04 (−38.59, 50.8)	5.47 (−37.33, 48.45)
13.1 (−19.16, 45.41)	4.25 (−33.9, 42.39)	3.89 (−33.26, 41.38)	12.55 (−31.62, 57.12)	14.64 (−24.54, 54.16)	4.6 (−28.8, 38.31)	−3 (−47.07, 41.14)	2.29 (−36.21, 41.05)	17.67 (−17.89, 53.44)	−6.65 (−50.92, 37.49)	5.7 (−30.25, 41.93)	−6.04 (−50.8, 38.59)	Hesperidin	−0.61 (−44.36, 43.15)
13.65 (−15.86, 43.27)	4.81 (−30.92, 40.86)	4.52 (−30.46, 39.63)	13.23 (−29.28, 55.7)	15.35 (−21.89, 52.6)	5.17 (−25.6, 36.38)	−2.37 (−44.54, 39.96)	2.86 (−33.56, 39.54)	18.21 (−14.87, 51.83)	−6 (−48.74, 36.37)	6.33 (−27.16, 39.92)	−5.47 (−48.45, 37.33)	0.61 (−43.15, 44.36)	Quercetin

The blue module represents different dietary polyphenols. The green module represents row-column comparisons of intervention effects of different dietary polyphenols. The orange module represents column-row comparisons of intervention effects of different dietary polyphenols.

#### 3.3.6 Inflammation-related outcome measures

Twelve studies reported serum TNF-α levels after dietary polyphenol treatment for NAFLD.

The NMA results demonstrated that quercetin was superior to placebo [WMD = 51.61, 95% CI = (22.27, 80.77)], genistin/genistein [WMD = 34.71, 95% CI = (0.55, 68.49)], CA [WMD = 50, 95% CI = (17.96, 82.03)], curcumin [WMD = 46.59, 95% CI = (15.99, 76.87)], resveratrol [WMD = 50.07, 95% CI = (19.81, 79.9)], DHM [WMD = 50.29, 95% CI = (18.39, 82.18)], and hesperidin [WMD = 45.29, 95% CI = (13.02, 77.39)] in reducing TNF-α (Table 8). According to the SUCRA probability ranking for lowering serum TNF-α levels, quercetin (99.47%) was ranked highest, followed by genistin/genistein (79.74%) and hesperidin (55.45%) (Figure 4).

**Table 8:** League table of TNF-α of different dietary polyphenols.

Placebo	Genistin/Genistein	CA	Curcumin	Resveratrol	DHM	Hesperidin	Quercetin
Placebo	−16.91 (−34.14, 0.22)	−1.58 (−14.87, 11.92)	−4.92 (−13.93, 3.27)	−1.46 (−8.75, 4.92)	−1.32 (−14.38, 11.9)	−6.33 (−19.97, 7.37)	**−51.61 (−80.77, −22.27)**
16.91 (−0.22, 34.14)	Genistin/Genistein	15.36 (−6.22, 37.19)	11.97 (−7.42, 30.79)	15.38 (−3.36, 33.65)	15.59 (−5.81, 37.3)	10.58 (−11.21, 32.49)	**−34.71 (−68.49, −0.55)**
1.58 (−11.92, 14.87)	−15.36 (−37.19, 6.22)	CA	−3.36 (−19.71, 11.97)	0.11 (−15.5, 14.62)	0.25 (−18.54, 19.04)	−4.77 (−23.91, 14.41)	**−50 (−82.03, −17.96)**
4.92 (−3.27, 13.93)	−11.97 (−30.79, 7.42)	3.36 (−11.97, 19.71)	Curcumin	3.48 (−7.52, 14.36)	3.58 (−11.64, 19.85)	−1.4 (−17.1, 15.1)	**−46.59 (−76.87, −15.99)**
1.46 (−4.92, 8.75)	−15.38 (−33.65, 3.36)	−0.11 (−14.62, 15.5)	−3.48 (−14.36, 7.52)	Resveratrol	0.13 (−14.24, 15.47)	−4.86 (−19.75, 10.96)	**−50.07 (−79.9, −19.81)**
1.32 (−11.9, 14.38)	−15.59 (−37.3, 5.81)	−0.25 (−19.04, 18.54)	−3.58 (−19.85, 11.64)	−0.13 (−15.47, 14.24)	DHM	−5.04 (−24.01, 13.98)	**−50.29 (−82.18, −18.39)**
6.33 (−7.37, 19.97)	−10.58 (−32.49, 11.21)	4.77 (−14.41, 23.91)	1.4 (−15.1, 17.1)	4.86 (−10.96, 19.75)	5.04 (−13.98, 24.01)	Hesperidin	**−45.29 (−77.39, −13.02)**
**51.61 (22.27, 80.77)**	**34.71 (0.55, 68.49)**	**50 (17.96, 82.03)**	**46.59 (15.99, 76.87)**	**50.07 (19.81, 79.9)**	**50.29 (18.39, 82.18)**	**45.29 (13.02, 77.39)**	Quercetin

^#Bold font represents a statistical difference. The blue module represents different dietary polyphenols. The green module represents row-column comparisons of intervention effects of different dietary polyphenols. The orange module represents column-row comparisons of intervention effects of different dietary polyphenols.^

### 3.4 Publication bias

**Figure 5:**
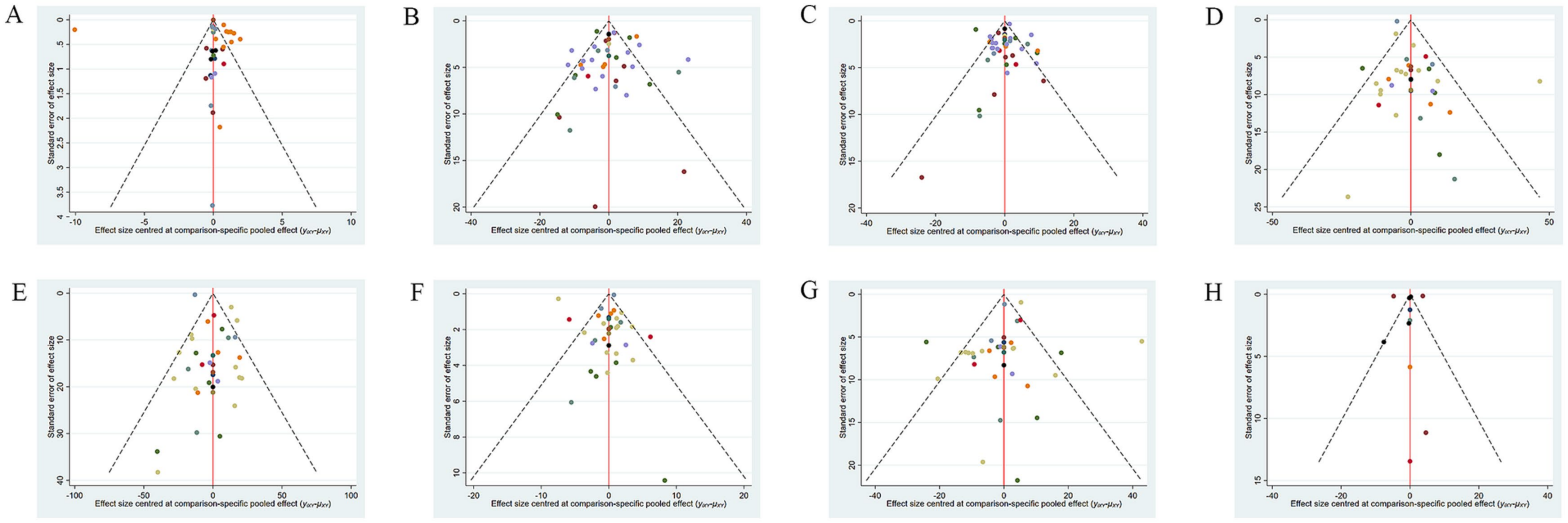
Funnel plot of different outcome indicators of NAFLD patients treated with different dietary polyphenol supplements. **(A)** BMI; **(B)** ALT; **(C)** AST; **(D)** TC; **(E)** TG; **(F)** HDL-C; **(G)** LDL-C; **(H)** TNF-α.

A corrected comparison funnel plot was created for each outcome measure to appraise publication bias. Upon visual inspection of the funnel plot, no notable publication bias was detected (Figure 5). Funnel plots for publication bias of the other measures are provided in Supplementary material 3.

## 4 Discussion

The effects of 13 various single (or combined) dietary polyphenol supplements on NAFLD treatment are compared in this study. The results indicate that naringenin is the most effective polyphenol for lowering serum TC and TG, catechin for reducing BMI and ALT, AST, ALP (98.65%) and raising HDL-C, DHM for reducing LDL-C, FBG (82.80%), and insulin levels (77.34%) and quercetin for lowering TNF-α. In conclusion, our findings suggest that catechin may be the most appropriate dietary polyphenol supplement for treating patients with NAFLD.

Catechin is a naturally occurring polyphenolic compound found in dried tea leaves and is the main bioactive ingredient in green tea extracts. The results of this study imply that catechin is the most effective in reducing BMI, consistent with previous studies (14, 77). BMI is a common measure of overweight/obesity, and individuals with these conditions are more likely to develop NAFLD (78). Epigallocatechin gallate (EGCG) is the most abundant (60.89%) effective catechin in green tea (79). Animal studies (80, 81) indicate that EGCG reduces body weight by (i) decreasing intestinal absorption of lipids and proteins, reducing caloric intake, and (ii) activating adenosine monophosphate-activated protein kinase (AMPK), which regulates lipid, cholesterol, and glucose metabolism in the liver, skeletal muscles, and white adipose tissue, thereby promoting lipid and glucose catabolism (82).

Besides reducing BMI, catechin can regulate serum lipids in NAFLD patients by lowering TC levels and raising HDL-C levels. The liver is the primary organ responsible for regulating lipid metabolism. High TC levels are positively tied to NAFLD onset (83), while low HDL-C levels are a risk factor for both NAFLD and severe liver diseases (83, 84). Moreover, the TC/HDL-C ratio serves as a more reliable predictor of NAFLD. A higher ratio indicates a greater risk of BMI-related NAFLD (83, 85). Catechin can increase HDL-C levels in overweight/obese individuals (86). In addition, the consumption of green tea drinks or extracts can notably reduce serum TC levels in adults (87), demonstrating the role of catechins in lipid metabolism regulation. An animal experiment suggests that EGCG can inhibit hepatic cholesterol synthesis by modulating the sterol regulatory element-binding protein 2/silent information regulator 1 (SIRT1)/forkhead box transcription factor O1 signaling pathway, thereby affecting lipid metabolism in hyperlipidemic rats (88). Moreover, oxidative stress is a core mechanism in the progression of NAFLD. Nuclear factor erythroid 2-related factor 2 (NRF2) is a key factor that limits oxidative stress through its transcriptional activity and can regulate the expression of lipid metabolism-related genes, while EGCG can notably inhibit oxidative stress via the NRF2 signaling pathway (89).

ALT, AST, and ALP serve as traditional blood biomarkers for evaluating liver cell damage. Elevated levels of AST and ALT indicate hepatocellular necrosis, while elevated ALP levels suggest damage to biliary epithelial cells or tubular membranes (90). All three may be linked to the risk of HCC development (91). Multiple animal studies have confirmed that EGCG intervention can reduce serum liver enzyme levels in NAFLD/NASH model animals. The action mechanism is multifaceted, including the improvement of insulin sensitivity, facilitation of glucose metabolism in liver tissue, and regulation of the expression of inflammatory mediators and related pathological changes (89, 92, 93). Moreover, catechin is a natural iron chelator that can restrict iron absorption, potentially helping to prevent liver iron accumulation and slow the progression of NASH (59, 62, 94).

This study includes four RCTs on catechin treatment for NAFLD. One study notes mild bloating in NAFLD patients who take green tea extract. However, it does not specify whether this is linked to the green tea extract (61). Another article states that taking a high dose of catechin (1,080 mg/700 mL) does not induce any side effects (59). Although high-dose catechin may offer more benefits in reducing patients’ weight, BMI, and ALT (59, 95), it may also lead to mild nausea and stomach pain, particularly when taken on an empty stomach (89). Basic research also reveals that high doses of EGCG can result in mild liver damage in mice (96) and concentration-dependent hepatocyte death (97). Hence, the safety dosage of catechin still requires further research to be determined. Furthermore, the bioavailability of catechins is influenced by various processes, such as chemical degradation, microbial metabolism, and hepatic metabolism. Consequently, only a small fraction of the catechins that enter the intestine after tea consumption are absorbed (98). This issue warrants further attention.

High levels of TC and TG are connected to the development of NAFLD (83, 84). Naringenin is a natural citrus flavonoid that is widely present in grapefruit and oranges. It is the most effective dietary polyphenol in this study for decreasing serum TC and TG, aligning with prior research results (77). According to basic research results, the mechanisms through which naringenin reduces TC and TG levels in NAFLD/NASH rodent models are outlined below: (i) The key regulatory enzyme that inhibits cholesterol synthesis, 3-hydroxy-3-methylglutaryl-coenzyme A reductase is involved in lipid metabolism (99). (ii) By inhibiting the activation of the NLR family pyrin domain containing 3 inflammasome in hepatocytes, inflammation and lipid accumulation are alleviated (100). (iii) By activating the SIRT1-mediated signaling cascade, naringenin regulates the expression of lipid metabolism-related genes (fatty acid synthase, stearoyl-CoA desaturase 1, peroxisome proliferator-activated receptor α, carnitine palmitoyltransferase 1α) and clears reactive oxygen species and lipid peroxides in the body (101). (iv) By downregulating the liver X receptor in the liver and activating AMPK, naringenin stimulates fatty acid oxidation and inhibits lipogenesis (102). Additionally, since gut bacteria can influence host lipid metabolism, naringenin can lower TC and TG levels in NASH mice by regulating the gut microbiota (103). Two included RCTs on naringenin for NAFLD show no side effects (71, 72). An animal study indicates that different concentrations of naringenin (1 mM, 10 mM, 100 mM) do not cause intestinal damage in rats (104). However, there are contradictory research results (105). Consequently, the safety of naringenin must be explored in more detail. Moreover, although naringenin can be rapidly absorbed after oral administration, its bioavailability is only 15%. This low bioavailability is attributed to its very low affinity for water and the prolonged metabolic time during intestinal passage (106, 107).

DHM is the primary flavonoid substance in the edible medicinal plant *Ampelopsis grossedentata*. In this study, it is the most effective dietary polyphenol in reducing serum LDL-C, FBG, and insulin levels. High serum levels of LDL-C are the primary cause of the development of atherosclerosis (108). NAFLD is prevalent in T2DM patients (109). Chen et al. (73) propose that DHM lowers LDL-C, FBG, and insulin by regulating fibroblast growth factor 21 (FGF21) to improve IR. FGF21 is expressed in the liver, adipose tissue, and pancreas, playing a key role in the regulation of glucose and lipid metabolism. It may potentially develop into a biomarker for monitoring NAFLD/NASH (73, 110). Basic research indicates that DHM is a potential agonist of peroxisome proliferator-activated receptor γ (PPARγ) (112), an important metabolic regulatory factor for targeted treatment of NAFLD (111). DHM improves IR by activating PPARγ and subsequently modulating the FGF21-AMPK pathway (112). No toxicity has been reported for DHM within the typical dosage range. The only included RCT on DHM treatment for NAFLD shows that participants taking 600mg of DHM capsules daily experience no adverse effects (73). A study demonstrates that mice with NAFLD receive oral doses of DHM at 500 mg/kg/day, 750 mg/kg/day, and 1000 mg/kg/day for 8 weeks, with no adverse reactions reported (113). Moreover, an acute toxicity test on DHM reveals that its toxicity is very slight, the highest tolerance level for oral gavage in rats is 5g/kg body weight (114). The maximum safe dose for mice is around 16 g/kg, while for adults, it is approximately 1.6 g/kg, based on the body surface area standardization method (115). This could serve as a reference for determining the safe dosage of DHM. The absolute bioavailability of DHM in rats is less than 10%, which may be due to its poor solubility in water, as well as its metabolism and elimination within the intestinal tract (116).

Quercetin, a natural flavonoid found abundantly in various foods (like apples, onions, and tomatoes), possesses potent anti-inflammatory capabilities. It has the greatest effect in reducing TNF-α in NAFLD patients. Inflammation is regarded as a critical driver of NAFLD progression and can promote hepatic steatosis (100). Furthermore, TNF-α can mediate inflammation by activating mitogen-activated protein kinases (MAPK) signaling pathways, like extracellular signal-regulated kinase, c-Jun N-terminal kinase, nuclear factor kappa-B (NF-κB), and activator protein 1 (AP-1) (75). Nevertheless, quercetin can block the TNF-α-induced inflammatory cascade by inhibiting MAPK or enhancing PPARγ activity, thus antagonizing NF-κB or AP-1 activation and indirectly reducing inflammation (117, 118). The sole RCT included on quercetin treatment for NAFLD involves patients taking 500 mg of quercetin daily for 12 weeks, and no adverse effects are observed (75). Other clinical studies report minimal adverse effects of quercetin. Nonetheless, the safety of long-term (>12 weeks) high-dose (≥1,000 mg) supplementation needs further evaluation (119). A pharmacokinetic study indicates that the bioavailability of a single oral dose of quercetin is very low (approximately 2%). This may be ascribed to factors like reduced absorption rates, extensive metabolism, and/or rapid elimination (117).

The MD has been proven to be a healthy eating pattern that can improve blood lipids and liver enzymes, and reverse IR in patients with NAFLD (120–122). The MD is marked by food diversity, including fruits, vegetables, legumes, spices, and herbs. The health benefits are attributed to its rich content of dietary polyphenols with anti-inflammatory and antioxidant effects (123, 124). Given the absence of consistent evidence on the effective dosage and safety of isolated dietary polyphenol monomers, it is recommended for NAFLD patients to follow the MD, which is a safer, more affordable, and easier-to-maintain adjunctive therapy. However, before starting the MD, it is still important to seek the advice and guidance of a nutritionist or doctor based on one’s health condition to ensure safety and effectiveness.

This study compared the effects of different dietary polyphenol supplements on NAFLD, including 54 RCTs with 3,132 patients, offering a large sample size. Nonetheless, this study also presents certain limitations. Firstly, the network diagram has not formed a loop, and there is insufficient evidence for direct comparisons of various dietary polyphenols. Secondly, although this study shows that DHM and quercetin have markable effects, the number of samples available for research is very limited. Finally, the literature included in this study exhibits an imbalance in regional distribution. There are differences among various regions regarding healthcare systems, methods for diagnosing and assessing NAFLD, as well as laboratory testing indicators. The above limitations could impact the reliability of the findings in this study.

## 5 Conclusion

This study indicates that catechin, naringenin, DHM, and quercetin are dietary polyphenols that demonstrate notable efficacy in improving metabolic and inflammatory markers in patients with NAFLD. Among these, catechin appears to comprehensively enhance BMI, liver enzymes, and blood lipids linked to NAFLD, suggesting it may be the most suitable dietary polyphenol supplement. These findings provide preliminary recommendations and reliable evidence for healthcare professionals or nutritionists advocating for the use of dietary polyphenol supplements or polyphenol-rich diets as adjunctive therapies for NAFLD. The focus of future studies may, on the one hand, still be based on well-designed and rigorous RCTs to provide adequate sample sizes that can be employed for analyses. This is particularly important for dietary polyphenols that the current study has identified as most efficacious but which are limited by small sample sizes (like DHM and quercetin). On the other hand, given the uncertainties surrounding the safety of dietary polyphenols and their relatively low bioavailability, it may be beneficial to utilize RCTs to examine the incidence of adverse reactions tied to these compounds in order to better understand their safety profiles. Or, conducting a meta-analysis of clinical studies related to specific dietary polyphenols could help summarize effective strategies for maximizing their bioavailability in humans, determining the lowest effective doses, and addressing long-term administration safety concerns.

## Footnotes

^1^ https://www.crd.york.ac.uk/PROSPERO/

^2^ http://phenol-explorer.eu/
