# Supplementary material for: Efficacy of dietary polyphenol supplement in patients with non-alcoholic fatty liver disease: a network meta-analysis
Source: Front Nutr. 2025 May 9;12:1582861. doi: 10.3389/fnut.2025.1582861 (PMC12100629; doi:10.3389/fnut.2025.1582861)
Supplement: Supplementary file 1 [file Table_1.docx]

# Supplementary Material 1: Search Strategies for database (Pubmed as an example)

| Search number | Query | Results |
| --- | --- | --- |
| #1 | "Polyphenols"[Mesh] | 33,024 |
| #2 | (polyphenol[Title/Abstract]) OR (polyphenols[Title/Abstract])) OR (provinols[Title/Abstract])) OR (flavonoid[Title/Abstract])) OR (flavone[Title/Abstract])) OR (flavanone[Title/Abstract])) OR (flavonol[Title/Abstract])) OR (isoflavone[Title/Abstract])) OR (isoflavonoid[Title/Abstract])) OR (anthocyanin[Title/Abstract])) OR (anthocyanins[Title/Abstract])) OR (proanthocyanidin[Title/Abstract])) OR (proanthocyanidins[Title/Abstract])) OR (phenolic acid[Title/Abstract])) OR (phenolic acids[Title/Abstract])) OR (phenol[Title/Abstract])) OR (lignans[Title/Abstract])) OR (enterolignan[Title/Abstract])) OR (stilbene[Title/Abstract])) OR (stilbenes[Title/Abstract])) OR (curcumin[Title/Abstract])) OR (catechin[Title/Abstract])) OR (silymarin[Title/Abstract])) OR (hesperetin[Title/Abstract])) OR (hesperidin[Title/Abstract])) OR (naringenin[Title/Abstract])) OR (kaempferol[Title/Abstract])) OR (quercetin[Title/Abstract])) OR (resveratrol[Title/Abstract])) OR (phytoestrogen[Title/Abstract])) OR (genistein[Title/Abstract])) OR (matairesinol[Title/Abstract])) OR (secoisolariciresinol[Title/Abstract])) OR (myricetin[Title/Abstract])) OR (lariciresinol[Title/Abstract])) OR (pinoresinol[Title/Abstract])) OR (enterodiol[Title/Abstract])) OR (enterolactone[Title/Abstract])) OR (coumestrol[Title/Abstract])) OR (tamarixetin[Title/Abstract])) OR (luteolin[Title/Abstract])) OR (apigenin[Title/Abstract])) OR (fruit[Title/Abstract])) OR (cocoa[Title/Abstract])) OR (coffee[Title/Abstract])) OR (tea[Title/Abstract])) OR (nut[Title/Abstract])) OR (juice[Title/Abstract])) OR (wine[Title/Abstract])) OR (isorhamnetin[Title/Abstract])) OR (eriodictyol[Title/Abstract])) OR (flavan-3-ol[Title/Abstract])) OR (epicatechin[Title/Abstract])) OR (theaflavin[Title/Abstract])) OR (anthocyanidin[Title/Abstract])) OR (cyanidin[Title/Abstract])) OR (delphinidin[Title/Abstract])) OR (malvidin[Title/Abstract])) OR (peonidin[Title/Abstract])) OR (petunidin[Title/Abstract])) OR (benzoflavones[Title/Abstract])) OR (beta naphthoflavone[Title/Abstract])) OR (bioflavonoids[Title/Abstract])) OR (chalcones[Title/Abstract])) OR (diosmin[Title/Abstract])) OR (rutin[Title/Abstract])) OR (pterocarpum[Title/Abstract])) OR (rotenone[Title/Abstract])) OR (phloretin[Title/Abstract])) OR (polyphloretin phosphate[Title/Abstract])) OR (hydroxycinnamic acids[Title/Abstract])) OR (hydroxybenzoic acids[Title/Abstract])) OR (hydroxyphenylacetic acids[Title/Abstract])) OR (alkylphenols[Title/Abstract])) OR (alkylmethoxyphenols[Title/Abstract])) OR (hesperedin[Title/Abstract]) | 432,424 |
| #3 | #1 OR #2 | 438,389 |
| #4 | "Non-alcoholic Fatty Liver Disease"[Mesh] | 26,378 |
| #5 | ((((((((((((((((((non-alcoholic fatty liver disease[Title/Abstract]) OR (Nonalcoholic Fatty Liver[Title/Abstract])) OR (Non alcoholic Fatty Liver Disease[Title/Abstract])) OR (NAFLD[Title/Abstract])) OR (Nonalcoholic Fatty Liver Disease[Title/Abstract])) OR (Nonalcoholic Fatty Livers[Title/Abstract])) OR (Nonalcoholic Steatohepatitis[Title/Abstract])) OR (Nonalcoholic Steatohepatitides[Title/Abstract])) OR (non alcoholic hepato-steatosis[Title/Abstract])) OR (non alcoholic hepatosteatosis[Title/Abstract])) OR (non alcoholic liver steatosis[Title/Abstract])) OR (non alcoholic steatotic hepatopathy[Title/Abstract])) OR (non-alcoholic FLD[Title/Abstract])) OR (non-alcoholic hepatic steatosis[Title/Abstract])) OR (nonalcoholic FLD[Title/Abstract])) OR (nonalcoholic hepatic steatosis[Title/Abstract])) OR (nonalcoholic hepatosteatosis[Title/Abstract])) OR (nonalcoholic liver steatosis'[Title/Abstract])) OR (nonalcoholic fatty liver'[Title/Abstract]) | 40,602 |
| #6 | #4 OR #5 | 44,148 |
| #7 | #3 AND #6 | 1,603 |
