# Supplementary material for: Efficacy of dietary polyphenol supplement in patients with non-alcoholic fatty liver disease: a network meta-analysis
Source: Front Nutr. 2025 May 9;12:1582861. doi: 10.3389/fnut.2025.1582861 (PMC12100629; doi:10.3389/fnut.2025.1582861)
Supplement: Supplementary file 2 [file Table_2.docx]

# Supplementary materials 2 Basic characteristics of 54 included RCTs

| Polyphenol | Author | Country | Year | analyzable sample size | | Gender# | | Mean age(years) | | Intervention | | Treatment duration(weeks) | Outcomes |
| --- | --- | --- | --- | --- | --- | --- | --- | --- | --- | --- | --- | --- | --- |
|  |  |  |  | Trial group | Control group | Trial group(M/F) | Control group(M/F) | Trial group | Control group | Trial group | Control group |  |  |
| Curcumin | △Saeede Saadati[23] | Iran | 2019 | 27 | 23 | 13/14 | 14/9 | 46.19 ± 11.5 | 45.13 ± 10.9 | Curcumin 1,500mg | Placebo | 12 | Weight BMI WC HC WHR ALT AST GGT TG TC HDL-C LDL-C FBG Insulin HOMA-IR |
|  | ＊Ghanashyam Patel[24] | India | 2023 | 15 | 15 | 12/3 | 12/3 | 31.4±6.08 | 36.9±10.50 | *Curcuma longa* extract capsules 500 mg | Placebo | 8 | ALT AST TC |
|  | ＊Yunes Panahi[25] | Iran | 2019 | 35 | 35 | 20/15 | 19/16 | 46.63 ± 2.21 | 47.51 ± 2.45 | Curcumin 500 mg plus piperine 5 mg | Placebo | 12 | ALT AST ALP TG TC HDL-C LDL-C FBG |
|  | △Maryam Saberi-Karimian[26] | Iran | 2020 | 27 | 28 | NA | NA | aged 18–70 years | | Curcuminoids 500 mg plus piperine 5 mg | Placebo | 8 | Weight BMI WC SBP DBP ALT AST TG TC HDL-C LDL-C FBG TNF-α |
|  | △Seyed Reza Mirhafez[27] | Iran | 2021 | 33 | 37 | 18/15 | 22/15 | 45.6 ± 11.0 | 43.1 ± 11.6 | Curcuminoids 500 mg plus piperine 5 mg | Placebo | 8 | Weight BMI HC WHR SBP DBP ALT AST ALP TG TC HDL-C LDL-C FBG |
|  | △Shima Sharifi[28] | Iran | 2023 | 30 | 30 | 20/10 | 16/14 | 43 ± 9.72 | 47.5 ± 13.31 | Curcumin 500 mg plus piperine 5 mg | Placebo | 12 | Weight BMI WC SBP DBP ALT AST TG TC HDL-C LDL-C FBG |
|  | △Leila Rezaei[29] | Iran | 2023 | 6 | 6 | 0/6 | 0/6 | 44.33±8.55 | | Nano-curcumin 80 mg plus pilates | Placebo plus pilates | 8 | Weight BMI WHR ALT AST GGT ALP TG TC HDL-C LDL-C |
|  | △Seyed Ali Jazayeri-Tehrani[30] | Iran | 2019 | 42 | 42 | 23/19 | 23/19 | 41.8±5.6 | 42.5±6.2 | Nano-curcumin 80 mg | Placebo | 12 | Weight BMI WC SBP DBP ALT AST TG TC HDL-C LDL-C FBG Insulin HOMA-IR TNF-α |
|  | △Baharak Moradi Kelardeh[31] | Iran | 2020 | 11 | 11 | 0/11 | 0/11 | 66.72±3.03 | 64.36±2.97 | Nano-curcumin 80 mg | Placebo | 12 | Weight BMI ALT AST ALP |
|  | △Yunes Panahi[32] | Iran | 2016 | 44 | 43 | 24/20 | 27/16 | 44.98±12.59 | 47.21±10.29 | Curcumin 1,000 mg | Placebo | 8 | TG TC HDL-C LDL-C FBG Insulin HOMA-IR |
|  | △Yunes Panahi[33] | Iran | 2017 | 44 | 43 | 24/20 | 27/16 | 44.98 ± 12.59 | 47.21 ± 10.29 | Curcumin 1,000 mg | Placebo | 8 | BMI WC SBP DBP ALT AST ALP |
|  | △Seyed Reza Mirhafez[34] | Iran | 2021 | 35 | 37 | 19/16 | 22/15 | 45.0 ± 11.1 | 43.1 ± 11.6 | Phospholipidated curcumin 250 mg | Placebo | 8 | Weight BMI HC WHR SBP DBP ALT AST ALP TG TC HDL-C LDL-C FBG |
|  | △Seyed Ali Hosseinian[35] | Iran | 2022 | 35 | 37 | 19/16 | 22/15 | 45.0 ± 11.1 | 43.1 ± 11.6 | Phytosomal curcuminoids 250 mg | Placebo | 8 | TNF-α |
|  | △Zahra Safari[36] | Iran | 2023 | 28 | 28 | 17/11 | 11/17 | 43.92 ± 8.74 | 50.35 ± 9.44 | Phytosomal curcumin 250 mg | Placebo | 12 | Weight BMI WC ALT AST TG TC HDL-C LDL-C FBG |
|  | △Roya Navekar[37] | Iran | 2017 | 21 | 21 | 11/10 | 8/13 | 42.09±7.23 | 40.38±9.26 | Turmeric powder 3,000 mg | Placebo | 12 | FBG Insulin HOMA-IR |
|  | △Aida Ghaffari[38] | Iran | 2019 | 21 | 21 | 11/10 | 8/13 | 42.5 ± 6.93 | 40.3 ± 9.26 | Turmeric powder 3,000 mg | Placebo | 12 | WC WHR ALT AST GGT ALP TG TC HDL -C LDL-C |
|  | △Maryam jarhahzadeh[39] | Iran | 2021 | 32 | 32 | 19/13 | 19/13 | 44.12±8.35 | 38.56±10.43 | Turmeric 2,000 mg | Placebo | 8 | ALT AST GGT TG TC HDL-C LDL-C FBG |
|  | △Ali Kalhori[40] | Iran | 2022 | 21 | 21 | 11/10 | 8/13 | 42.09±7.23 | 40.38±9.26 | Turmeric powder 3,000 mg | Placebo | 12 | Weight BMI SBP DBP |
| Resveratrol | △Veronique S. Chachay[41] | Australia | 2014 | 10 | 10 | 10/0 | 10/0 | 48.8±12.2 | 47.5±11.2 | Resveratrol 3,000 mg | Placebo | 8 | Weight BMI SBP DBP ALT AST TG TC HDL-C LDL-C FBG Insulin HOMA-IR TNF -α |
|  | △Shihui Chen[42] | China | 2015 | 30 | 30 | 22/8 | 20/10 | 45.2 ± 10.0 | 43.5 ± 11.0 | Resveratrol 600 mg | Placebo | 12 | Weight BMI WC HC WHR SBP DBP ALT AST GGT TG TC HDL-C LDL-C FBG Insulin HOMA-IR TNF -α |
|  | △Sara Heebøll[43] | Danmark | 2016 | 13 | 13 | 9/4 | 8/5 | 43.2(22-67)* | 43.5(21-69)* | Resveratrol 1,500 mg | Placebo | 24 | Weight BMI WHR SBP DBP ALT AST GGT ALP TG HDL-C LDL-C FBG Insulin TNF-α |
|  | △L. Farzin[44] | Iran | 2020 | 25 | 25 | 18/7 | 17/8 | 39.78±8.09 | 38.71±5.76 | Resveratrol 600 mg | Placebo | 12 | Weight BMI WC HC WHR SBP DBP ALT AST GGT ALP |
|  | △Forouzan Faghihzadeh[45] | Iran | 2014 | 25 | 25 | 18/7 | 17/8 | 44.04±10.10 | 46.28±9.52 | Pure trans-resveratrol 500 mg | Placebo | 12 | TNF-α |
|  | △Forouzan Faghihzadeh[46] | Iran | 2015 | 25 | 25 | 18/7 | 17/8 | 44.04±10.10 | 46.28±9.52 | Pure trans-resveratrol 500 mg | Placebo | 12 | Weight BMI WC HC WHR SBP DBP ALT AST GGT TG TC HDL-C LDL-C FBG Insulin HOMA-IR |
|  | △Somayyeh Asghari[47] | Iran | 2018 | 30 | 30 | 21/9 | 19/11 | 39.8±7.74 | 39.27±5.51 | Pure trans-resveratrol 600 mg | Placebo | 12 | TG TC HDL -C LDL-C FBG Insulin HOMA-IR |
|  | △Somayyeh Asghari[48] | Iran | 2018 | 30 | 30 | 21/9 | 19/11 | 40.00(22-58)* | 38.50(30-48)* | Pure trans-resveratrol 600 mg | Placebo | 12 | ALT AST GGT ALP |
| Anthocyanin | △Pei-Wen Zhang[49] | China | 2015 | 37 | 37 | 19/18 | 20/17 | 44.9±7.5 | 46.9±7.7 | Purified anthocyanin 320 mg | Placebo | 12 | Weight BMI WC HC WHR SBP DBP ALT AST TG TC HDL-C LDL-C FBG Insulin HOMA-IR |
|  | △Fatemeh Izadi[50] | Iran | 2021 | 30 | 31 | 17/13 | 19/12 | 43.3 ± 10.2 | 42.8 ± 10.6 | Sour tea powder capsules 450 mg | Placebo | 8 | Weight BMI WC SBP DBP ALT AST TG TC HDL-C LDL-C |
|  | △Zohreh Sadat Sangsefidi[51] | Iran | 2021 | 25 | 25 | 12/13 | 11/14 | 41.48 ± 9.53 | 42.68 ± 9.96 | *Cornus mas L.* fruit extract 20 ml | Placebo | 12 | ALT AST |
|  | ＊Hoda Mojiri-Forushani[52] | Iran | 2022 | 45 | 45 | 26/19 | 26/19 | 37.71 ± 9.39 | 36.04 ± 9.40 | Grape seed extract capsules 400 mg | Placebo | 8 | ALT AST TG TC HDL-C LDL-C FBG |
|  | △Faezeh Yarhosseini[53] | Iran | 2023 | 25 | 25 | 12/13 | 11/14 | 41.4 ± 9.5 | 42.6 ± 9.9 | *Cornus mas L.* fruit extract 20 ml | Placebo | 12 | Weight WC HC WHR SBP DBP |
| Silymarin | △Seyed Jalal Hashemi[54] | Iran | 2009 | 50 | 50 | 28/22 | 29/21 | 39.28±11.117 | 39.0±10.70 | Silymarin active extract 280 mg | Placebo | 24 | BMI ALT AST TG TC HDL-C LDL-C FBG |
|  | △Mohesn Masoodi[55] | Iran | 2013 | 50 | 50 | 31/19 | 31/19 | 48.42±6.75 | 48.32±5.45 | Silymarin 280 mg | Placebo | 12 | BMI ALT AST |
|  | △Hassan Solhi[56] | Iran | 2014 | 33 | 31 | 19/14 | 19/12 | 43.6±8.3 | 39.36±10.5 | Silymarin 210 mg | Placebo | 8 | ALT AST |
|  | △Chan Wah Kheong[57] | Malaysia | 2017 | 49 | 50 | 24/25 | 22/28 | 49.6±12.7 | 50.1±10.2 | Silymarin 2,100 mg | Placebo | 48 | Weight ALT AST GGT TG TC HDL-C LDL-C FBG HOMA-IR |
|  | △Amir Anushiravani[58] | Iran | 2019 | 30 | 30 | 73/77 | | 47.0 ± 9.1 (range: 18–65) | | Silymarin 140 mg | Placebo | 12 | BMI WC ALT AST TG TC HDL-C LDL-C FBG |
| Catechin | △RYUICHIRO SAKATA[59] | Japan | 2013 | 7 | 5 | 7 /10 | | 47.1±17.2 | 54.2＋8.1 | Catechin 1,080 mg/ 700 ml | Placebo | 12 | Weight BMI ALT |
|  | △Ali Pezeshki[60] | Iran | 2016 | 35 | 36 | 16/19 | 16/20 | aged 20–50 years | | Green tea extract 500 mg | Placebo | 12 | Weight BMI ALT AST ALP |
|  | △Mazhar Hussain[61] | Pakistan | 2017 | 40 | 40 | 26/14 | 28/12 | 25±18 | 28±15 | Green tea extract 1,000 mg | Placebo | 12 | Weight BMI ALT AST TG TC HDL-C LDL-C HOMA-IR |
|  | △Seyed Mohammad Tabatabaee[62] | Iran | 2017 | 21 | 24 | 15/18 | 12/22 | age≥18 years | | Green tea tablets 550 mg | Placebo | 12 | Weight BMI ALT AST TG TC HDL-C LDL-C FBG Insulin HOMA-IR |
| Chlorogenic acid | △Hedayat Allah Shahmohammadi[63] | Iran | 2017 | 22 | 22 | 11/11 | 11/11 | 41.36 ± 7.69 | 44.50 ± 5.24 | Green coffee bean extract 1,000 mg | Placebo | 8 | Weight BMI WC HC WHR ALT AST ALP TG TC HDL-C LDL-C FBG Insulin HOMA-IR TNF-α |
|  | △Samaneh Hosseinabadi[64] | Iran | 2019 | 21 | 23 | 11/10 | 12/11 | 41.14 ± 7.87 | 41.13 ± 8.47 | Green coffee extract capsules 400 mg | Placebo | 8 | SBP DBP ALT AST |
|  | △Samaneh Hosseinabadi[65] | Iran | 2020 | 21 | 23 | 11/10 | 12/11 | 41.14 ± 7.87 | 41.13 ± 8.47 | Green coffee extract capsules 400 mg | Placebo | 8 | Weight BMI TG TC HDL-C LDL-C |
|  | △Samaneh Hosseinabadi[66] | Iran | 2020 | 21 | 23 | 11/10 | 12/11 | 41.14 ± 7.87 | 41.13 ± 8.47 | Green coffee extract capsules 400 mg | Placebo | 8 | WC WHR FBG Insulin HOMA-IR |
| Ellagic acid | △Reza Goodarzi[67] | Iran | 2021 | 22 | 22 | 13/9 | 11/11 | 47.41 ± 9.58 | 44.91 ± 9.41 | Dried pomegranate fruit extract tablets 450 mg | Placebo | 12 | Weight BMI WC SBP DBP TG TC HDL-C LDL-C FBG Insulin HOMA-IR |
|  | △Hanieh Barghchi[68] | Iran | 2023 | 39 | 37 | 18/21 | 19/18 | 42.8±7.2 | 43.3±10.1 | Pomegranate peel extract 1,500 mg | Placebo | 8 | Weight BMI WC SBP DBP TG TC HDL-C LDL-C FBG Insulin HOMA-IR |
| Genistin/Genistein | △Sasan Amanat[69] | Iran | 2018 | 41 | 37 | 30/11 | 31/10 | aged 18-69 years | | Genistein 250 mg | Placebo | 8 | Weight BMI WC WHR SBP DBP ALT AST TG TC HDL-C LDL-C FBG Insulin HOMA-IR TNF-α |
|  | △Asal Neshatbini Tehrani[70] | Iran | 2024 | 25 | 21 | 10/15 | 10/11 | Male：47.60±14.98 Female：51.93±11.15 | Male：46.0±14.10 Female：52.09±5.73 | Soy isoflavone 100 mg | Placebo | 12 | Weight BMI WC HC WHR ALT AST GGT |
| Naringenin | △Zahra Namkhah[71] | Iran | 2021 | 22 | 22 | 12/10 | 13/9 | 44.7 ± 10.7 | 47 ± 9 | Naringenin 200 mg | Placebo | 4 | Weight BMI WC ALT AST TG TC HDL-C LDL-C |
|  | △Fatemeh Naeini[72] | Iran | 2022 | 22 | 22 | 12/10 | 13/9 | 45±11 | 47±9 | Naringenin 200 mg | Placebo | 4 | SBP DBP |
| Dihydromyricetin | △Shihui Chen[73] | China | 2015 | 30 | 30 | 21/9 | 19/11 | 46.9 ± 8.44 | 43.2 ± 11.3 | Dihydromyricetin 600 mg | Placebo | 12 | Weight BMI WC HC WHR SBP DBP ALT AST GGT ALP TG TC HDL-C LDL-C FBG Insulin HOMA-IR TNF-α |
| Hesperidin | △Makan Cheraghpour[74] | Iran | 2019 | 25 | 24 | 10/13 | 12/12 | 47.32 ± 11.66 | 47.29 ± 13.76 | Hesperidin 1,000 mg | Placebo | 12 | ALT AST GGT TG TC LDL-C HDL-C FBG Insulin HOMA-IR TNF -α |
| Quercetin | △Mahboobe Hosseinikia[75] | Iran | 2020 | 39 | 39 | 15/24 | 13/26 | 43.4±11.1 | 45.9±9.2 | Quercetin 500 mg | Placebo | 12 | BMI WHR ALT AST GGT TG TC HDL-C LDL-C TNF-α |
| ①Silymarin ②Gallic acid and Chlorogenic acid | △Vamsi Krishna Pothula Rajendra[76] | India | 2022 | ①29 ②29 | 30 | ①14/16 ②16/14 | 16/14 | aged 25–60 years | | ①Silymarin 320 mg ②Terminalia chebula fruit and Sphaeranthus indicus flower head extracts 300 mg | Placebo | 12 | ALT AST GGT ALP TG TC HDL-C LDL-C HOMA-IR |

Note：NA: Not Applicable；# represents baseline data from original studies；*denotes (median(range))；△ denotes no conflict of interest; ＊denotes that conflict of interest may exist.
