# Supplementary material for: Efficacy of dietary polyphenol supplement in patients with non-alcoholic fatty liver disease: a network meta-analysis
Source: Front Nutr. 2025 May 9;12:1582861. doi: 10.3389/fnut.2025.1582861 (PMC12100629; doi:10.3389/fnut.2025.1582861)
Supplement: Supplementary file 3 [file Data_Sheet_1.zip › Supplementary Material 3.docx]

# Supplementary Material 3: Network diagrams, SUCRA plots, funnel plots, and league tables for other outcome measures

## **Figure 1: Network diagrams for other outcome measures.**

### (A) Weight


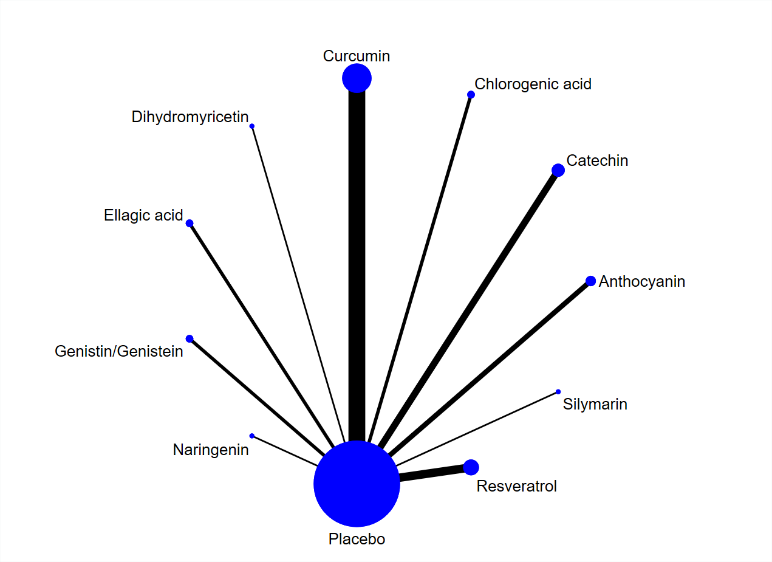


### (B) WC


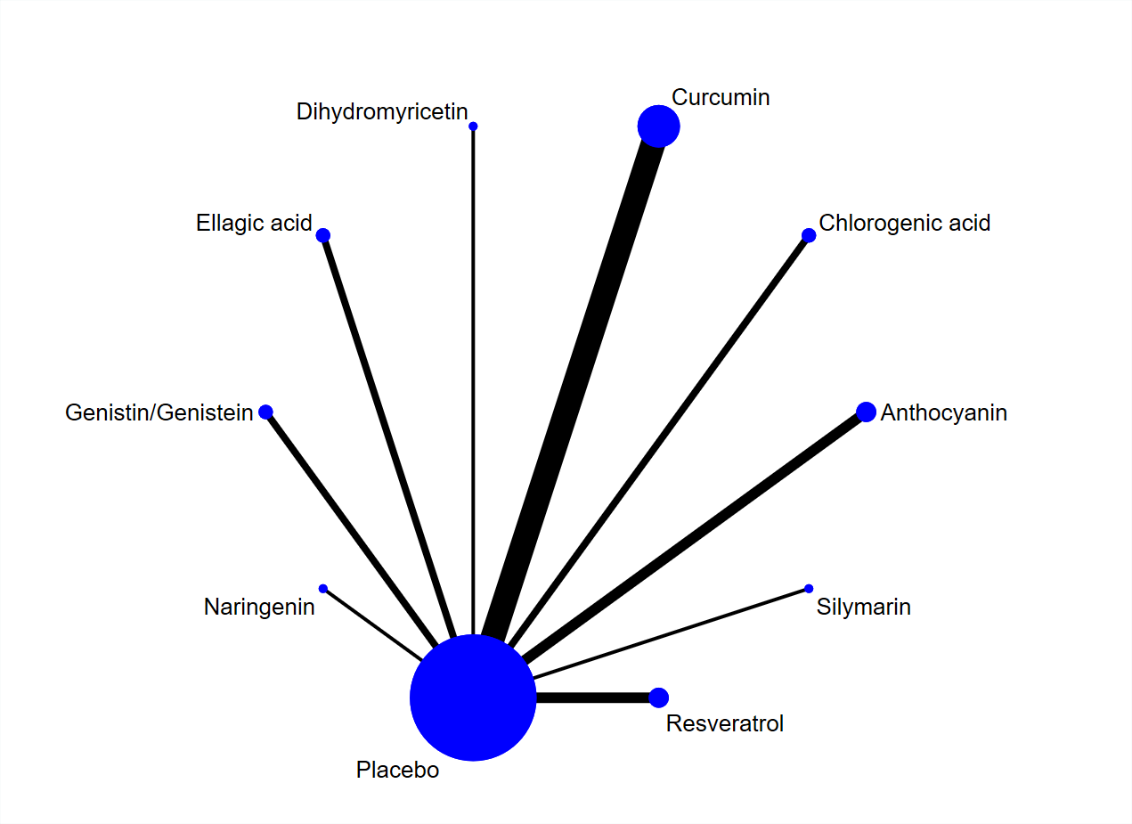


### (C) HC


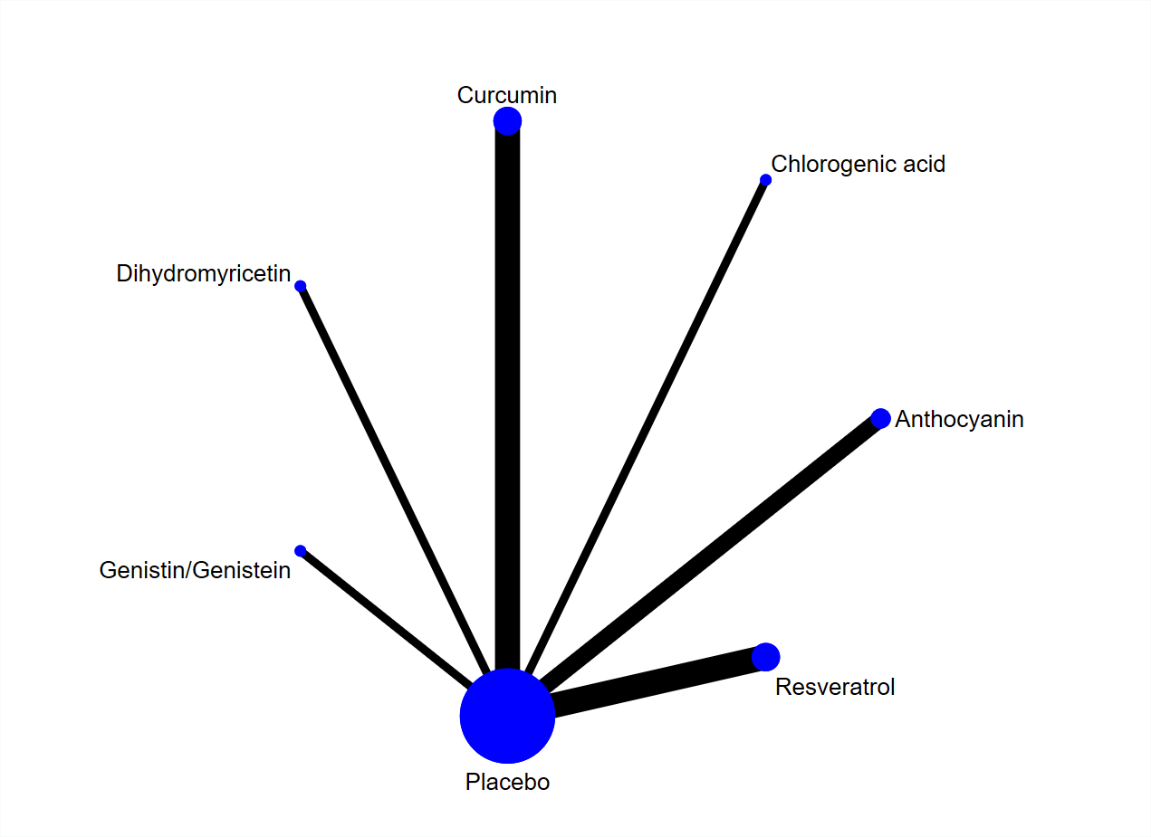


### (D) WHR


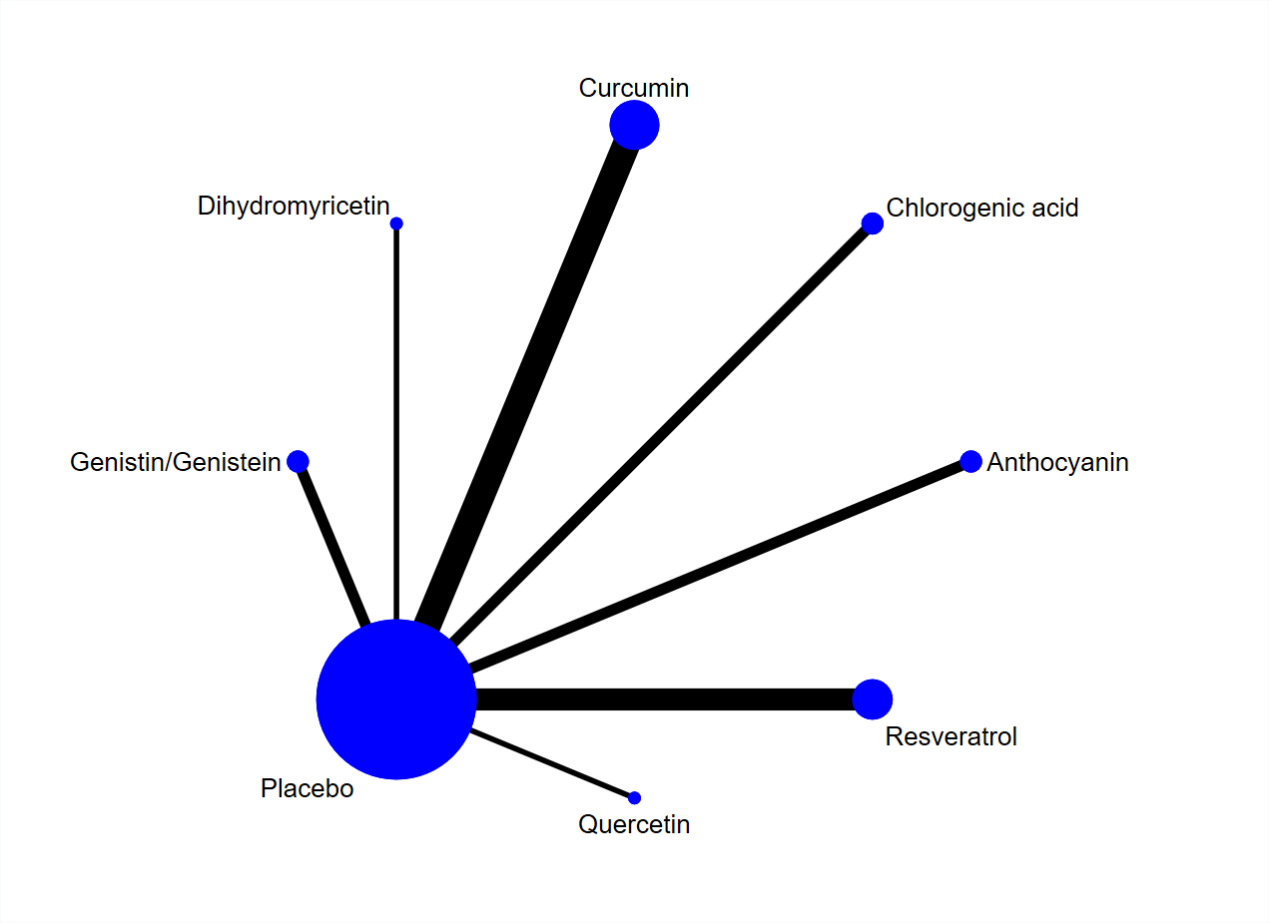


### (E) SBP


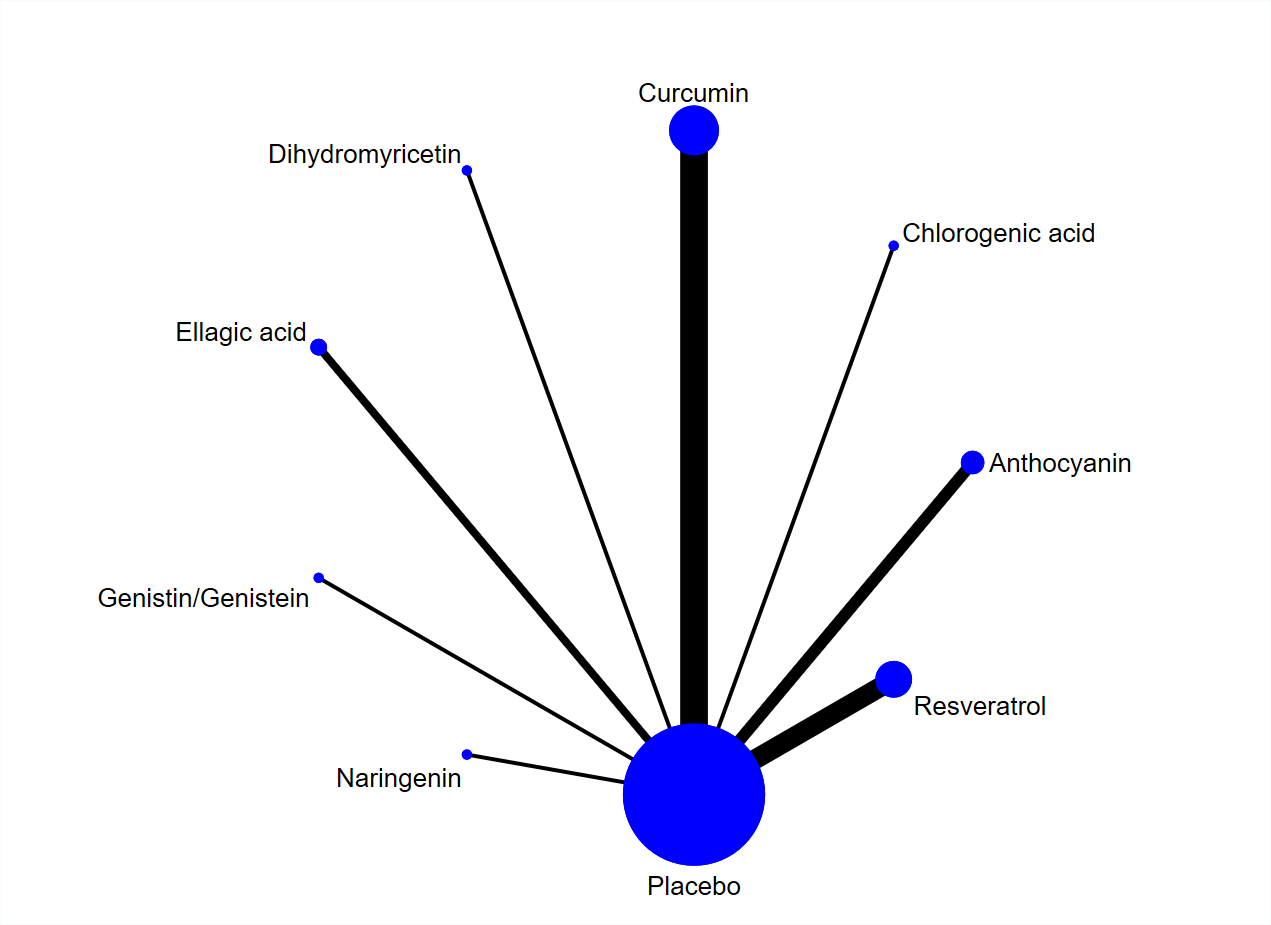


### (F) DBP


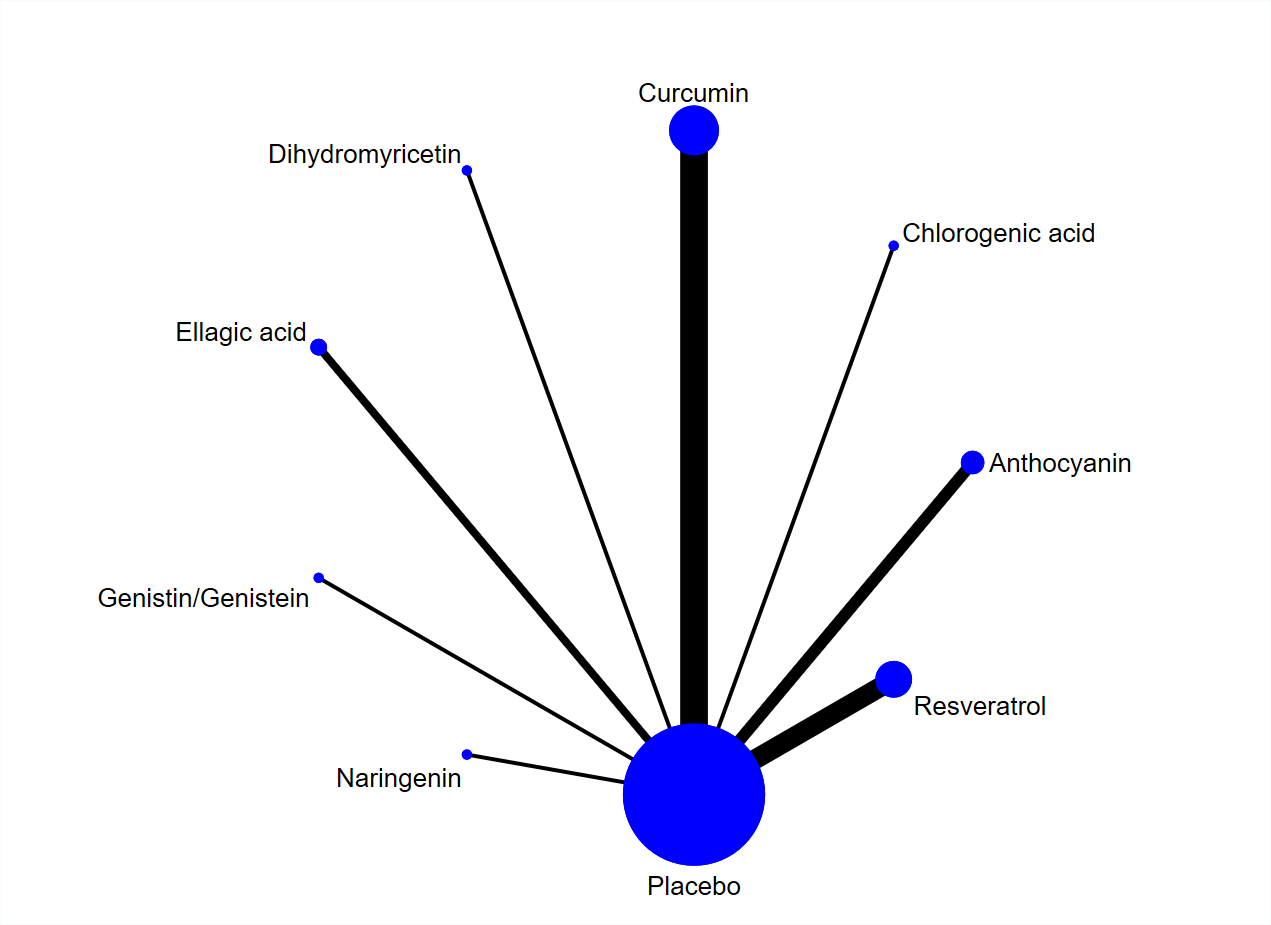


### (G) ALP


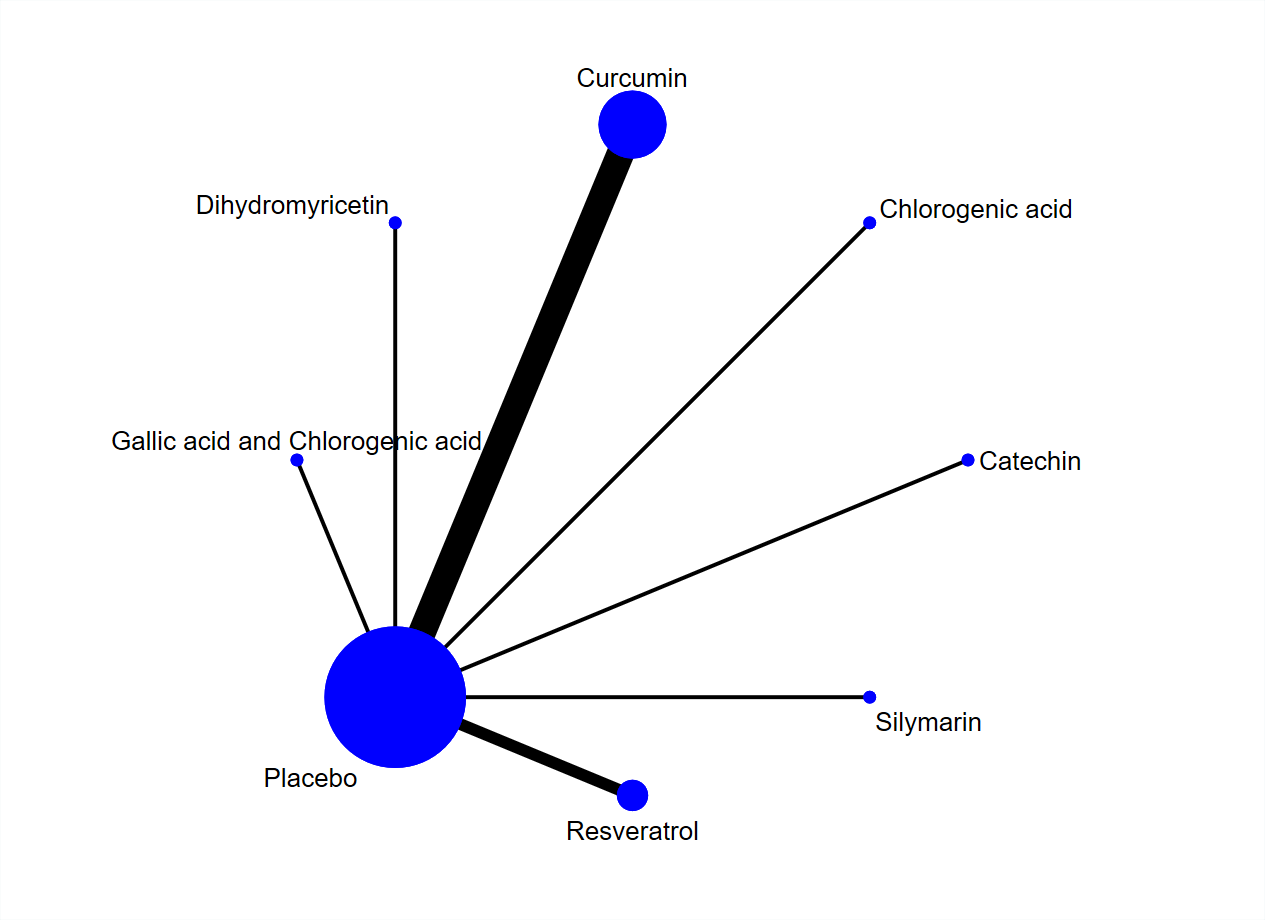


### (H) GGT


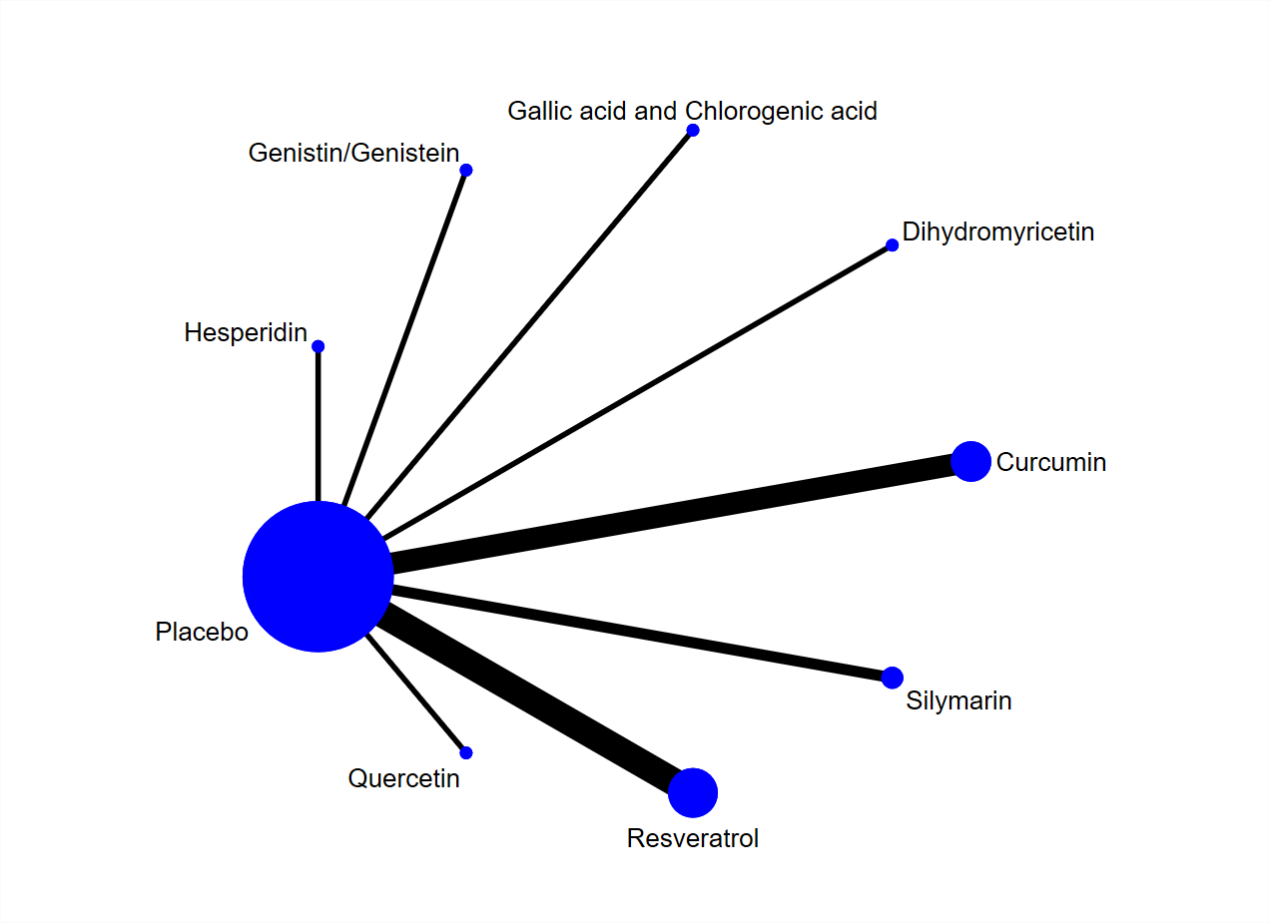


### (I) FBG


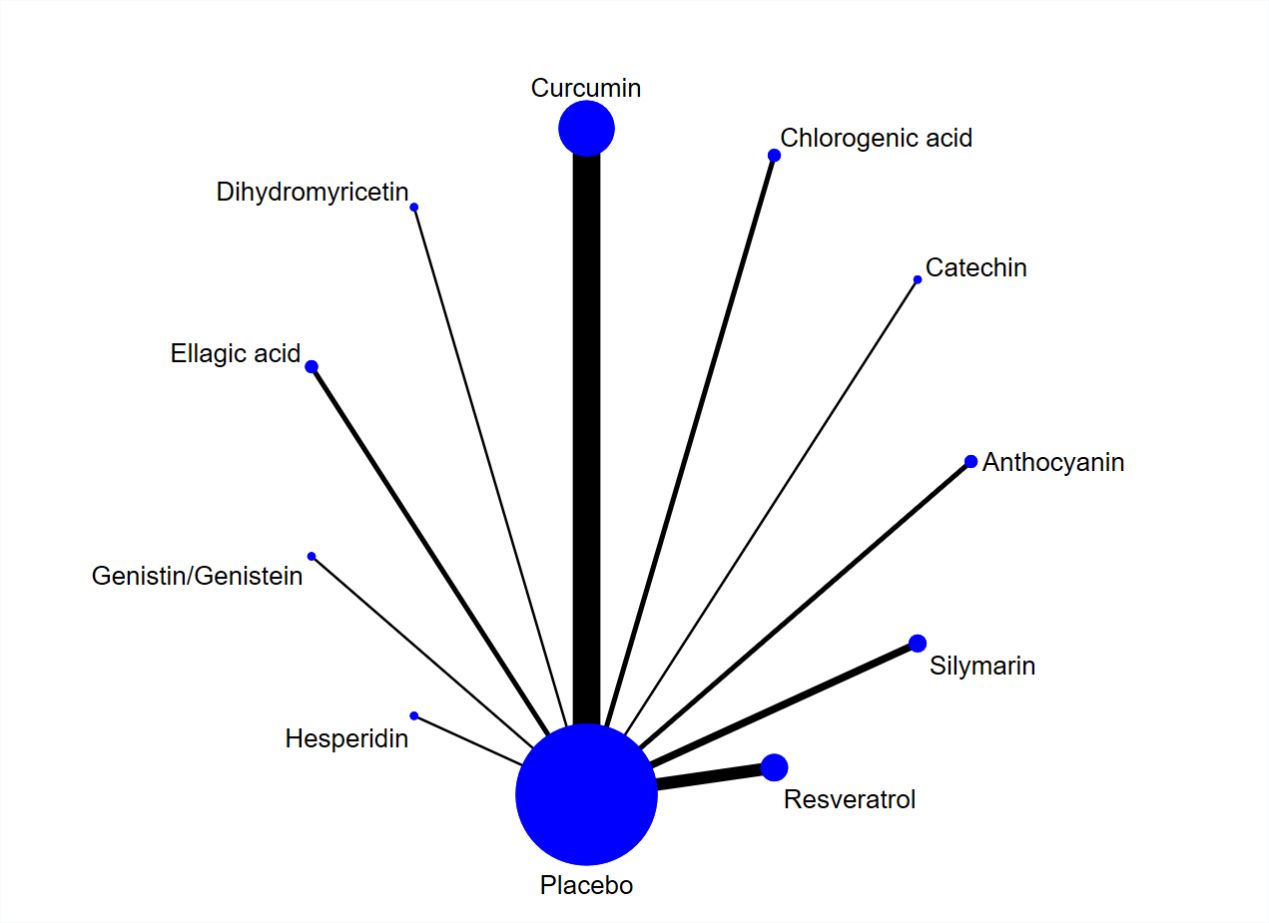


### (J) Insulin


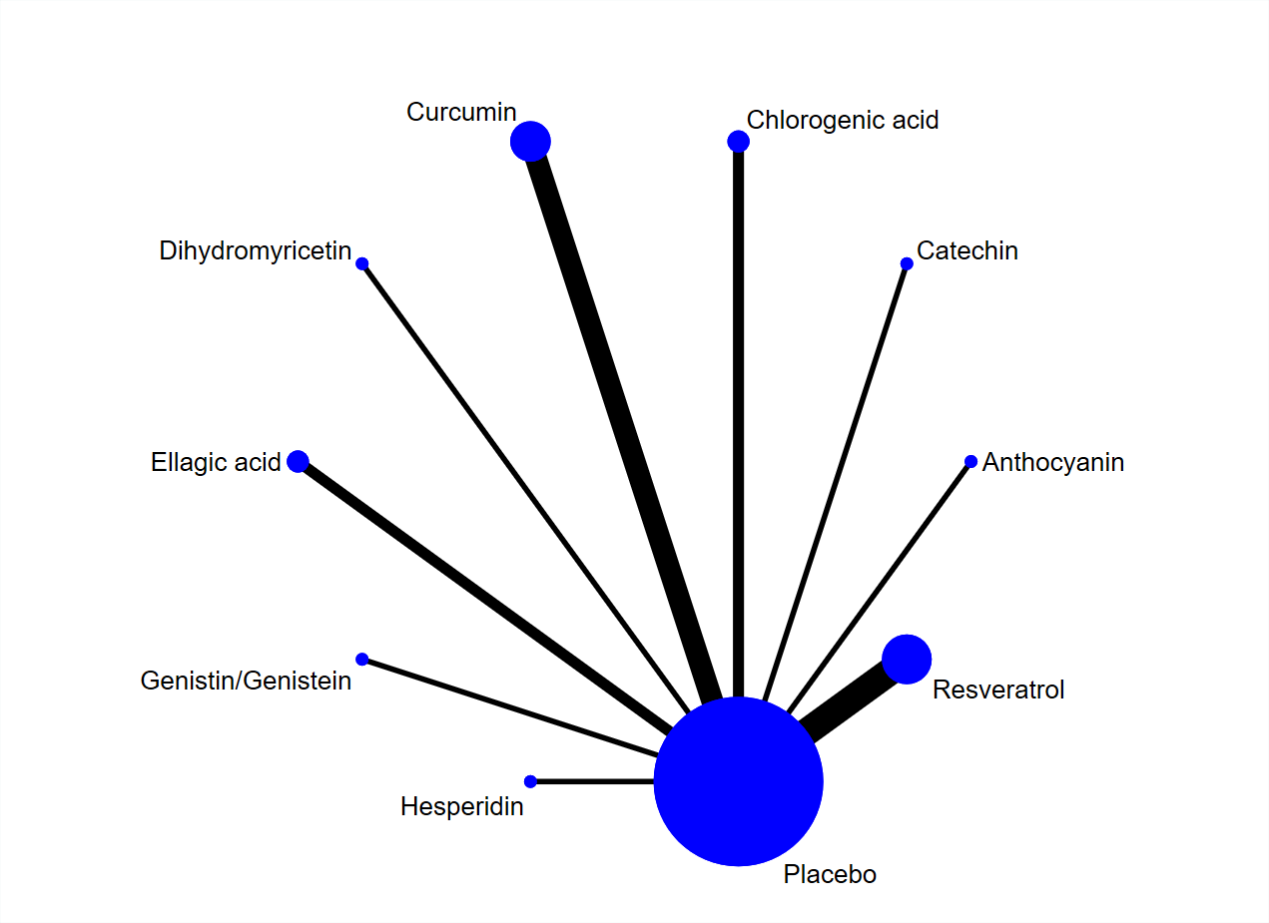


### (K) HOMA-IR


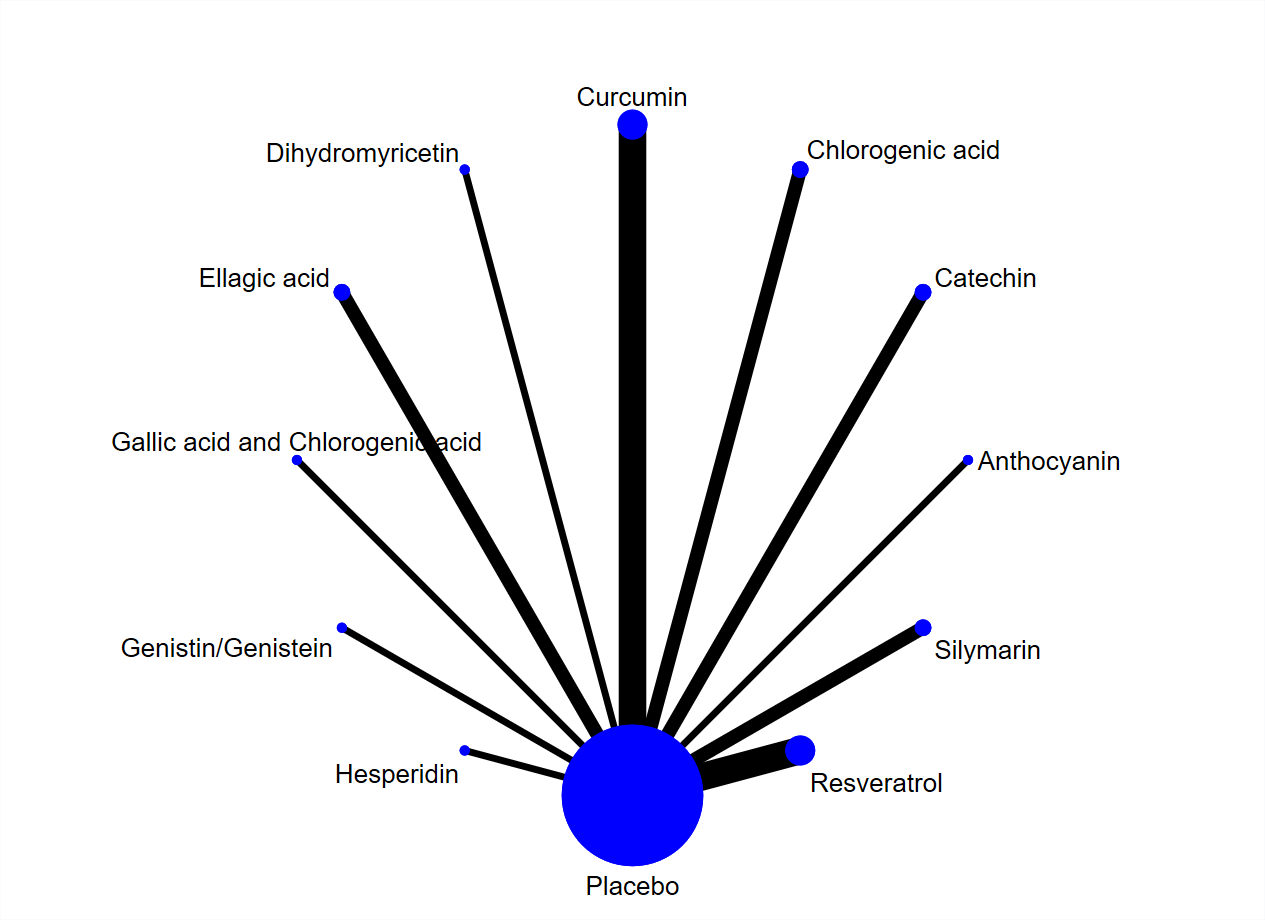


## **Figure 2: SUCRA plots for other outcome measures**

### (A) Weight


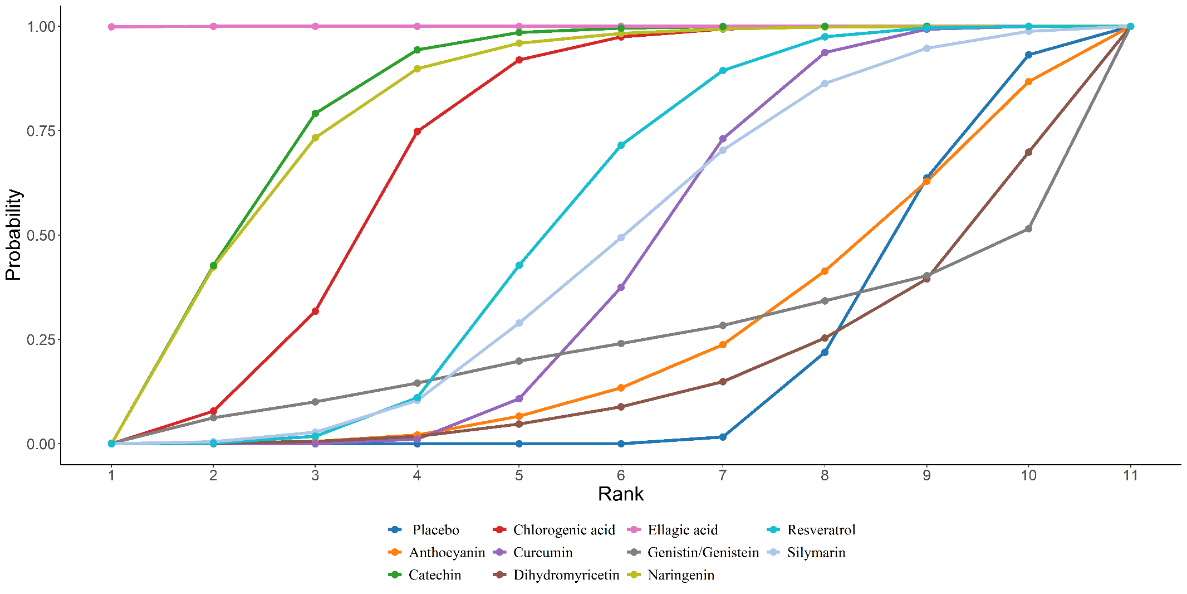


### (B) WC


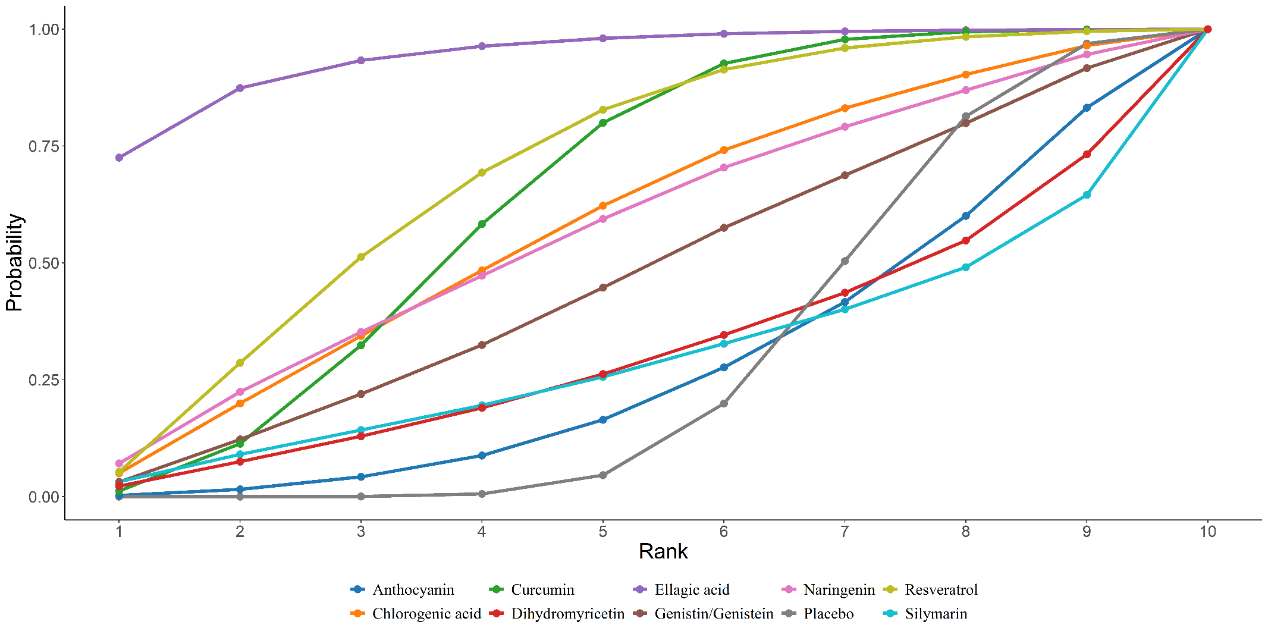


### (C) HC


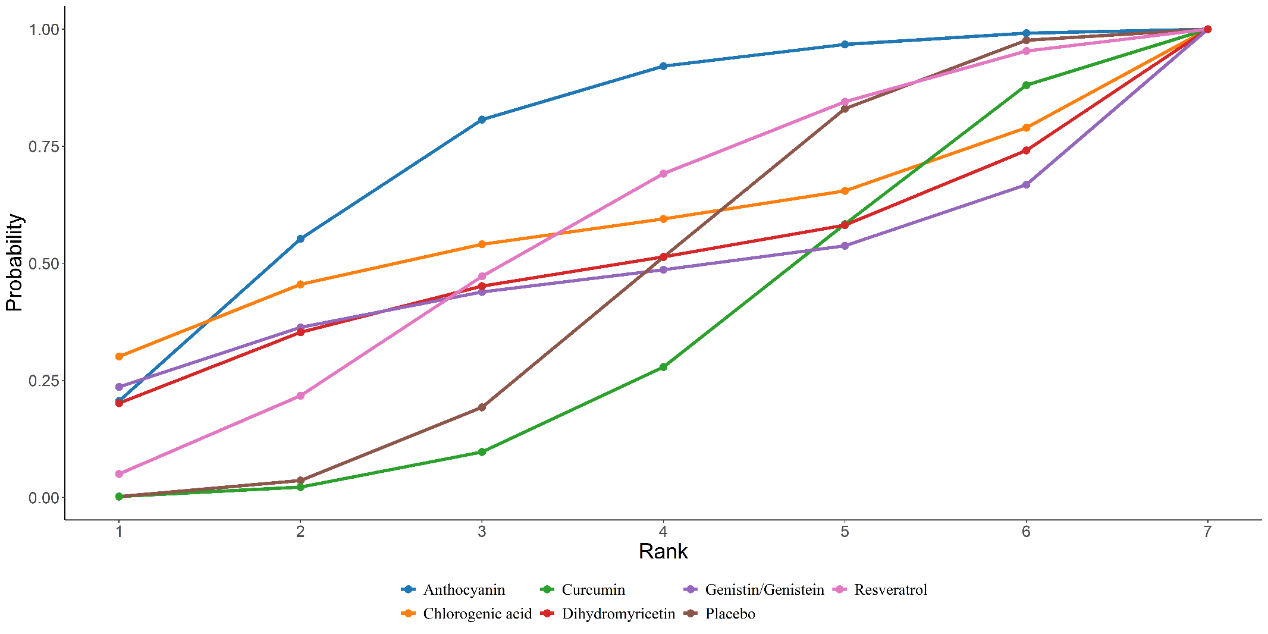


### (D) WHR


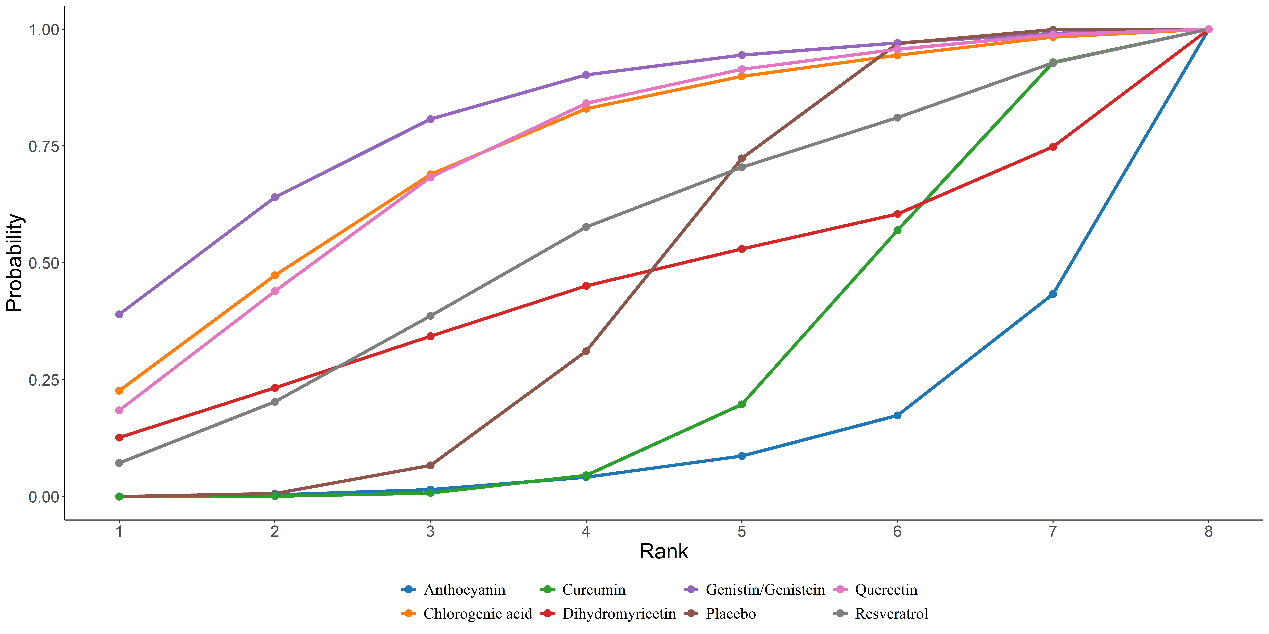


### (E) SBP


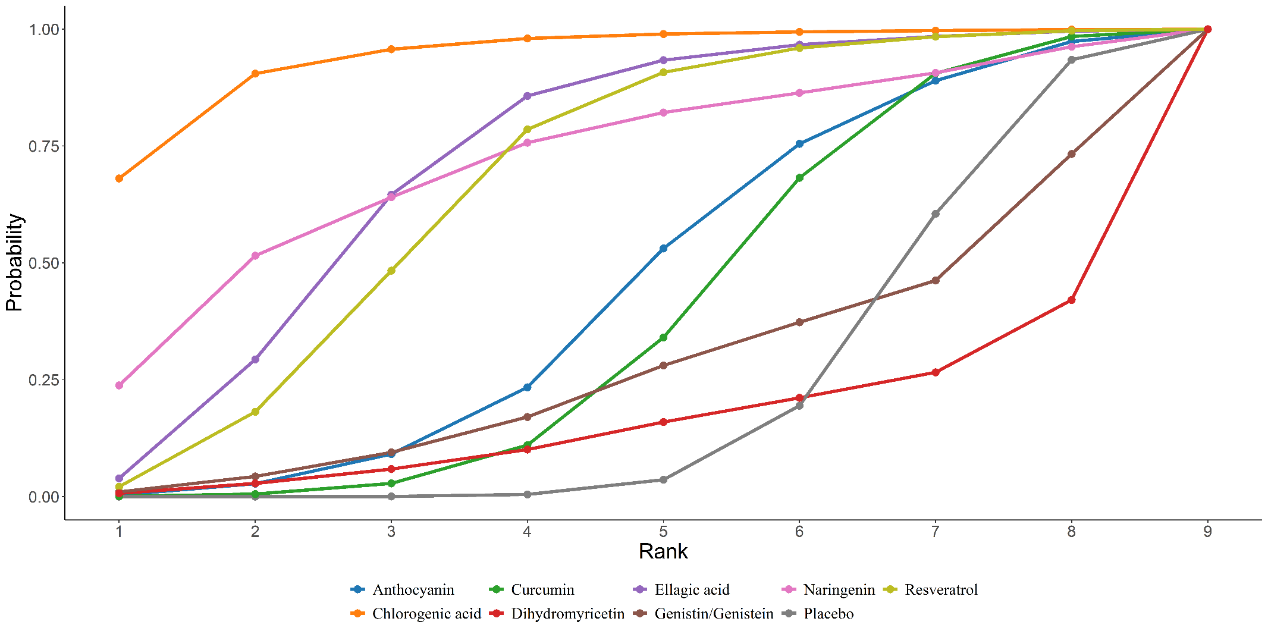


### (F) DBP


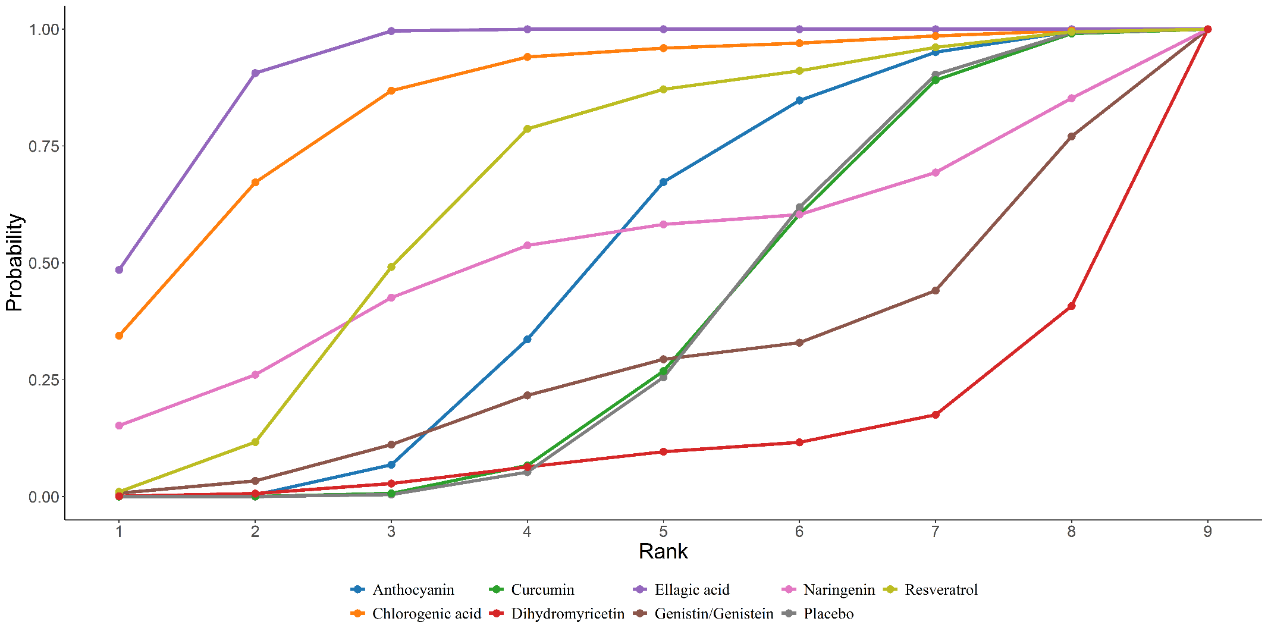


### (G) ALP


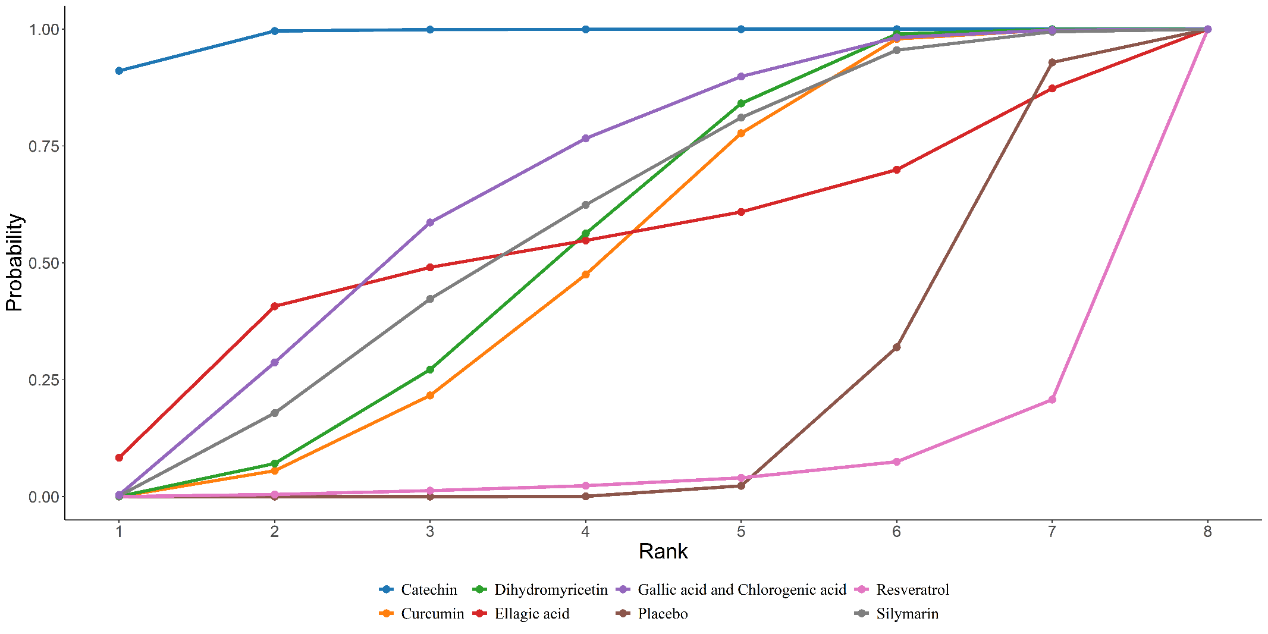


### (H) GGT


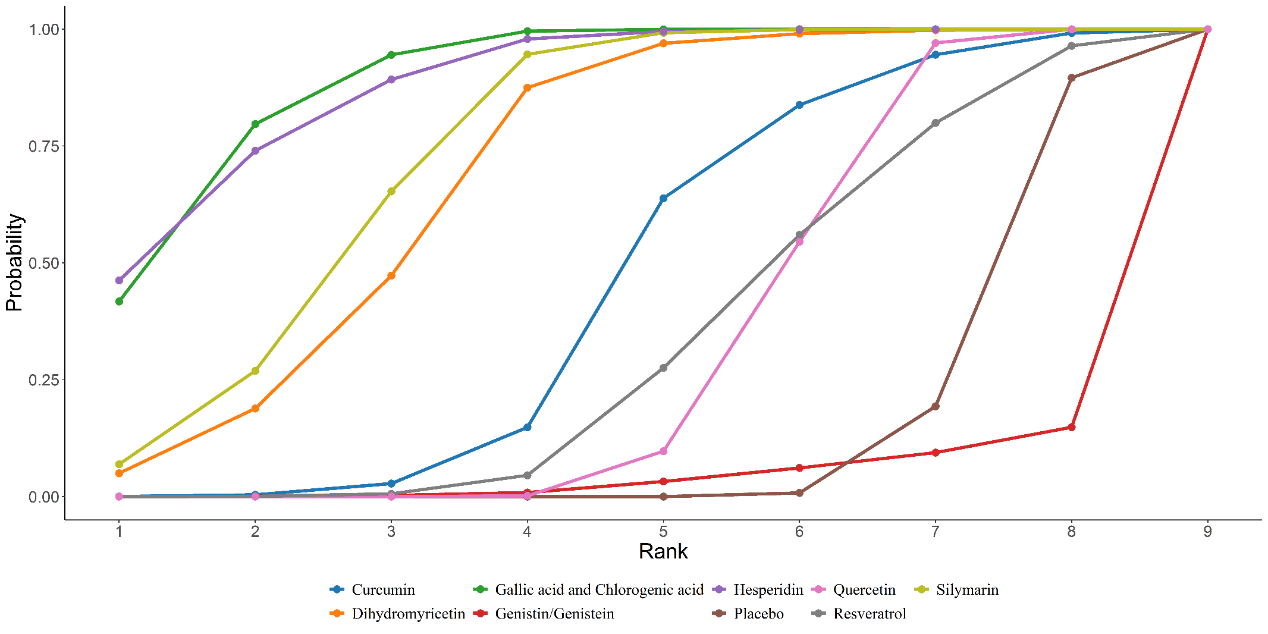


### (I) FBG


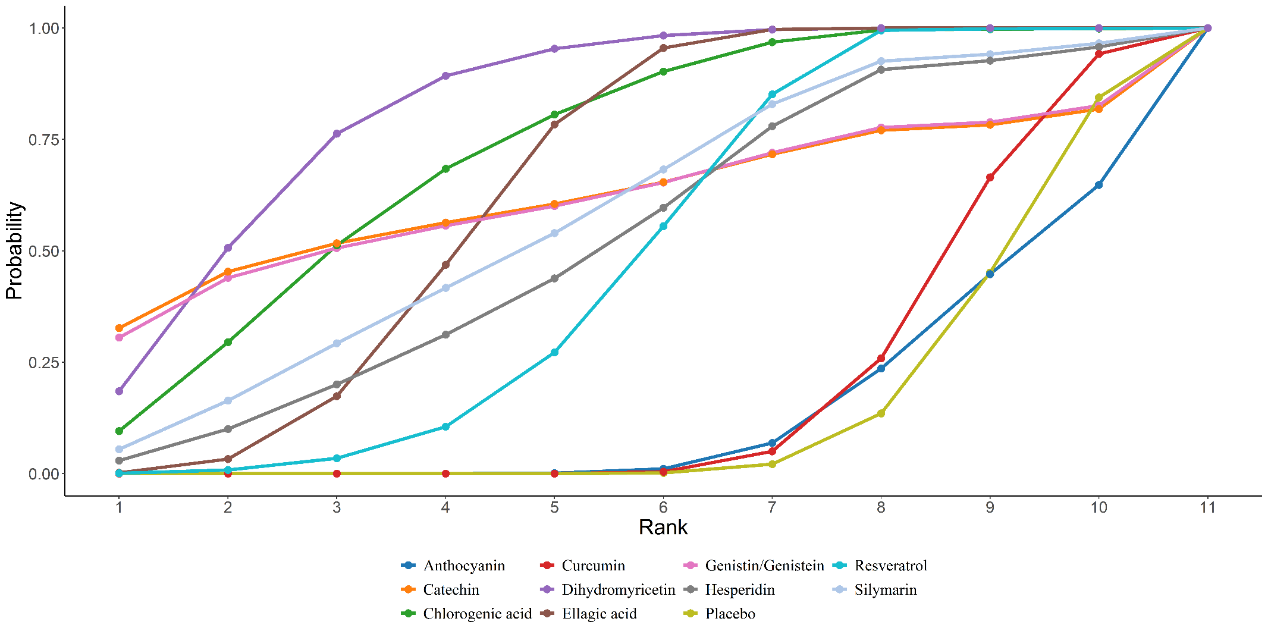


### (J) Insulin


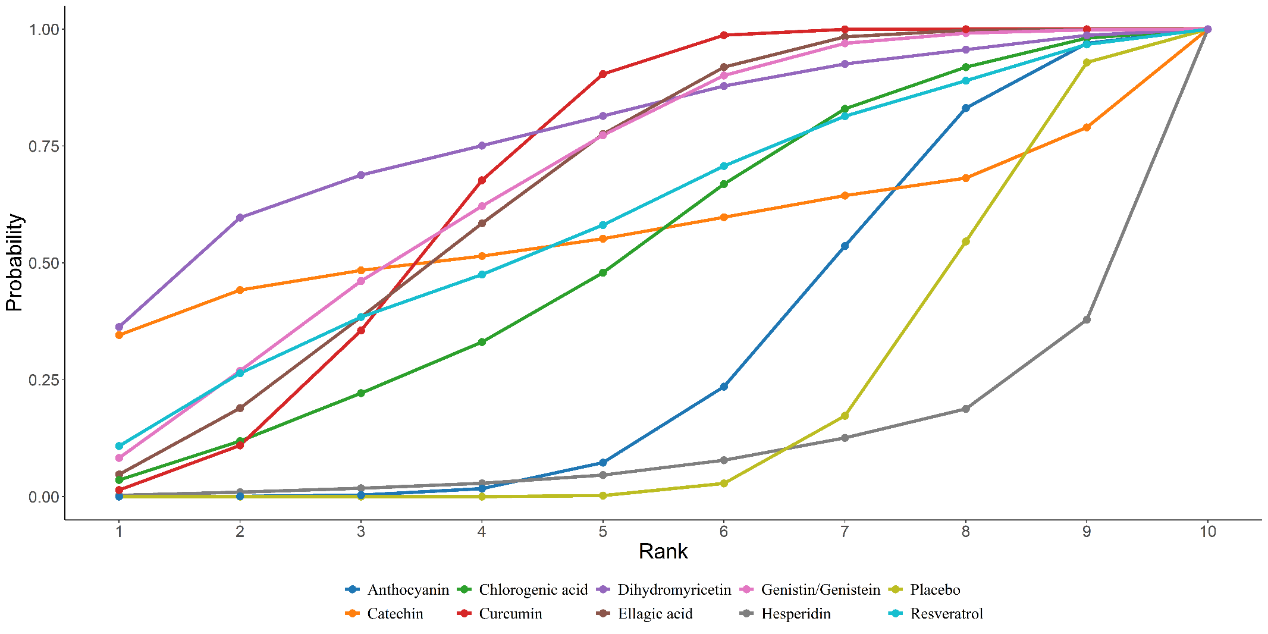


### (K) HOMA-IR


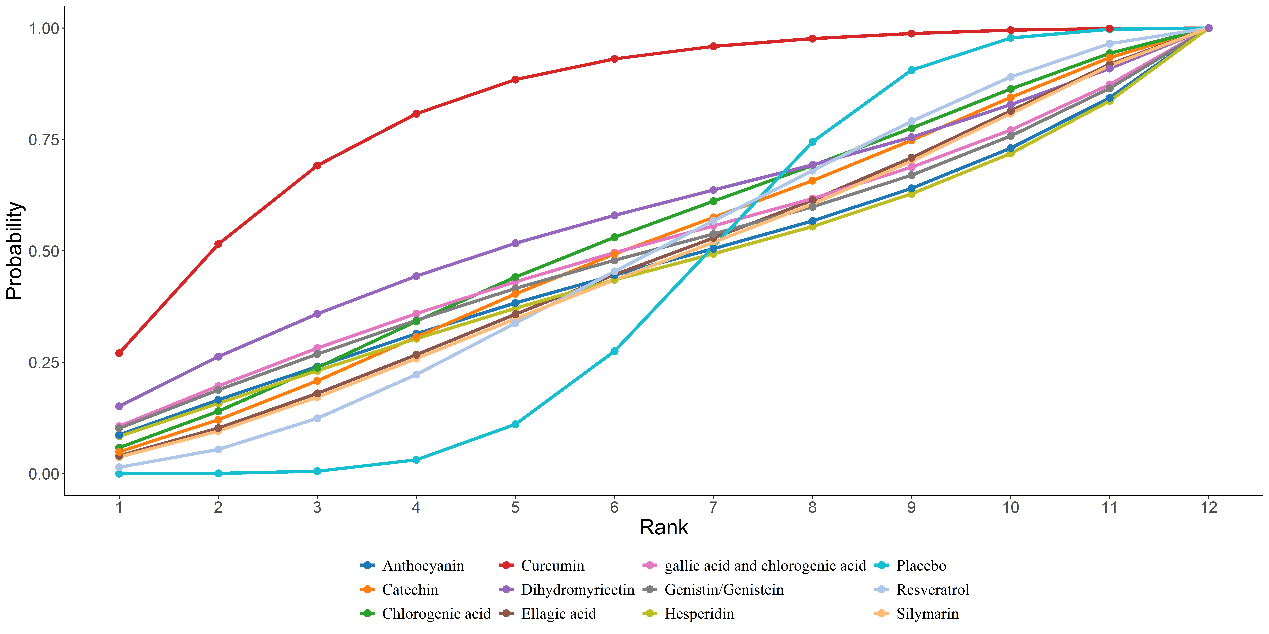


## **Figure 3: Funnel plots for other outcome measures**

### (A) Weight


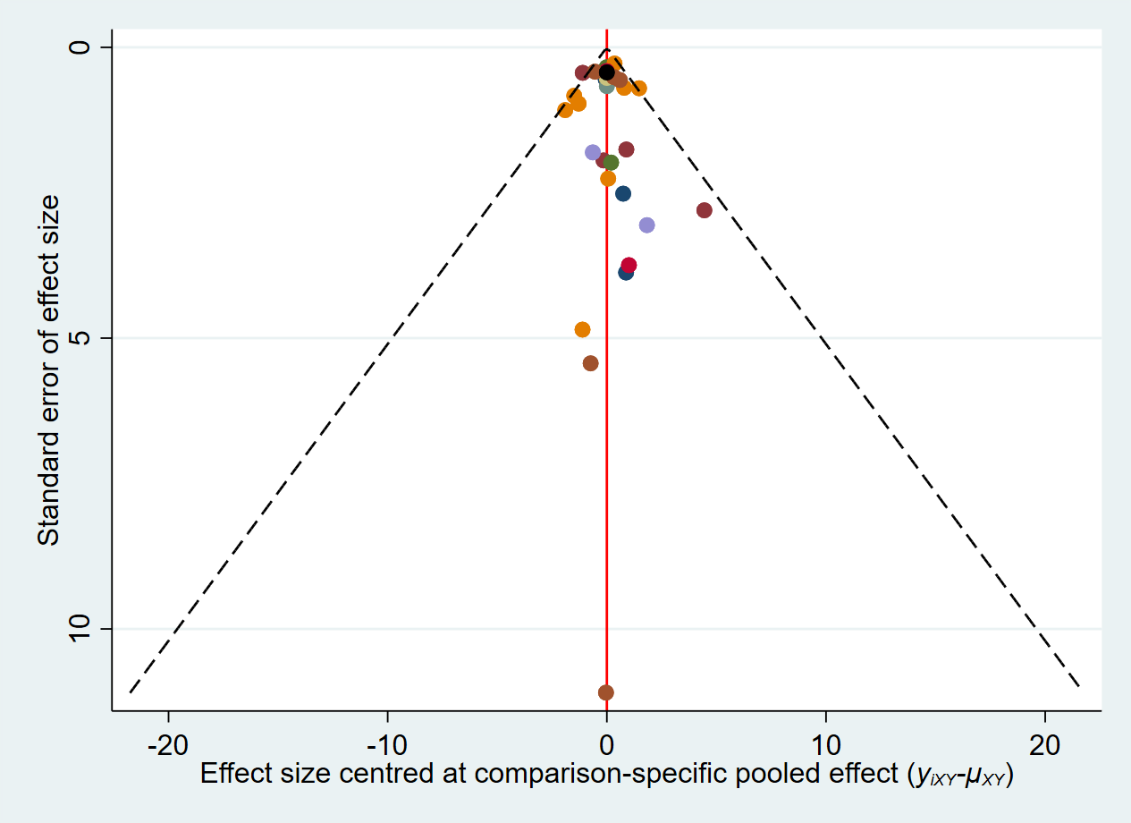


### (B) WC


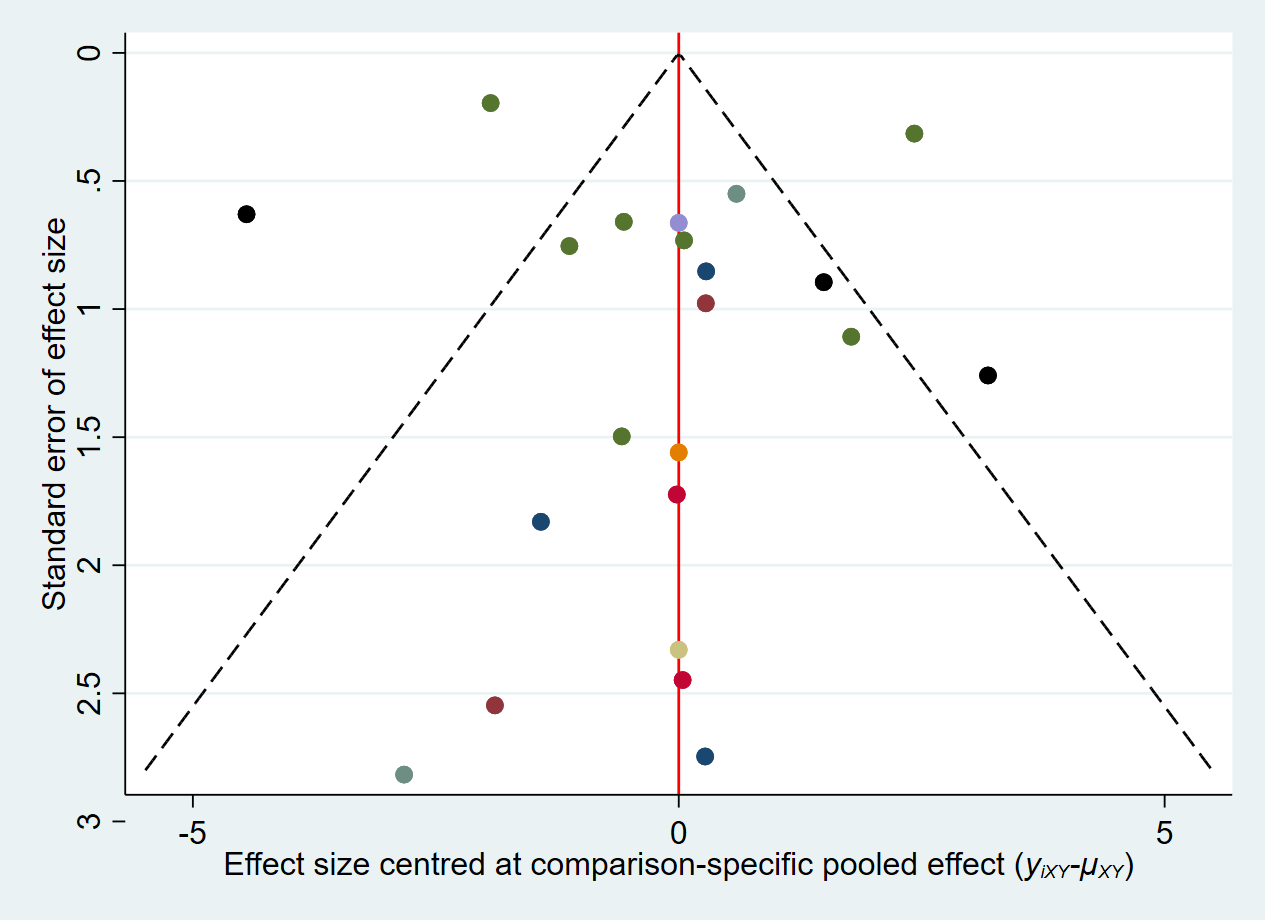


### (C) HC


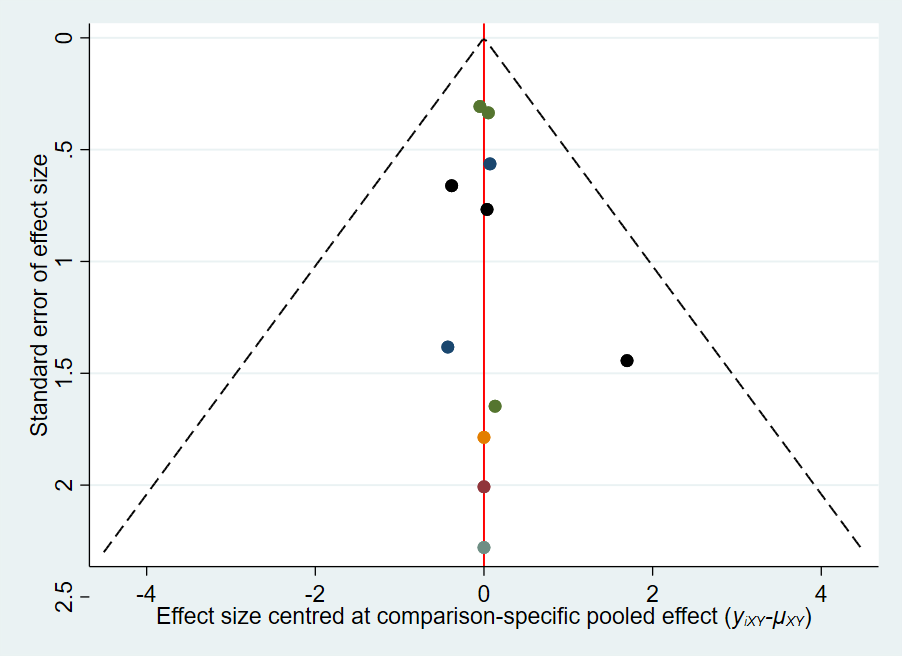


### (D) WHR


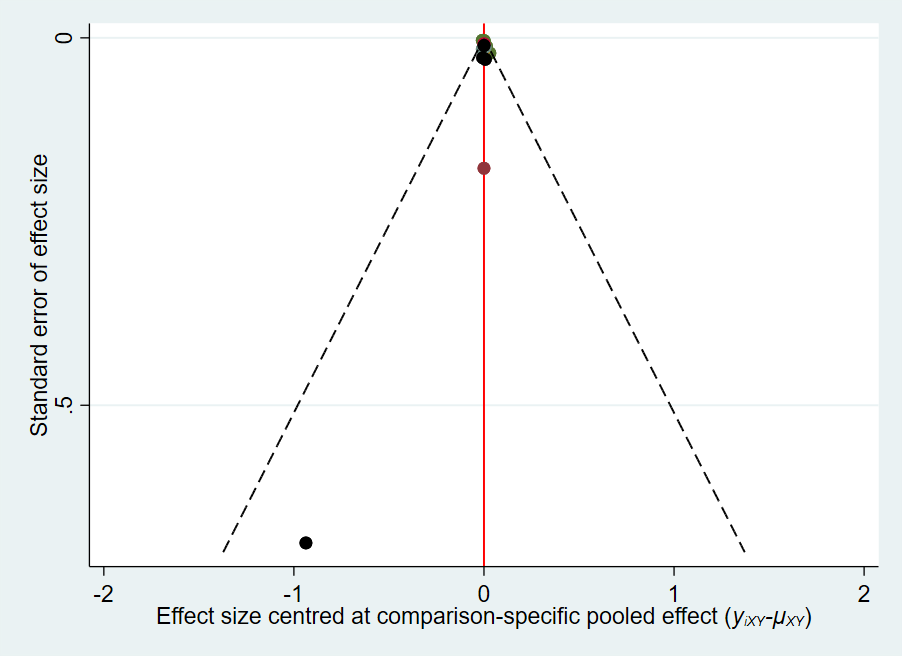


### (E) SBP


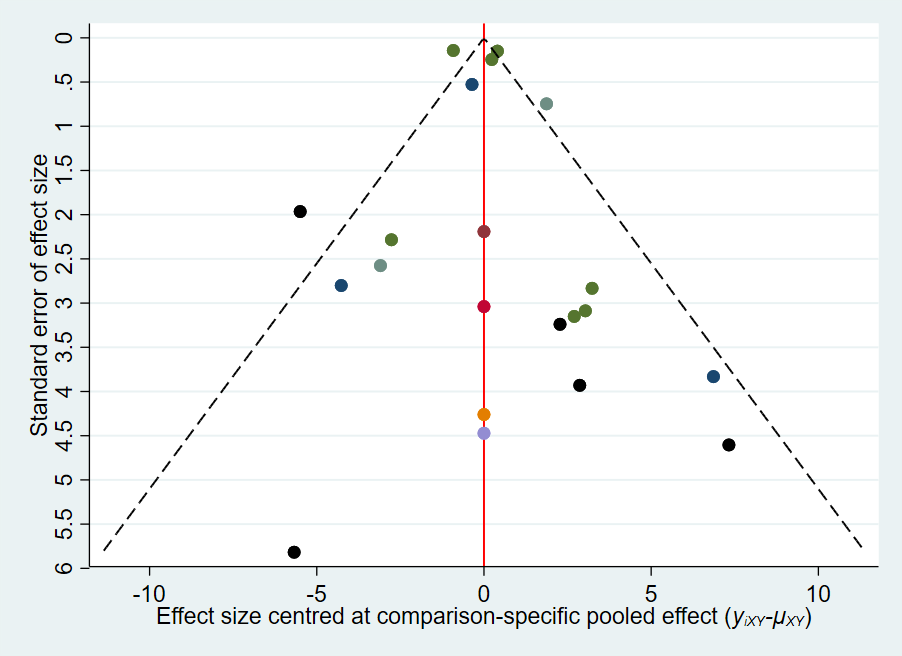


### (F) DBP


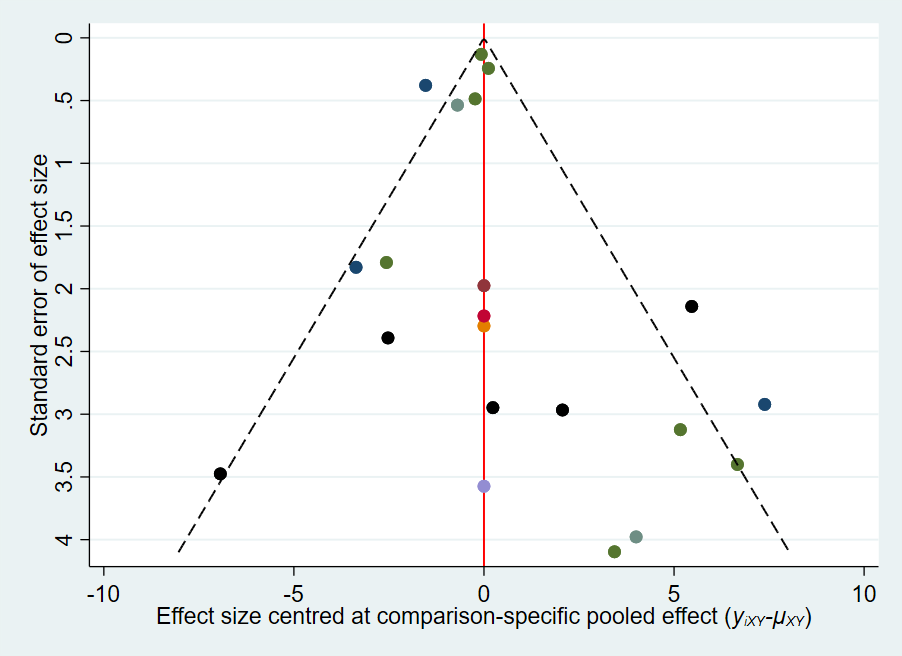


### (G) ALP


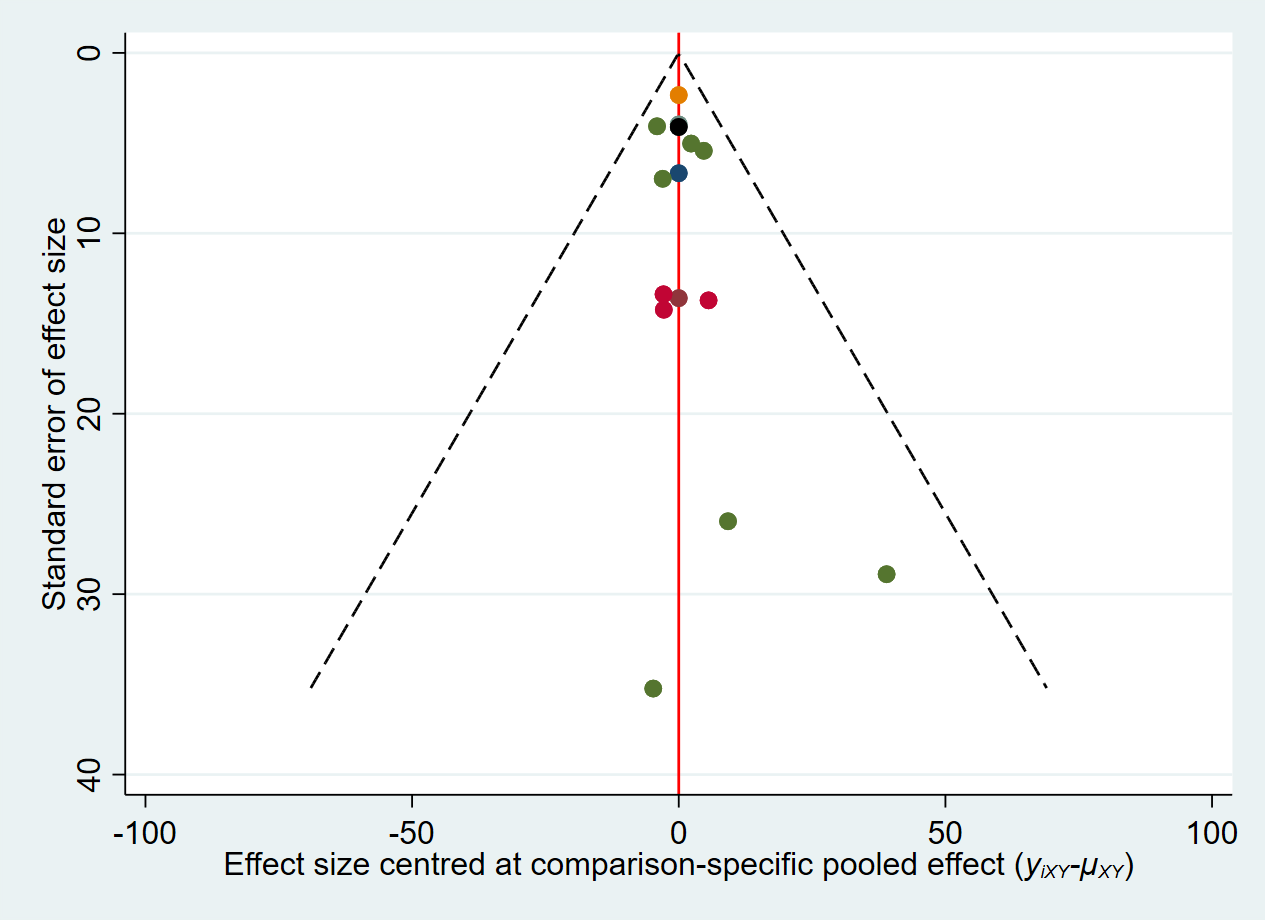


### (H) GGT


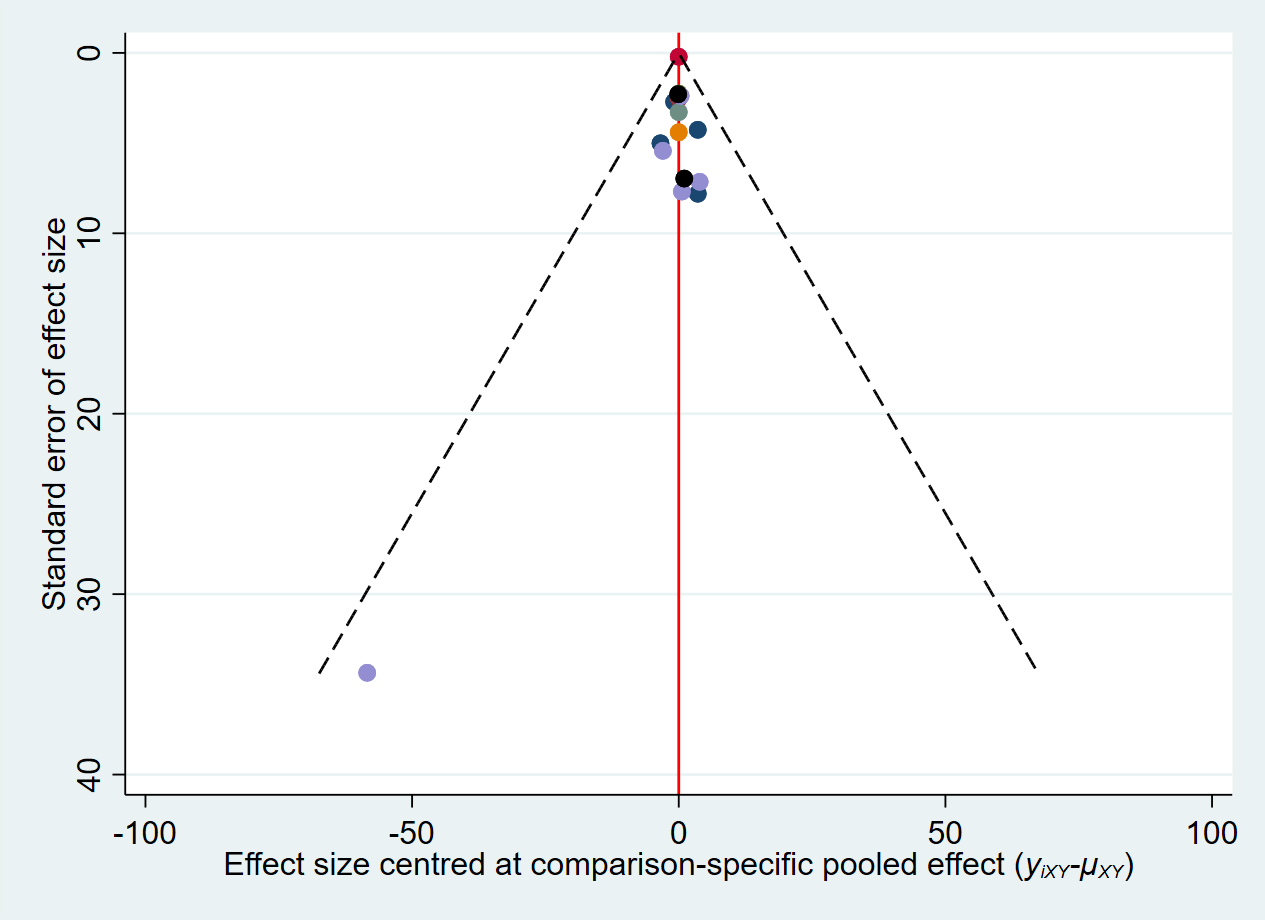


### (I) FBG


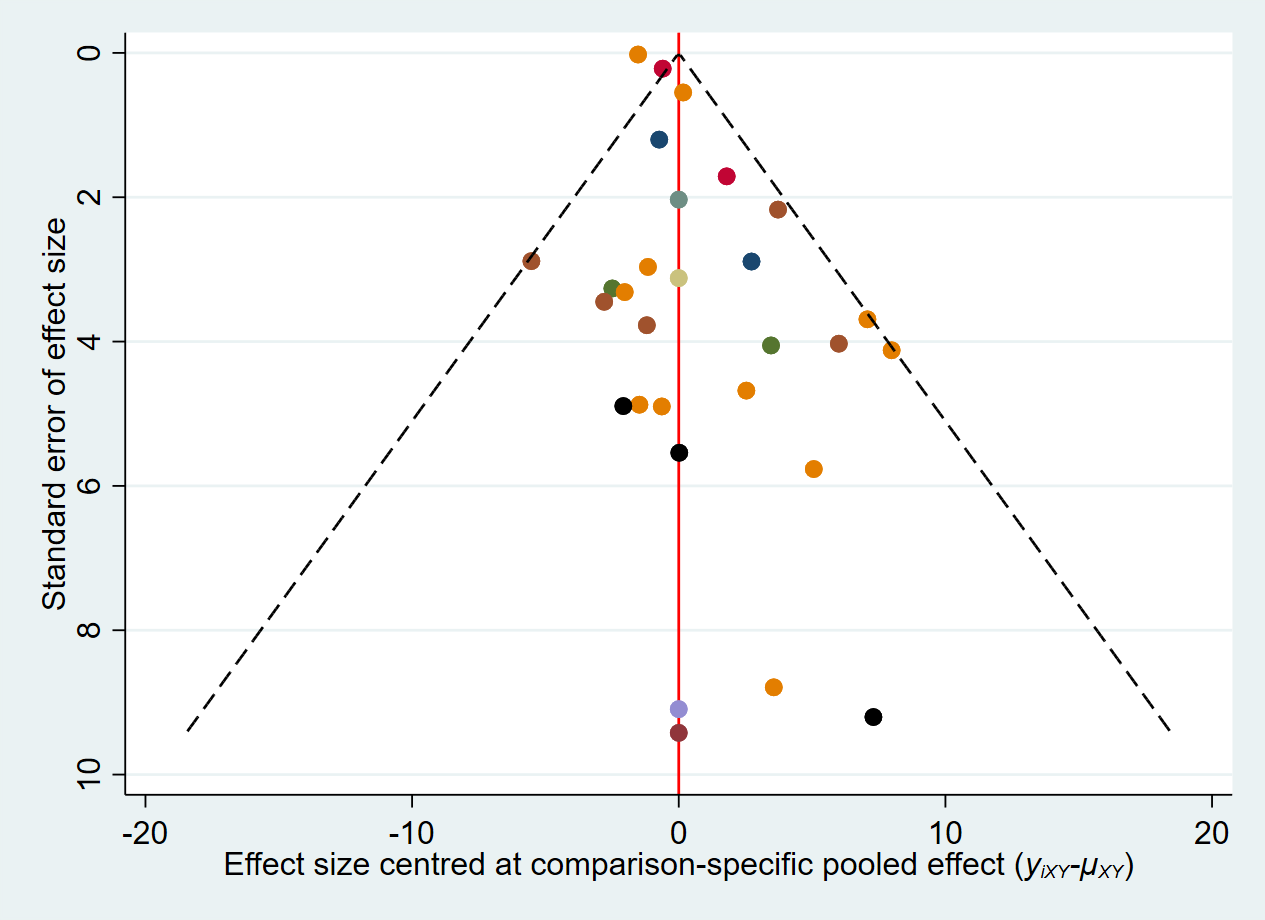


### (J) Insulin


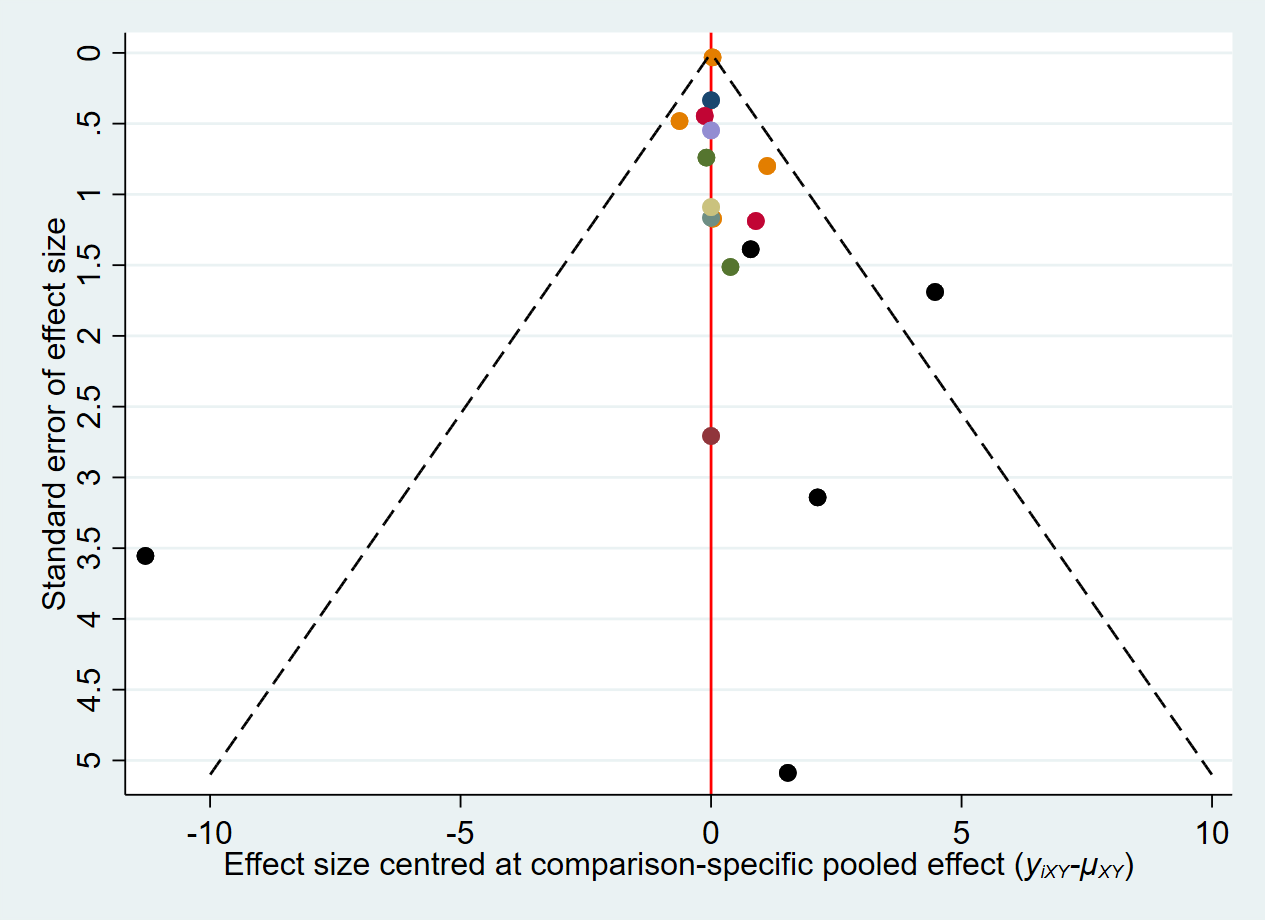


### (K) HOMA-IR


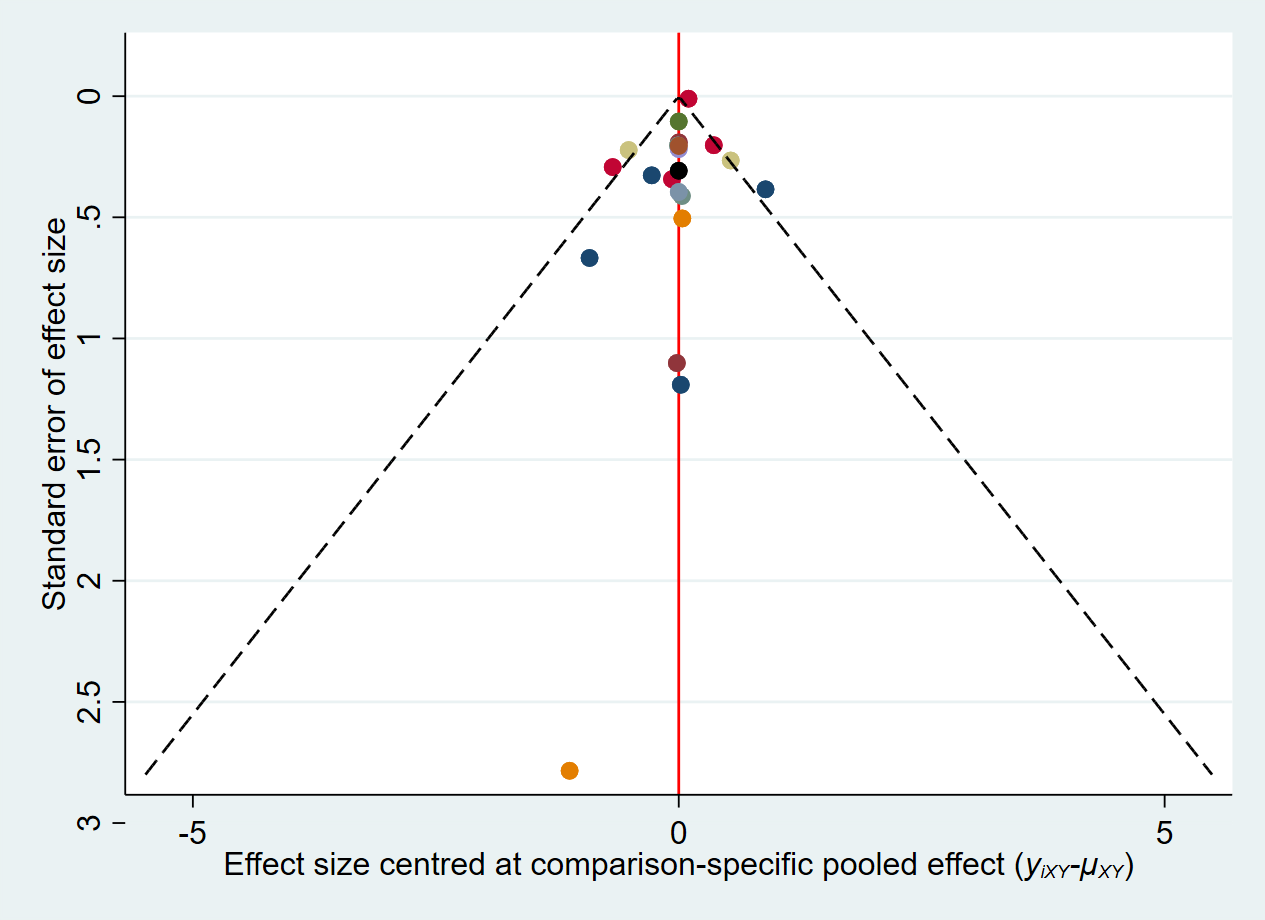


## **Table 1~Table 11: League tables of other outcome measures**

Table 1 League table of Weight of different dietary polyphenols

| Placebo | Ellagic acid | Anthocyanin | Genistin/Genistein | Chlorogenic acid | Curcumin | Naringenin | Catechin | Resveratrol | Dihydromyricetin | Silymarin |
| --- | --- | --- | --- | --- | --- | --- | --- | --- | --- | --- |
| Placebo | -4.49 (-5.29, -3.7) | -0.05 (-1.1, 0.99) | 0.45 (-2.6, 3.49) | -1.29 (-1.95, -0.62) | -0.54 (-0.91, -0.16) | -1.7 (-2.73, -0.68) | -1.72 (-2.52, -0.91) | -0.76 (-1.31, -0.21) | 0.24 (-1.06, 1.55) | -0.6 (-1.45, 0.24) |
| **4.49 (3.7, 5.29)** | Ellagic acid | 4.44 (3.13, 5.75) | 4.94 (1.79, 8.08) | 3.21 (2.17, 4.24) | 3.95 (3.08, 4.83) | 2.79 (1.49, 4.09) | 2.77 (1.64, 3.91) | 3.73 (2.76, 4.7) | 4.74 (3.21, 6.27) | 3.89 (2.73, 5.05) |
| 0.05 (-0.99, 1.1) | **-4.44 (-5.75, -3.13)** | Anthocyanin | 0.5 (-2.72, 3.7) | -1.23 (-2.47, 0.01) | -0.48 (-1.59, 0.63) | -1.65 (-3.11, -0.18) | -1.66 (-2.99, -0.35) | -0.71 (-1.88, 0.47) | 0.3 (-1.37, 1.97) | -0.55 (-1.88, 0.79) |
| -0.45 (-3.49, 2.6) | **-4.94 (-8.08, -1.79)** | -0.5 (-3.7, 2.72) | Genistin/Genistein | -1.73 (-4.84, 1.38) | -0.98 (-4.04, 2.09) | -2.14 (-5.34, 1.06) | -2.16 (-5.32, 0.98) | -1.2 (-4.3, 1.88) | -0.2 (-3.52, 3.12) | -1.05 (-4.21, 2.12) |
| **1.29 (0.62, 1.95)** | **-3.21 (-4.24, -2.17)** | 1.23 (-0.01, 2.47) | 1.73 (-1.38, 4.84) | Chlorogenic acid | 0.75 (-0.01, 1.51) | -0.41 (-1.63, 0.8) | -0.43 (-1.47, 0.61) | 0.53 (-0.33, 1.39) | 1.53 (0.07, 2.99) | 0.69 (-0.39, 1.76) |
| **0.54 (0.16, 0.91)** | **-3.95 (-4.83, -3.08)** | 0.48 (-0.63, 1.59) | 0.98 (-2.09, 4.04) | -0.75 (-1.51, 0.01) | Curcumin | -1.16 (-2.26, -0.07) | -1.18 (-2.07, -0.3) | -0.22 (-0.89, 0.44) | 0.78 (-0.57, 2.14) | -0.07 (-0.99, 0.86) |
| **1.7 (0.68, 2.73)** | **-2.79 (-4.09, -1.49)** | **1.65 (0.18, 3.11)** | 2.14 (-1.06, 5.34) | 0.41 (-0.8, 1.63) | **1.16 (0.07, 2.26)** | Naringenin | -0.02 (-1.32, 1.29) | 0.94 (-0.22, 2.11) | 1.95 (0.28, 3.61) | 1.1 (-0.23, 2.43) |
| **1.72 (0.91, 2.52)** | **-2.77 (-3.91, -1.64)** | **1.66 (0.35, 2.99)** | 2.16 (-0.98, 5.32) | 0.43 (-0.61, 1.47) | **1.18 (0.3, 2.07)** | 0.02 (-1.29, 1.32) | Catechin | 0.96 (-0.02, 1.93) | 1.97 (0.43, 3.5) | 1.12 (-0.05, 2.29) |
| **0.76 (0.21, 1.31)** | **-3.73 (-4.7, -2.76)** | 0.71 (-0.47, 1.88) | 1.2 (-1.88, 4.3) | -0.53 (-1.39, 0.33) | 0.22 (-0.44, 0.89) | -0.94 (-2.11, 0.22) | -0.96 (-1.93, 0.02) | Resveratrol | 1 (-0.41, 2.42) | 0.16 (-0.85, 1.17) |
| -0.24 (-1.55, 1.06) | **-4.74 (-6.27, -3.21)** | -0.3 (-1.97, 1.37) | 0.2 (-3.12, 3.52) | **-1.53 (-2.99, -0.07)** | -0.78 (-2.14, 0.57) | **-1.95 (-3.61, -0.28)** | **-1.97 (-3.5, -0.43)** | -1 (-2.42, 0.41) | Dihydromyricetin | -0.85 (-2.4, 0.7) |
| 0.6 (-0.24, 1.45) | **-3.89 (-5.05, -2.73)** | 0.55 (-0.79, 1.88) | 1.05 (-2.12, 4.21) | -0.69 (-1.76, 0.39) | 0.07 (-0.86, 0.99) | -1.1 (-2.43, 0.23) | -1.12 (-2.29, 0.05) | -0.16 (-1.17, 0.85) | 0.85 (-0.7, 2.4) | Silymarin |

Table 2 League table of WC of different dietary polyphenols

| Placebo | Ellagic acid | Anthocyanin | Genistin/Genistein | Chlorogenic acid | Curcumin | Naringenin | Resveratrol | Dihydromyricetin | Silymarin |
| --- | --- | --- | --- | --- | --- | --- | --- | --- | --- |
| Placebo | -5.56 (-9.43, -1.46) | 0.33 (-3, 3.68) | -0.95 (-5.31, 3.39) | -1.79 (-5.75, 2.3) | -2.07 (-3.94, -0.2) | -1.72 (-6.58, 3.14) | -2.56 (-5.4, 0.43) | 0.41 (-5.14, 5.93) | 0.78 (-5.69, 7.29) |
| **5.56 (1.46, 9.43)** | Ellagic acid | 5.9 (0.57, 10.99) | 4.62 (-1.42, 10.38) | 3.77 (-1.96, 9.37) | 3.5 (-1.03, 7.76) | 3.84 (-2.57, 10) | 3 (-1.97, 7.89) | 5.97 (-0.96, 12.7) | 6.34 (-1.38, 13.9) |
| -0.33 (-3.68, 3) | **-5.9 (-10.99, -0.57)** | Anthocyanin | -1.28 (-6.77, 4.18) | -2.11 (-7.32, 3.14) | -2.4 (-6.24, 1.41) | -2.05 (-7.99, 3.81) | -2.88 (-7.27, 1.6) | 0.08 (-6.39, 6.51) | 0.44 (-6.85, 7.74) |
| 0.95 (-3.39, 5.31) | -4.62 (-10.38, 1.42) | 1.28 (-4.18, 6.77) | Genistin/Genistein | -0.83 (-6.71, 5.15) | -1.11 (-5.84, 3.62) | -0.77 (-7.29, 5.77) | -1.6 (-6.77, 3.72) | 1.36 (-5.7, 8.42) | 1.74 (-6.08, 9.6) |
| 1.79 (-2.3, 5.75) | -3.77 (-9.37, 1.96) | 2.11 (-3.14, 7.32) | 0.83 (-5.15, 6.71) | Chlorogenic acid | -0.28 (-4.77, 4.1) | 0.07 (-6.3, 6.3) | -0.76 (-5.73, 4.21) | 2.2 (-4.72, 8.98) | 2.56 (-5.13, 10.17) |
| **2.07 (0.2, 3.94)** | -3.5 (-7.76, 1.03) | 2.4 (-1.41, 6.24) | 1.11 (-3.62, 5.84) | 0.28 (-4.1, 4.77) | Curcumin | 0.35 (-4.87, 5.56) | -0.49 (-3.88, 3.07) | 2.49 (-3.36, 8.31) | 2.85 (-3.89, 9.62) |
| 1.72 (-3.14, 6.58) | -3.84 (-10, 2.57) | 2.05 (-3.81, 7.99) | 0.77 (-5.77, 7.29) | -0.07 (-6.3, 6.3) | -0.35 (-5.56, 4.87) | Naringenin | -0.84 (-6.41, 4.92) | 2.14 (-5.27, 9.47) | 2.51 (-5.57, 10.62) |
| 2.56 (-0.43, 5.4) | -3 (-7.89, 1.97) | 2.88 (-1.6, 7.27) | 1.6 (-3.72, 6.77) | 0.76 (-4.21, 5.73) | 0.49 (-3.07, 3.88) | 0.84 (-4.92, 6.41) | Resveratrol | 2.97 (-3.37, 9.14) | 3.33 (-3.81, 10.41) |
| -0.41 (-5.93, 5.14) | -5.97 (-12.7, 0.96) | -0.08 (-6.51, 6.39) | -1.36 (-8.42, 5.7) | -2.2 (-8.98, 4.72) | -2.49 (-8.31, 3.36) | -2.14 (-9.47, 5.27) | -2.97 (-9.14, 3.37) | Dihydromyricetin | 0.37 (-8.14, 8.95) |
| -0.78 (-7.29, 5.69) | -6.34 (-13.9, 1.38) | -0.44 (-7.74, 6.85) | -1.74 (-9.6, 6.08) | -2.56 (-10.17, 5.13) | -2.85 (-9.62, 3.89) | -2.51 (-10.62, 5.57) | -3.33 (-10.41, 3.81) | -0.37 (-8.95, 8.14) | Silymarin |

Table 3 League table of HC of different dietary polyphenols

| Placebo | Anthocyanin | Genistin/Genistein | Chlorogenic acid | Curcumin | Resveratrol | Dihydromyricetin |
| --- | --- | --- | --- | --- | --- | --- |
| Placebo | -0.73 (-1.76, 0.29) | 0.07 (-4.39, 4.49) | -0.46 (-4.36, 3.45) | 0.15 (-0.29, 0.59) | -0.23 (-1.16, 0.7) | -0.06 (-3.56, 3.42) |
| 0.73 (-0.29, 1.76) | Anthocyanin | 0.8 (-3.78, 5.34) | 0.27 (-3.77, 4.3) | 0.88 (-0.23, 2) | 0.5 (-0.88, 1.88) | 0.66 (-2.98, 4.3) |
| -0.07 (-4.49, 4.39) | -0.8 (-5.34, 3.78) | Genistin/Genistein | -0.52 (-6.42, 5.41) | 0.08 (-4.36, 4.56) | -0.3 (-4.82, 4.26) | -0.13 (-5.78, 5.51) |
| 0.46 (-3.45, 4.36) | -0.27 (-4.3, 3.77) | 0.52 (-5.41, 6.42) | Chlorogenic acid | 0.62 (-3.32, 4.55) | 0.24 (-3.78, 4.24) | 0.41 (-4.83, 5.62) |
| -0.15 (-0.59, 0.29) | -0.88 (-2, 0.23) | -0.08 (-4.56, 4.36) | -0.62 (-4.55, 3.32) | Curcumin | -0.38 (-1.41, 0.65) | -0.21 (-3.74, 3.29) |
| 0.23 (-0.7, 1.16) | -0.5 (-1.88, 0.88) | 0.3 (-4.26, 4.82) | -0.24 (-4.24, 3.78) | 0.38 (-0.65, 1.41) | Resveratrol | 0.17 (-3.45, 3.76) |
| 0.06 (-3.42, 3.56) | -0.66 (-4.3, 2.98) | 0.13 (-5.51, 5.78) | -0.41 (-5.62, 4.83) | 0.21 (-3.29, 3.74) | -0.17 (-3.76, 3.45) | Dihydromyricetin |

Table 4 League table of WHR of different dietary polyphenols

| Placebo | Anthocyanin | Genistin/Genistein | Chlorogenic acid | Curcumin | Resveratro | Dihydromyricetin | Quercetin |
| --- | --- | --- | --- | --- | --- | --- | --- |
| Placebo | 0.01 (0, 0.02) | -0.01 (-0.03, 0.01) | -0.01 (-0.03, 0.01) | 0 (0, 0.01) | 0 (-0.02, 0.01) | 0 (-0.03, 0.03) | -0.01 (-0.03, 0.01) |
| **-0.01 (-0.02, 0)** | Anthocyanin | -0.02 (-0.05, 0) | -0.02 (-0.04, 0) | -0.01 (-0.02, 0.01) | -0.01 (-0.04, 0.01) | -0.01 (-0.04, 0.02) | -0.02 (-0.04, 0) |
| 0.01 (-0.01, 0.03) | **0.02 (0, 0.05)** | Genistin/Genistein | 0 (-0.02, 0.03) | 0.02 (0, 0.04) | 0.01 (-0.02, 0.04) | 0.01 (-0.02, 0.05) | 0 (-0.02, 0.03) |
| 0.01 (-0.01, 0.03) | **0.02 (0, 0.04)** | 0 (-0.03, 0.02) | Chlorogenic acid | 0.01 (-0.01, 0.03) | 0.01 (-0.02, 0.03) | 0.01 (-0.03, 0.05) | 0 (-0.02, 0.03) |
| **0 (-0.01, 0)** | 0.01 (-0.01, 0.02) | **-0.02 (-0.04, 0)** | -0.01 (-0.03, 0.01) | Curcumin | -0.01 (-0.03, 0.01) | 0 (-0.03, 0.03) | -0.01 (-0.03, 0) |
| 0 (-0.01, 0.02) | 0.01 (-0.01, 0.04) | -0.01 (-0.04, 0.02) | -0.01 (-0.03, 0.02) | 0.01 (-0.01, 0.03) | Resveratrol | 0 (-0.03, 0.04) | -0.01 (-0.03, 0.02) |
| 0 (-0.03, 0.03) | 0.01 (-0.02, 0.04) | -0.01 (-0.05, 0.02) | -0.01 (-0.05, 0.03) | 0 (-0.03, 0.03) | 0 (-0.04, 0.03) | Dihydromyricetin | -0.01 (-0.04, 0.03) |
| 0.01 (-0.01, 0.03) | **0.02 (0, 0.04)** | 0 (-0.03, 0.02) | 0 (-0.03, 0.02) | **0.01 (0, 0.03)** | 0.01 (-0.02, 0.03) | 0.01 (-0.03, 0.04) | Quercetin |

Table 5 League table of SBP of different dietary polyphenols

| Placebo | Ellagic acid | Anthocyanin | Genistin/Genistein | Chlorogenic acid | Curcumin | Naringenin | Resveratrol | Dihydromyricetin |
| --- | --- | --- | --- | --- | --- | --- | --- | --- |
| Placebo | -4.99 (-8.73, 0.04) | -1.54 (-5.68, 2.09) | 0.56 (-7.32, 8.42) | -9.74 (-16.48, -3) | -0.97 (-3.65, 1.11) | -5.93 (-15.89, 4.15) | -4.21 (-7.83, 0.09) | 3.14 (-6.58, 12.92) |
| 4.99 (-0.04, 8.73) | Ellagic acid | 3.44 (-3.26, 8.44) | 5.44 (-3.86, 14.06) | -4.81 (-13.37, 2.71) | 4 (-1.9, 8.13) | -1.06 (-12.15, 9.68) | 0.68 (-5.04, 6.45) | 8 (-2.87, 18.42) |
| 1.54 (-2.09, 5.68) | -3.44 (-8.44, 3.26) | Anthocyanin | 2.14 (-6.42, 11.02) | -8.18 (-15.71, -0.23) | 0.56 (-4.01, 5.07) | -4.34 (-14.95, 6.54) | -2.69 (-7.58, 3.53) | 4.72 (-5.6, 15.38) |
| -0.56 (-8.42, 7.32) | -5.44 (-14.06, 3.86) | -2.14 (-11.02, 6.42) | Genistin/Genistein | -10.32 (-20.68, 0.07) | -1.58 (-9.89, 6.46) | -6.5 (-19.09, 6.33) | -4.76 (-13.29, 4.38) | 2.56 (-9.97, 15.26) |
| **9.74 (3, 16.48)** | 4.81 (-2.71, 13.37) | **8.18 (0.23, 15.71)** | 10.32 (-0.07, 20.68) | Chlorogenic acid | 8.73 (1.39, 15.65) | 3.82 (-8.21, 15.96) | 5.51 (-1.9, 13.75) | 12.88 (1.05, 24.79) |
| 0.97 (-1.11, 3.65) | -4 (-8.13, 1.9) | -0.56 (-5.07, 4.01) | 1.58 (-6.46, 9.89) | -8.73 (-15.65, -1.39) | Curcumin | -4.88 (-15.02, 5.54) | -3.22 (-7.28, 2.03) | 4.18 (-5.7, 14.33) |
| 5.93 (-4.15, 15.89) | 1.06 (-9.68, 12.15) | 4.34 (-6.54, 14.95) | 6.5 (-6.33, 19.09) | -3.82 (-15.96, 8.21) | 4.88 (-5.54, 15.02) | Naringenin | 1.75 (-8.9, 12.6) | 9.08 (-5.05, 23.11) |
| 4.21 (-0.09, 7.83) | -0.68 (-6.45, 5.04) | 2.69 (-3.53, 7.58) | 4.76 (-4.38, 13.29) | -5.51 (-13.75, 1.9) | 3.22 (-2.03, 7.28) | -1.75 (-12.6, 8.9) | Resveratrol | 7.32 (-3.42, 17.69) |
| -3.14 (-12.92, 6.58) | -8 (-18.42, 2.87) | -4.72 (-15.38, 5.6) | -2.56 (-15.26, 9.97) | -12.88 (-24.79, -1.05) | -4.18 (-14.33, 5.7) | -9.08 (-23.11, 5.05) | -7.32 (-17.69, 3.42) | Dihydromyricetin |

Table 6 League table of DBP of different dietary polyphenols

| Placebo | Ellagic acid | Anthocyanin | Genistin/Genistein | Chlorogenic acid | Curcumin | Naringenin | Resveratrol | Dihydromyricetin |
| --- | --- | --- | --- | --- | --- | --- | --- | --- |
| Placebo | -4.09 (-5.12, -3.04) | -0.3 (-1.02, 0.42) | 1.01 (-3.35, 5.36) | -3.52 (-7.41, 0.35) | 0 (-0.22, 0.22) | -0.85 (-7.85, 6.14) | -1.44 (-3.76, 0.89) | 2.75 (-1.73, 7.24) |
| **4.09 (3.04, 5.12)** | Ellagic acid | 3.78 (2.52, 5.05) | 5.1 (0.62, 9.56) | 0.57 (-3.46, 4.58) | 4.09 (3.02, 5.15) | 3.23 (-3.86, 10.3) | 2.65 (0.09, 5.2) | 6.83 (2.23, 11.44) |
| 0.3 (-0.42, 1.02) | **-3.78 (-5.05, -2.52)** | Anthocyanin | 1.31 (-3.1, 5.73) | -3.22 (-7.17, 0.73) | 0.3 (-0.45, 1.05) | -0.55 (-7.59, 6.47) | -1.14 (-3.59, 1.31) | 3.06 (-1.5, 7.59) |
| -1.01 (-5.36, 3.35) | **-5.1 (-9.56, -0.62)** | -1.31 (-5.73, 3.1) | Genistin/Genistein | -4.53 (-10.39, 1.31) | -1.01 (-5.36, 3.35) | -1.87 (-10.15, 6.35) | -2.45 (-7.37, 2.48) | 1.74 (-4.53, 8) |
| 3.52 (-0.35, 7.41) | -0.57 (-4.58, 3.46) | 3.22 (-0.73, 7.17) | 4.53 (-1.31, 10.39) | Chlorogenic acid | 3.52 (-0.35, 7.42) | 2.66 (-5.34, 10.7) | 2.08 (-2.44, 6.6) | 6.27 (0.32, 12.22) |
| 0 (-0.22, 0.22) | **-4.09 (-5.15, -3.02)** | -0.3 (-1.05, 0.45) | 1.01 (-3.35, 5.36) | -3.52 (-7.42, 0.35) | Curcumin | -0.85 (-7.85, 6.14) | -1.44 (-3.77, 0.9) | 2.75 (-1.73, 7.25) |
| 0.85 (-6.14, 7.85) | -3.23 (-10.3, 3.86) | 0.55 (-6.47, 7.59) | 1.87 (-6.35, 10.15) | -2.66 (-10.7, 5.34) | 0.85 (-6.14, 7.85) | Naringenin | -0.58 (-7.95, 6.81) | 3.6 (-4.73, 11.92) |
| 1.44 (-0.89, 3.76) | **-2.65 (-5.2, -0.09)** | 1.14 (-1.31, 3.59) | 2.45 (-2.48, 7.37) | -2.08 (-6.6, 2.44) | 1.44 (-0.9, 3.77) | 0.58 (-6.81, 7.95) | Resveratrol | 4.19 (-0.89, 9.23) |
| -2.75 (-7.24, 1.73) | **-6.83 (-11.44, -2.23)** | -3.06 (-7.59, 1.5) | -1.74 (-8, 4.53) | **-6.27 (-12.22, -0.32)** | -2.75 (-7.25, 1.73) | -3.6 (-11.92, 4.73) | -4.19 (-9.23, 0.89) | Dihydromyricetin |

Table 7 League table of ALP of different dietary polyphenols

| Placebo | Ellagic acid | Curcumin | Catechin | Resveratrol | Dihydromyricetin | Silymarin | Gallic acid and Chlorogenic acid |
| --- | --- | --- | --- | --- | --- | --- | --- |
| Placebo | -7.21 (-33.95, 19.39) | -5.51 (-10.43, -0.56) | -28.14 (-41.19, -15.11) | 9.75 (-5.87, 25.29) | -6.04 (-10.63, -1.44) | -6.87 (-14.94, 1.2) | -8.35 (-16.12, -0.58) |
| 7.21 (-19.39, 33.95) | Ellagic acid | 1.74 (-25.36, 28.89) | -20.9 (-50.41, 8.88) | 16.96 (-13.94, 47.87) | 1.17 (-25.77, 28.26) | 0.37 (-27.49, 28.27) | -1.16 (-28.89, 26.72) |
| **5.51 (0.56, 10.43)** | -1.74 (-28.89, 25.36) | Curcumin | -22.61 (-36.64, -8.76) | 15.26 (-1.03, 31.55) | -0.54 (-7.26, 6.21) | -1.35 (-10.81, 8.08) | -2.85 (-12.04, 6.32) |
| **28.14 (15.11, 41.19)** | 20.9 (-8.88, 50.41) | **22.61 (8.76, 36.64)** | Catechin | 37.89 (17.5, 58.27) | 22.1 (8.22, 35.93) | 21.28 (5.92, 36.61) | 19.78 (4.64, 34.93) |
| -9.75 (-25.29, 5.87) | -16.96 (-47.87, 13.94) | -15.26 (-31.55, 1.03) | **-37.89 (-58.27, -17.5)** | Resveratrol | -15.8 (-32.04, 0.46) | -16.61 (-34.09, 0.96) | -18.13 (-35.55, -0.65) |
| **6.04 (1.44, 10.63)** | -1.17 (-28.26, 25.77) | 0.54 (-6.21, 7.26) | **-22.1 (-35.93, -8.22)** | 15.8 (-0.46, 32.04) | Dihydromyricetin | -0.81 (-10.13, 8.46) | -2.31 (-11.35, 6.73) |
| 6.87 (-1.2, 14.94) | -0.37 (-28.27, 27.49) | 1.35 (-8.08, 10.81) | **-21.28 (-36.61, -5.92)** | 16.61 (-0.96, 34.09) | 0.81 (-8.46, 10.13) | Silymarin | -1.5 (-12.74, 9.71) |
| **8.35 (0.58, 16.12)** | 1.16 (-26.72, 28.89) | 2.85 (-6.32, 12.04) | **-19.78 (-34.93, -4.64)** | **18.13 (0.65, 35.55)** | 2.31 (-6.73, 11.35) | 1.5 (-9.71, 12.74) | Gallic acid and Chlorogenic acid |

Table 8 League table of GGT of different dietary polyphenols

| Placebo | Genistin/Genistein | Curcumin | Resveratrol | Dihydromyricetin | Silymarin | Gallic acid and Chlorogenic acid | Hesperidin | Quercetin |
| --- | --- | --- | --- | --- | --- | --- | --- | --- |
| Placebo | 4.88 (-3.8, 13.44) | -3.3 (-7.25, 0.61) | -1.83 (-5.77, 2.1) | -7.21 (-12.15, -2.31) | -8.02 (-12.31, -3.76) | -10.85 (-15.25, -6.4) | -10.95 (-17.41, -4.55) | -1.64 (-2.06, -1.22) |
| -4.88 (-13.44, 3.8) | Genistin/Genistein | -8.19 (-17.64, 1.36) | -6.72 (-16.15, 2.87) | -12.1 (-21.96, -2.08) | -12.9 (-22.48, -3.27) | -15.72 (-25.4, -5.99) | -15.84 (-26.54, -5.11) | -6.52 (-15.09, 2.17) |
| 3.3 (-0.61, 7.25) | 8.19 (-1.36, 17.64) | Curcumin | 1.47 (-4.09, 7.03) | -3.91 (-10.21, 2.36) | -4.73 (-10.52, 1.08) | -7.55 (-13.49, -1.6) | -7.66 (-15.21, -0.18) | 1.65 (-2.28, 5.63) |
| 1.83 (-2.1, 5.77) | 6.72 (-2.87, 16.15) | -1.47 (-7.03, 4.09) | Resveratrol | -5.38 (-11.67, 0.91) | -6.18 (-12.02, -0.43) | -9.01 (-14.94, -3.09) | -9.11 (-16.7, -1.63) | 0.19 (-3.76, 4.15) |
| **7.21 (2.31, 12.15)** | **12.1 (2.08, 21.96)** | 3.91 (-2.36, 10.21) | 5.38 (-0.91, 11.67) | Dihydromyricetin | -0.8 (-7.32, 5.7) | -3.63 (-10.24, 2.99) | -3.74 (-11.8, 4.35) | 5.57 (0.64, 10.52) |
| **8.02 (3.76, 12.31)** | **12.9 (3.27, 22.48)** | 4.73 (-1.08, 10.52) | **6.18 (0.43, 12.02)** | 0.8 (-5.7, 7.32) | Silymarin | -2.82 (-8.98, 3.34) | -2.93 (-10.63, 4.77) | 6.38 (2.1, 10.69) |
| **10.85 (6.4, 15.25)** | **15.72 (5.99, 25.4)** | **7.55 (1.6, 13.49)** | **9.01 (3.09, 14.94)** | 3.63 (-2.99, 10.24) | 2.82 (-3.34, 8.98) | Gallic acid and Chlorogenic acid | -0.1 (-7.97, 7.66) | 9.21 (4.75, 13.62) |
| **10.95 (4.55, 17.41)** | **15.84 (5.11, 26.54)** | **7.66 (0.18, 15.21)** | **9.11 (1.63, 16.7)** | 3.74 (-4.35, 11.8) | 2.93 (-4.77, 10.63) | 0.1 (-7.66, 7.97) | Hesperidin | 9.31 (2.9, 15.78) |
| **1.64 (1.22, 2.06)** | 6.52 (-2.17, 15.09) | -1.65 (-5.63, 2.28) | -0.19 (-4.15, 3.76) | **-5.57 (-10.52, -0.64)** | **-6.38 (-10.69, -2.1)** | **-9.21 (-13.62, -4.75)** | **-9.31 (-15.78, -2.9)** | Quercetin |

Table 9 League table of FBG of different dietary polyphenols

| Placebo | Ellagic acid | Anthocyanin | Genistin/Genistein | Chlorogenic acid | Curcumin | Catechin | Resveratrol | Dihydromyricetin | Silymarin | Hesperidin |
| --- | --- | --- | --- | --- | --- | --- | --- | --- | --- | --- |
| Placebo | -5.64 (-6.06, -5.21) | 0.25 (-1.91, 2.43) | -6.95 (-24.78, 10.65) | -6.9 (-11.9, -1.9) | -0.01 (-0.06, 0.03) | -7.18 (-25.58, 11.24) | -3.88 (-6.53, -1.21) | -8.27 (-12.28, -4.28) | -5.1 (-11.74, 1.59) | -4.32 (-10.42, 1.8) |
| **5.64 (5.21, 6.06)** | Ellagic acid | 5.89 (3.68, 8.1) | -1.31 (-19.13, 16.29) | -1.26 (-6.28, 3.75) | 5.62 (5.19, 6.05) | -1.54 (-19.94, 16.89) | 1.76 (-0.93, 4.46) | -2.64 (-6.67, 1.38) | 0.54 (-6.12, 7.23) | 1.31 (-4.81, 7.46) |
| -0.25 (-2.43, 1.91) | **-5.89 (-8.1, -3.68)** | Anthocyanin | -7.21 (-25.05, 10.52) | -7.15 (-12.61, -1.68) | -0.26 (-2.44, 1.9) | -7.44 (-25.95, 11.15) | -4.14 (-7.56, -0.69) | -8.52 (-13.11, -3.98) | -5.35 (-12.36, 1.69) | -4.57 (-11.03, 1.94) |
| 6.95 (-10.65, 24.78) | 1.31 (-16.29, 19.13) | 7.21 (-10.52, 25.05) | Genistin/Genistein | 0.05 (-18.15, 18.58) | 6.93 (-10.66, 24.76) | -0.26 (-25.52, 25.4) | 3.05 (-14.66, 21.11) | -1.35 (-19.27, 16.97) | 1.89 (-17, 20.85) | 2.64 (-16.03, 21.47) |
| **6.9 (1.9, 11.9)** | 1.26 (-3.75, 6.28) | **7.15 (1.68, 12.61)** | -0.05 (-18.58, 18.15) | Chlorogenic acid | 6.89 (1.89, 11.88) | -0.29 (-19.39, 18.8) | 3.01 (-2.62, 8.68) | -1.39 (-7.8, 5) | 1.81 (-6.53, 10.12) | 2.59 (-5.28, 10.46) |
| 0.01 (-0.03, 0.06) | **-5.62 (-6.05, -5.19)** | 0.26 (-1.9, 2.44) | -6.93 (-24.76, 10.66) | **-6.89 (-11.88, -1.89)** | Curcumin | -7.16 (-25.56, 11.27) | -3.87 (-6.51, -1.2) | -8.26 (-12.27, -4.27) | -5.08 (-11.73, 1.61) | -4.31 (-10.4, 1.82) |
| 7.18 (-11.24, 25.58) | 1.54 (-16.89, 19.94) | 7.44 (-11.15, 25.95) | 0.26 (-25.4, 25.52) | 0.29 (-18.8, 19.39) | 7.16 (-11.27, 25.56) | Catechin | 3.3 (-15.32, 21.95) | -1.1 (-19.97, 17.75) | 2.09 (-17.51, 21.64) | 2.88 (-16.6, 22.14) |
| **3.88 (1.21, 6.53)** | -1.76 (-4.46, 0.93) | **4.14 (0.69, 7.56)** | -3.05 (-21.11, 14.66) | -3.01 (-8.68, 2.62) | **3.87 (1.2, 6.51)** | -3.3 (-21.95, 15.32) | Resveratrol | -4.39 (-9.2, 0.38) | -1.2 (-8.39, 5.96) | -0.43 (-7.08, 6.26) |
| **8.27 (4.28, 12.28)** | 2.64 (-1.38, 6.67) | **8.52 (3.98, 13.11)** | 1.35 (-16.97, 19.27) | 1.39 (-5, 7.8) | **8.26 (4.27, 12.27)** | 1.1 (-17.75, 19.97) | 4.39 (-0.38, 9.2) | Dihydromyricetin | 3.18 (-4.56, 10.94) | 3.95 (-3.36, 11.29) |
| 5.1 (-1.59, 11.74) | -0.54 (-7.23, 6.12) | 5.35 (-1.69, 12.36) | -1.89 (-20.85, 17) | -1.81 (-10.12, 6.53) | 5.08 (-1.61, 11.73) | -2.09 (-21.64, 17.51) | 1.2 (-5.96, 8.39) | -3.18 (-10.94, 4.56) | Silymarin | 0.79 (-8.24, 9.78) |
| 4.32 (-1.8, 10.42) | -1.31 (-7.46, 4.81) | 4.57 (-1.94, 11.03) | -2.64 (-21.47, 16.03) | -2.59 (-10.46, 5.28) | 4.31 (-1.82, 10.4) | -2.88 (-22.14, 16.6) | 0.43 (-6.26, 7.08) | -3.95 (-11.29, 3.36) | -0.79 (-9.78, 8.24) | Hesperidin |

Table 10 League table of insulin of different dietary polyphenols

| Placebo | Ellagic acid | Anthocyanin | Genistin/Genistein | Chlorogenic acid | Curcumin | Catechin | Resveratrol | Dihydromyricetin | Hesperidin |
| --- | --- | --- | --- | --- | --- | --- | --- | --- | --- |
| Placebo | -1.17 (-1.99, -0.36) | -0.26 (-0.92, 0.4) | -1.25 (-2.32, -0.18) | -0.81 (-2.12, 0.48) | -1.2 (-1.26, -1.14) | -1.19 (-6.48, 4.11) | -1.03 (-2.91, 0.85) | -1.84 (-4.12, 0.45) | 0.95 (-1.18, 3.09) |
| **1.17 (0.36, 1.99)** | Ellagic acid | 0.91 (-0.13, 1.96) | -0.07 (-1.42, 1.28) | 0.36 (-1.18, 1.89) | -0.03 (-0.84, 0.79) | -0.02 (-5.36, 5.35) | 0.15 (-1.91, 2.2) | -0.66 (-3.09, 1.77) | 2.13 (-0.17, 4.41) |
| 0.26 (-0.4, 0.92) | -0.91 (-1.96, 0.13) | Anthocyanin | -0.98 (-2.25, 0.28) | -0.55 (-2.01, 0.9) | -0.94 (-1.6, -0.28) | -0.93 (-6.27, 4.41) | -0.77 (-2.76, 1.22) | -1.58 (-3.96, 0.81) | 1.21 (-1.03, 3.46) |
| **1.25 (0.18, 2.32)** | 0.07 (-1.28, 1.42) | 0.98 (-0.28, 2.25) | Genistin/Genistein | 0.43 (-1.26, 2.12) | 0.05 (-1.03, 1.13) | 0.06 (-5.32, 5.46) | 0.22 (-1.95, 2.38) | -0.59 (-3.11, 1.94) | 2.2 (-0.2, 4.59) |
| 0.81 (-0.48, 2.12) | -0.36 (-1.89, 1.18) | 0.55 (-0.9, 2.01) | -0.43 (-2.12, 1.26) | Chlorogenic acid | -0.39 (-1.68, 0.92) | -0.38 (-5.83, 5.08) | -0.22 (-2.5, 2.08) | -1.02 (-3.66, 1.61) | 1.77 (-0.74, 4.27) |
| **1.2 (1.14, 1.26)** | 0.03 (-0.79, 0.84) | **0.94 (0.28, 1.6)** | -0.05 (-1.13, 1.03) | 0.39 (-0.92, 1.68) | Curcumin | 0.01 (-5.28, 5.3) | 0.17 (-1.71, 2.05) | -0.64 (-2.93, 1.65) | 2.15 (0.02, 4.29) |
| 1.19 (-4.11, 6.48) | 0.02 (-5.35, 5.36) | 0.93 (-4.41, 6.27) | -0.06 (-5.46, 5.32) | 0.38 (-5.08, 5.83) | -0.01 (-5.3, 5.28) | Catechin | 0.16 (-5.47, 5.78) | -0.64 (-6.43, 5.12) | 2.14 (-3.58, 7.86) |
| 1.03 (-0.85, 2.91) | -0.15 (-2.2, 1.91) | 0.77 (-1.22, 2.76) | -0.22 (-2.38, 1.95) | 0.22 (-2.08, 2.5) | -0.17 (-2.05, 1.71) | -0.16 (-5.78, 5.47) | Resveratrol | -0.81 (-3.77, 2.16) | 1.98 (-0.86, 4.83) |
| 1.84 (-0.45, 4.12) | 0.66 (-1.77, 3.09) | 1.58 (-0.81, 3.96) | 0.59 (-1.94, 3.11) | 1.02 (-1.61, 3.66) | 0.64 (-1.65, 2.93) | 0.64 (-5.12, 6.43) | 0.81 (-2.16, 3.77) | Dihydromyricetin | 2.79 (-0.35, 5.93) |
| -0.95 (-3.09, 1.18) | -2.13 (-4.41, 0.17) | -1.21 (-3.46, 1.03) | -2.2 (-4.59, 0.2) | -1.77 (-4.27, 0.74) | **-2.15 (-4.29, -0.02)** | -2.14 (-7.86, 3.58) | -1.98 (-4.83, 0.86) | -2.79 (-5.93, 0.35) | Hesperidin |

Table 11 League table of HOMA-IR of different dietary polyphenols

| Placebo | Ellagic acid | Anthocyanin | Genistin/Genistein | Chlorogenic acid | Curcumin | Catechin | Resveratrol | Dihydromyricetin | Silymarin | Gallic acid and chlorogenic acid | Hesperidin |
| --- | --- | --- | --- | --- | --- | --- | --- | --- | --- | --- | --- |
| Placebo | -0.12 (-4.36, 4.14) | -0.01 (-5.98, 6.01) | -0.23 (-6.21, 5.74) | -0.51 (-4.77, 3.72) | -2.57 (-5.65, 0.4) | -0.33 (-4.57, 3.93) | -0.21 (-3.21, 2.79) | -0.9 (-6.95, 5.15) | -0.07 (-4.28, 4.17) | -0.34 (-6.29, 5.61) | 0.06 (-5.95, 6.08) |
| ＊0.12 (-4.14, 4.36) | Ellagic acid | 0.1 (-7.26, 7.47) | -0.12 (-7.45, 7.23) | -0.39 (-6.43, 5.66) | -2.45 (-7.73, 2.73) | -0.21 (-6.24, 5.8) | -0.1 (-5.32, 5.13) | -0.78 (-8.14, 6.56) | 0.05 (-5.94, 6.04) | -0.24 (-7.54, 7.13) | 0.18 (-7.18, 7.59) |
| 0.01 (-6.01, 5.98) | -0.1 (-7.47, 7.26) | Anthocyanin | -0.22 (-8.72, 8.24) | -0.49 (-7.88, 6.88) | -2.55 (-9.32, 4.07) | -0.32 (-7.66, 7.02) | -0.2 (-6.93, 6.51) | -0.89 (-9.42, 7.61) | -0.05 (-7.43, 7.33) | -0.34 (-8.84, 8.15) | 0.09 (-8.43, 8.55) |
| 0.23 (-5.74, 6.21) | 0.12 (-7.23, 7.45) | 0.22 (-8.24, 8.72) | Genistin/Genistein | -0.28 (-7.69, 7.1) | -2.32 (-9.08, 4.29) | -0.09 (-7.46, 7.25) | 0.03 (-6.7, 6.76) | -0.65 (-9.21, 7.79) | 0.17 (-7.16, 7.51) | -0.1 (-8.56, 8.35) | 0.3 (-8.18, 8.83) |
| 0.51 (-3.72, 4.77) | 0.39 (-5.66, 6.43) | 0.49 (-6.88, 7.88) | 0.28 (-7.1, 7.69) | Chlorogenic acid | -2.06 (-7.35, 3.15) | 0.18 (-5.83, 6.2) | 0.3 (-4.91, 5.51) | -0.39 (-7.78, 7) | 0.44 (-5.57, 6.45) | 0.16 (-7.14, 7.55) | 0.58 (-6.78, 7.91) |
| 2.57 (-0.4, 5.65) | 2.45 (-2.73, 7.73) | 2.55 (-4.07, 9.32) | 2.32 (-4.29, 9.08) | 2.06 (-3.15, 7.35) | Curcumin | 2.24 (-2.95, 7.51) | 2.36 (-1.86, 6.66) | 1.66 (-5, 8.48) | 2.5 (-2.64, 7.75) | 2.22 (-4.41, 8.99) | 2.64 (-4.05, 9.43) |
| 0.33 (-3.93, 4.57) | 0.21 (-5.8, 6.24) | 0.32 (-7.02, 7.66) | 0.09 (-7.25, 7.46) | -0.18 (-6.2, 5.83) | -2.24 (-7.51, 2.95) | Catechin | 0.12 (-5.08, 5.31) | -0.56 (-7.97, 6.8) | 0.27 (-5.74, 6.28) | -0.01 (-7.32, 7.35) | 0.4 (-6.96, 7.74) |
| 0.21 (-2.79, 3.21) | 0.1 (-5.13, 5.32) | 0.2 (-6.51, 6.93) | -0.03 (-6.76, 6.7) | -0.3 (-5.51, 4.91) | -2.36 (-6.66, 1.86) | -0.12 (-5.31, 5.08) | Resveratrol | -0.69 (-7.43, 6.04) | 0.14 (-5.03, 5.36) | -0.14 (-6.78, 6.56) | 0.28 (-6.42, 6.98) |
| 0.9 (-5.15, 6.95) | 0.78 (-6.56, 8.14) | 0.89 (-7.61, 9.42) | 0.65 (-7.79, 9.21) | 0.39 (-7, 7.78) | -1.66 (-8.48, 5) | 0.56 (-6.8, 7.97) | 0.69 (-6.04, 7.43) | Dihydromyricetin | 0.83 (-6.54, 8.25) | 0.55 (-7.91, 9.05) | 0.95 (-7.5, 9.46) |
| 0.07 (-4.17, 4.28) | -0.05 (-6.04, 5.94) | 0.05 (-7.33, 7.43) | -0.17 (-7.51, 7.16) | -0.44 (-6.45, 5.57) | -2.5 (-7.75, 2.64) | -0.27 (-6.28, 5.74) | -0.14 (-5.36, 5.03) | -0.83 (-8.25, 6.54) | Silymarin | -0.27 (-7.59, 7.02) | 0.13 (-7.26, 7.5) |
| 0.34 (-5.61, 6.29) | 0.24 (-7.13, 7.54) | 0.34 (-8.15, 8.84) | 0.1 (-8.35, 8.56) | -0.16 (-7.55, 7.14) | -2.22 (-8.99, 4.41) | 0.01 (-7.35, 7.32) | 0.14 (-6.56, 6.78) | -0.55 (-9.05, 7.91) | 0.27 (-7.02, 7.59) | Gallic acid and chlorogenic acid | 0.41 (-8.05, 8.88) |
| -0.06 (-6.08, 5.95) | -0.18 (-7.59, 7.18) | -0.09 (-8.55, 8.43) | -0.3 (-8.83, 8.18) | -0.58 (-7.91, 6.78) | -2.64 (-9.43, 4.05) | -0.4 (-7.74, 6.96) | -0.28 (-6.98, 6.42) | -0.95 (-9.46, 7.5) | -0.13 (-7.5, 7.26) | -0.41 (-8.88, 8.05) | Hesperidin |

# The abbreviation HOMA-IR stands for homeostasis model assessment of insulin resistance.＊denotes standardized mean difference (95% CI).
